# Efficacy-safety trade-off and patient selection: a meta-analysis informing clinical choice between CAR-T and bispecific antibodies for R/R B-NHL

**DOI:** 10.3389/fimmu.2026.1714145

**Published:** 2026-07-09

**Authors:** Jie Wang, Shifeng Hao, Mengke Huang, Shiji Wang, Yukai Duan, Mingzhi Zhang, Xudong Zhang, Hui Yu

**Affiliations:** Department of Oncology, The First Affiliated Hospital of Zhengzhou University, Zhengzhou, Henan, China

**Keywords:** bispecific antibodies, CAR-T therapy, efficacy-safety trade-offs, indirect comparison, relapsed/refractory B-cell non-Hodgkin lymphoma

## Abstract

This meta-analysis compared chimeric antigen receptor T-cell (CAR-T) therapy and bispecific antibodies (BsAbs) for relapsed/refractory B-cell non-Hodgkin lymphoma (R/R B-NHL), focusing on efficacy and safety. We analyzed 59 phase I/II trials involving 2,914 patients. CAR-T achieved higher ORR (72% [95% CI 67–77%] vs. 50% [38–62%]) and CR (54% [49–59%] vs. 33% [23–46%]) than BsAbs. However, it was associated with higher rates of grade ≥3 CRS (8% [6–11%] vs. 4% [3–7%]), ICANS (12% [9–16%] vs. 6% [2–18%]), and neurotoxicity (8% [6–10%] vs. 6% [2–13%]). Among CAR-T constructs, dual-targeting products (CD19/20 and CD19/22) showed higher efficacy with more varied toxicity profiles; among BsAbs, CD3×CD20 had a more favorable safety profile relative to CD3×CD19. These results suggest CAR-T may be preferable when deep remission is the priority, whereas BsAbs could be a better fit for frail patients or those seeking outpatient care with lower toxicity risks. Treatment selection should be tailored to patient characteristics, including age, tumor burden, and comorbidities. Together, these results provide a comprehensive, evidence-based framework to guide individualized treatment and sequencing in clinical practice.

## Introduction

The management of relapsed/refractory B-cell non-Hodgkin lymphoma (R/R B-NHL) presents a complex treatment dilemma. After chemo-immunotherapy fails, patients generally face a poor prognosis, and they can differ considerably in disease biology, tumor burden, disease pace, comorbidities and frailty, organ reserve, number of prior therapies, and access to intensive care (1–4). Two T-cell–redirecting strategies, chimeric antigen receptor T cells (CAR-T) and bispecific antibodies (BsAbs), are now used in salvage therapy (5–7). Each offers distinct benefit–risk–logistics profiles: CAR-T can deliver deep, potentially curative remissions but requires leukapheresis, manufacturing time, specialized centers, and tolerance for cytokine release syndrome (CRS) and immune effector cell-associated neurotoxicity syndrome (ICANS) (12, 13). BsAbs are “off-the-shelf,” given with step-up dosing, can be administered in outpatient settings, and have a reversible mechanism; the trade-off may be somewhat less depth or durability of response, but with improved feasibility and retreatability (14–16). For a given patient, the question is less about which treatment is globally “better” and more about which one best fits that patient’s current goals, risks, and constraints.

Current evidence syntheses map products and outcomes but provide limited decision-grade guidance. Narrative reviews often cover mechanisms and possible sequencing strategies but lack quantitative comparisons across different product designs or patient scenarios (17, 18). Indirect comparisons exist but commonly center on single products or select populations, constraining generalizability and leaving unanswered how construct features (target, co-stimulation, generation) and patient factors (fitness, tumor kinetics, CNS risk, bridging needs) should sway the choice at the bedside (19–21). Evidence on post-failure sequencing (e.g., BsAb after CAR-T failure and vice versa) is fragmented, further complicating pathway planning (21). Consequently, real-world decisions often rely on local experience rather than a transparent, quantitative efficacy–safety trade-off.

To address this gap, we carried out a PRISMA-guided, large-scale single-arm meta-analysis of CAR-T and BsAb trials in R/R B-NHL, synthesizing response and toxicity outcomes overall and by decision-relevant factors such as construct generation, target, and co-stimulation. Our aim is not to declare a single “winner,” but to quantify the trade-offs involved and translate them into signals that can help with patient selection—specifically, to clarify when the deeper responses expected with CAR-T justify its toxicity and logistical burden, and when the easier deployability and reversibility of BsAbs better match patient priorities. We hope this decision-oriented framework can support individualized treatment and rational sequencing in everyday practice.

## Methods

### Literature search strategy

This systematic review and meta-analysis was registered with PROSPERO (CRD420251039030) and conducted in accordance with the PRISMA statement. A completed PRISMA checklist is available in **Supplementary Table 1**. A systematic literature search was conducted in PubMed, Embase, Web of Science, and the Cochrane Library from database inception to February 8, 2025, and was restricted to English-language publications. The search terms included “CAR-T therapy,” “bispecific antibodies,” “non-Hodgkin lymphoma,” “diffuse large B-cell lymphoma,” “efficacy,” “safety,” “adverse events,” and “clinical trials.” The detailed search strategies for each database are provided in **Supplementary Table 2**. In addition, we manually screened abstracts from the American Society of Hematology (ASH) Annual Meeting and the European Hematology Association (EHA) Annual Congress, and checked reference lists for inclusion to ensure the comprehensiveness of the literature.

### Inclusion and exclusion criteria

We included prospective phase I/II trials that evaluated the efficacy and safety of BsAbs or CAR-T cell therapy at defined therapeutic doses in patients with R/R B-NHL. The following types of studies were excluded: (1) studies that did not involve patients with relapsed/refractory B-cell non-Hodgkin lymphoma; (2) randomized controlled trials, comparative studies, retrospective studies, case reports, and reviews; (3) studies that provided only abstracts or lacked raw data on primary outcome metrics (e.g., ORR, CR, PR, and adverse events); (4) studies that involved non-CAR-T/BsAb interventions or combination therapies without single-agent data; (5) studies with subjects aged < 18 years; (6) studies evaluating re-treatment after prior receipt of a drug with a similar mechanism; (7) or lacked information on prior treatment lines or treatment course.

### Data extraction

Two investigators (W.J. and H.S.) independently extracted the following information from the included studies: trial name, clinical trial registration number (NCT), publication journal, publication year, first author, trial phase, country, treatment regimen, sample size, and median follow-up time. Clinical characteristics included median age, prior lines of therapy, co-stimulation domains, product generation, and bridging chemotherapy for CAR-T studies. Additional baseline characteristics included the proportions of patients with double-hit/triple-hit rearrangements, stage III/IV, prior autologous stem cell transplantation (ASCT), refractory disease to the most recent line of therapy, and prior CAR-T exposure for BsAb studies.

### Outcomes

The primary outcome was objective response rate (ORR). Secondary outcomes included complete response (CR) and grade ≥3 adverse events, including CRS, neurological events, infections, and ICANS.

### Statistical analysis and quality assessment

Pooled analyses were performed using a random-effects model, with τ² estimated using the DerSimonian–Laird method. Subgroup analyses were conducted to compare differences in primary and secondary outcomes between treatment types, and between-group heterogeneity was assessed using the Q statistic. Sensitivity analysis was performed using a leave-one-out approach to evaluate the influence of individual studies on the pooled estimates and heterogeneity. Publication bias was assessed via funnel plots and Egger’s test, with trim-and-fill adjustment applied if bias was indicated. Quantitative synthesis and meta-regression were performed using R Studio (version 4.3.2).

Two reviewers (W.J. and H.S.) independently assessed the methodological quality of the included studies using the Methodological Index for Non-Randomized Studies (MINORS), and disagreements were resolved through discussion.

## Results

### Study characteristics and quality assessment

**Figure 1:**
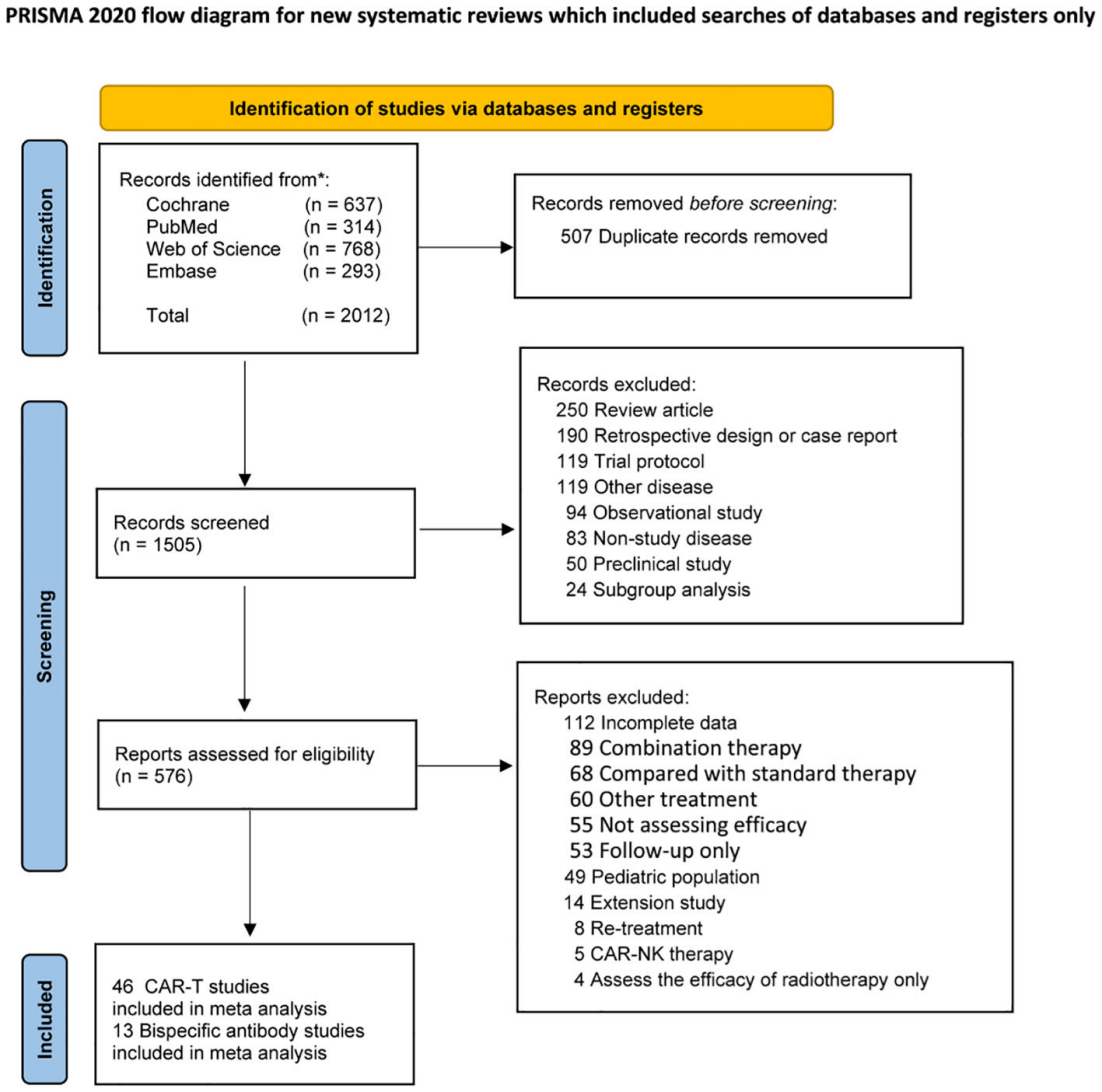
Literature search flow diagram. PRISMA 2020 flow diagram for new systematic reviews which included searches of databases and registers only. This diagram outlines the study selection process for the meta-analysis, starting with the identification of records from four databases (Cochrane, PubMed, Web of Science, and Embase), resulting in 2012 records. After removing 507 duplicate records, 1505 records were screened. Of these, 576 reports were assessed for eligibility. A total of 46 CAR-T studies and 13 bispecific antibody studies were included in the meta-analysis. The diagram also shows the reasons for exclusion during each phase of screening, such as review articles, trial protocols, observational studies, and studies not assessing efficacy. Reports excluded at the final stage include incomplete data, combination therapy studies, studies comparing with standard therapy, pediatric populations, and those assessing the efficacy of radiotherapy only.

The 59 included studies comprised 2914 patients, with 1893 in the CAR-T group and 1021 in the BsAbs group, yielding an approximate 2:1 ratio (**Figure 1**). Baseline characteristics of all included studies are summarized in **Table 1**. Detailed study-level baseline characteristics and treatment-related information for each included study are provided in **Supplementary Table 4**. The studies were published between 2021 and 2025, including 15 multicenter trials (25.4%) and 44 single-center trials (74.6%). Study phases comprised 24 phase I trials (40.7%), 19 phase II trials (32.2%), and 16 early-to-late phase transition studies (27.1%). Methodological quality assessed by the MINORS tool ranged from 13 to 16 (out of 16). A summary of the item-level assessment is shown in **Supplementary Figure 1**. The main reasons for score deduction were inadequate reporting of consecutive inclusion, unbiased assessment of the study endpoint, and failure to report prospective calculation of study size. A graphical summary of the study design and main findings is presented in **Supplementary Figure 2**, the distribution of median age between CAR-T and bispecific antibody groups is shown in **Supplementary Figure 3**, and detailed MINORS scores for each study are provided in **Supplementary Table 3**.

**Table 1:** Summary comparison of baseline characteristics between CAR-T and bispecific antibody studies.

Baseline characteristic	CAR-T	Bispecific antibodies
No. of studies	46	13
Total patients	1,893	1,021
Study phase	Phase I: 20; phase II: 15; phase I/II: 11	Phase I: 4; phase II: 4; phase I/II: 5
Study region	Multicenter: 7; China: 19; America/USA: 12; other: 8	Multicenter: 9; China: 1; America/USA: 1; other: 2
Age, median years	57 (36-74)	65 (56-72)
Prior lines of therapy, median	3 (1-8)	3 (2-4)
ECOG 0-2, n/N (%)	1,356/1,412 (96.0%)	697/808 (86.3%)
IPI >=3, n/N (%)	392/793 (49.4%)	126/268 (47.0%)
Ann Arbor stage III/IV, n/N (%)	786/982 (80.0%)	726/915 (79.3%)
Double-/triple-hit, n/N (%)	266/1,311 (20.3%)	71/488 (14.5%)
Prior ASCT, n/N (%)	464/1,471 (31.5%)	115/725 (15.9%)
Refractory to last therapy, n/N (%)	1,137/1,669 (68.1%)	708/939 (75.4%)
Disease status before infusion/treatment: CR+PR, n/N (%)	87/673 (12.9%)	38/860 (4.4%)
Target distribution, patients	CD19: 1,442; CD19/20: 211; CD19/22: 183; CD20: 57	CD3xCD20: 835; CD3xCD19: 186
CAR-T source	Autologous: 44 studies; Allogeneic: 2 studies	NA
Bridging therapy allowed	25/46 studies	NA
Prior CAR-T exposure, n/N (%)	NA	221/788 (28.0%)
Combination with other anticancer agents	NA	2/13 studies
Co-stimulatory domain(s)	4-1BB/CD3ζ: 20; CD28/CD3ζ: 8; 4-1BB: 7; CD28: 4; 4-1BB/CD28: 3; other: 4	NA

### Treatment characteristics

**Figure 2:**
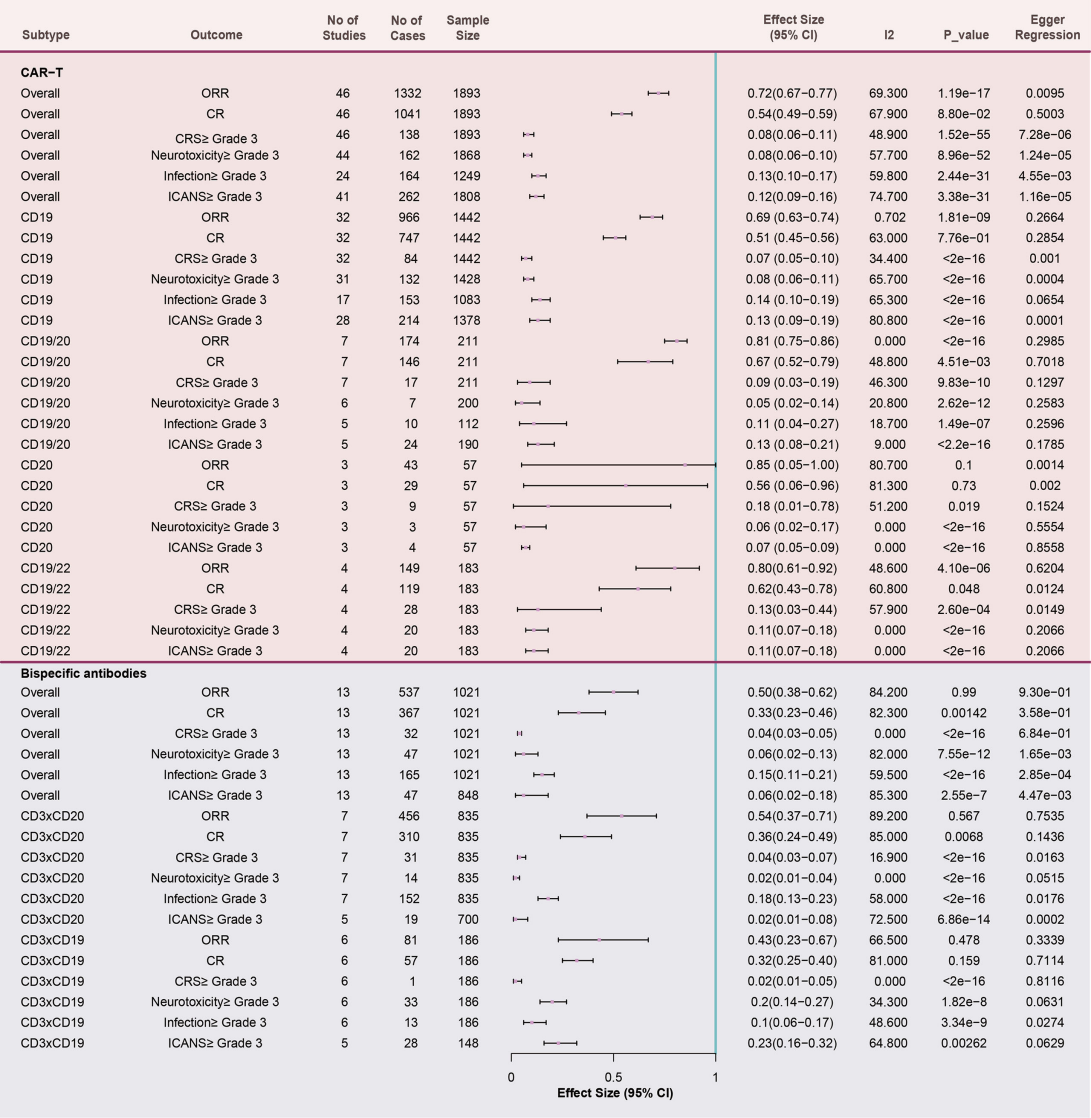
Pooled analysis of efficacy and safety outcomes for CAR-T cell therapies and BsAbs. Pooled analysis of efficacy and safety outcomes for CAR-T cell therapies and BsAbs. This forest plot presents the effect sizes (with 95% CI) for various outcomes associated with CAR-T therapies and BsAbs across multiple subtypes. The outcomes include ORR, CR and safety metrics such as CRS ≥ Grade 3, neurotoxicity ≥ Grade 3, infection ≥ Grade 3, and ICANS ≥ Grade 3. The data are stratified by therapy subtype (e.g., CD19, CD20, CD19/20, and CD19/22 for CAR-T therapies, and various combinations for BsAbs). The I² values and corresponding p-values are displayed to indicate heterogeneity and statistical significance, respectively. The overall efficacy and safety data for each treatment group are summarized, with the range of effect sizes provided for each outcome.

Among CAR-T studies, the most common target was CD19 (69.6%), followed by dual-targeting of CD19/CD20 (15.2%), CD20 (6.5%), and CD19/CD22 (8.7%). Specific products included axi-cel (n=241), liso-cel (n=422), relma-cel (n=94), tisa-cel (n=212), and others (n=924). Among BsAbs studies, CD3×CD20 was the predominant target (57.1%), with CD3×CD19 accounting for 42.9%. The evaluated agents were blinatumomab (n=165), mosunetuzumab (n=88), glofitamab (n=155), odronextamab (n=285), and others (n=328). An overview of the pooled efficacy and safety outcomes for both CAR-T therapies and BsAbs is presented in **Figure 2**.

### Efficacy outcomes

**Figure 3:**
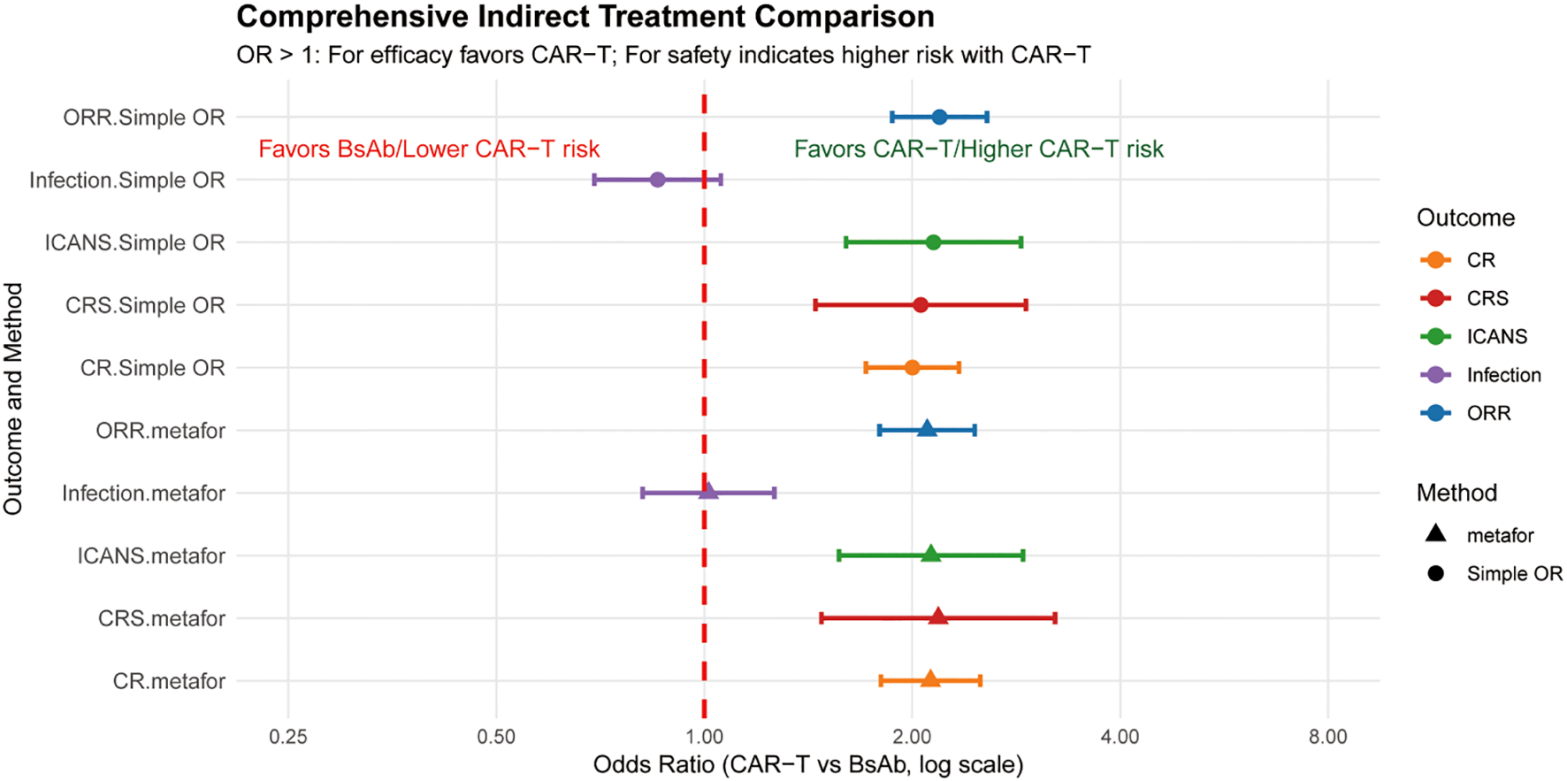
Indirect comparisons between treatments comparisons among CAR-T targets. Comprehensive Indirect Treatment Comparison for CAR-T versus bispecific antibodies (BsAb). This plot presents odds ratios (ORs) comparing CAR-T with bispecific antibody therapies across multiple efficacy and safety outcomes, displayed on a log scale. The outcomes include ORR, CR, CRS, infection, and ICANS. For each outcome, the ORs are derived using both simple OR and meta-analysis (metafor) methods, with results shown as triangles (metafor) and circles (simple OR). The red dashed line at OR = 1.0 indicates no difference between the therapies. ORs greater than 1 favor CAR-T and ORs less than 1 favor BsAb, with the direction of the comparison indicated in the color-coded legend. The outcomes show varying trends in efficacy and safety, with CAR-T showing higher risk for some safety-related outcomes and BsAb favoring lower risk for infection.

As shown in **Figure 3**, the overall ORR for CAR-T therapy was 72% (95% CI: 67-77%), compared to 50% (95% CI: 38-62%) for BsAbs. Among CAR-T constructs, response rates varied: CD19 CAR-T achieved an ORR of 69%, CD19/20 CAR-T 81%, and CD19/22 CAR-T 80%. BsAbs constructs showed lower ORRs overall, with both CD3×CD20 and CD3×CD19 demonstrating rates of approximately 50%. After correcting for publication bias using the trim-and-fill method, the pooled ORR for CAR-T decreased to 71% (95% CI: 66-75%). The overall CR rate for CAR-T therapy was 54% (95% CI: 49-59%), compared to 33% (95% CI: 23-46%) for BsAbs. Among CAR-T constructs, CD19/20 CAR-T achieved a CR rate of 67% and CD19 CAR-T achieved 51%. BsAb constructs showed consistently lower CR rates across different targets.

### Safety outcomes

#### CRS (Grade ≥3)

The pooled incidence of grade ≥3 CRS was 8% (95% CI: 6-11%) for CAR-T therapy, compared with 4% (95% CI: 3-7%) for BsAbs. Among CAR-T studies, the incidence varied by target: 7% (95% CI: 5-10%) for CD19-targeted CAR-T, 9% (95% CI: 3-19%) for CD19/20 dual-targeted CAR-T, 13% (95% CI: 3-44%) for CD19/22 dual-targeted CAR-T, and 18% (95% CI: 1-78%) for CD20-targeted CAR-T. For BsAbs, CRS incidence was 4% (95% CI: 3-7%) in the CD3×CD20 group and 2% (95% CI: 1-5%) in the CD3×CD19 group. Egger regression indicated significant publication bias for CAR-T studies (p = 0.00000728); after trim-and-fill correction, the incidence increased to 12% (95% CI: 9-15%). Detailed Egger regression and sensitivity analysis plots for each CAR-T outcome are presented in **Supplementary Figures 4.1–4.6**. No significant publication bias was detected for BsAbs (p = 0.684). Corresponding Egger regression and sensitivity analysis plots are shown in **Supplementary Figures 5.1–5.6**.

#### Neurotoxicity (Grade ≥3)

The incidence of grade ≥3 neurotoxicity was 8% (95% CI: 6-10%) for CAR-T therapy and 6% (95% CI: 2-13%) for BsAbs. In CAR-T subgroups, the incidence was 8% (95% CI: 6-11%) in CD19-targeted CAR-T, 5% (95% CI: 2-14%) in CD19/20 dual-targeted CAR-T, 6% (95% CI: 2-17%) in CD20-targeted CAR-T, and 11% (95% CI: 7-18%) in CD19/22 dual-targeted CAR-T. For BsAbs, the incidence was 2% (95% CI: 1-4%) in the CD3×CD20 group and 20% (95% CI: 14-27%) in the CD3×CD19 group. After trim-and-fill correction for publication bias, the incidence increased to 11% (95% CI: 8-14%) for CAR-T and 7% (95% CI: 3-15%) for BsAbs.

#### Infections (Grade ≥3)

The pooled incidence of grade ≥3 infections was 13% (95% CI: 10-17%) for CAR-T therapy and 15% (95% CI: 11-21%) for BsAbs. CAR-T subgroups showed incidences of 14% (95% CI: 10-19%) in CD19-targeted, 11% (95% CI: 4-27%) in CD19/20 dual-targeted, and 18% (95% CI: 13-23%) in CD3×CD20 BsAbs, with CD3×CD19 BsAbs showing an incidence of 10% (95% CI: 6-17%). Publication bias was detected in both groups; after trim-and-fill correction, the incidence increased to 15% (95% CI: 12-20%) for CAR-T and 18% (95% CI: 14-23%) for BsAbs.

#### ICANS (Grade ≥3)

The incidence of grade ≥3 ICANS was 12% (95% CI: 9-16%) for CAR-T therapy and 6% (95% CI: 2-18%) for BsAbs. In CAR-T subgroups, ICANS incidence was 13% (95% CI: 9-19%) for CD19-targeted, 13% (95% CI: 8-21%) for CD19/20 dual-targeted, and 7% (95% CI: 5-9%) for CD20-targeted CAR-T. For BsAbs, the incidence was 2% (95% CI: 1-8%) in the CD3×CD20 group and 23% (95% CI: 16-32%) in the CD3×CD19 group. After trim-and-fill correction, the incidence increased to 18% (95% CI: 13-23%) for CAR-T and 12% (95% CI: 4-30%) for BsAbs.

## Discussion

**Figure 4:**
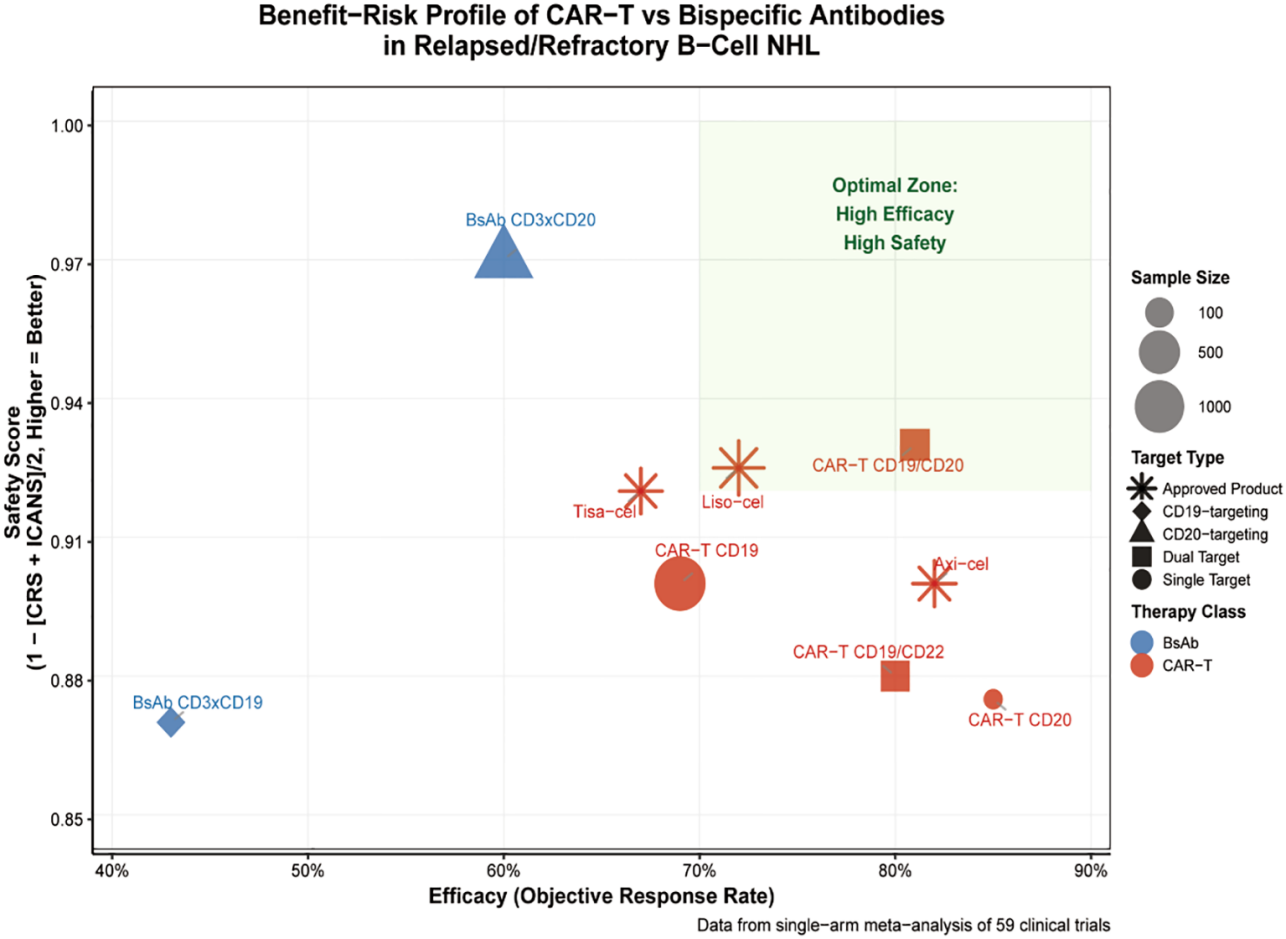
Benefit–risk analysis. Benefit–Risk Profile of CAR-T vs BsAbs in Relapsed/Refractory B-Cell NHL. This scatter plot compares the efficacy and safety profiles of various CAR-T and BsAbs therapies in R/R B-NHL. The x-axis represents efficacy (ORR), while the y-axis represents safety (a composite score of CRS and ICANS, where higher values indicate better safety). The size of each point corresponds to the sample size, with larger points representing studies with larger sample sizes (100, 500, and 1000). The color of the points indicates the target type (blue for BsAb and red for CAR-T), and the shape indicates whether the product is an approved product, single-target or dual-target. The optimal zone, indicating therapies with both high efficacy and high safety, is shaded in green. Notable CAR-T therapies such as axi-cel and tisa-cel are shown with larger circles, while BsAb therapies like BsAb CD3xCD19 and BsAb CD3xCD20 are positioned with diamond and triangle shapes. This plot is based on data from a single-arm meta-analysis of 59 clinical trial.

Beyond the individual comparisons of efficacy and safety outcomes, our analysis highlights the critical trade-off between them. As shown in **Figure 4**, when efficacy and safety are considered together, a more nuanced picture emerges regarding the relative roles of CAR-T and BsAbs in relapsed/refractory B-cell non-Hodgkin lymphoma. Our indirect treatment comparison suggests that CAR-T generally offers superior efficacy across multiple outcomes, particularly in ORR and CR, with higher odds ratios compared to BsAbs. However, CAR-T also carries higher risks, particularly for CRS, ICANS, and infection, where the odds ratios for these safety outcomes are significantly greater than 1. Although the overall rates of severe infections were similar between CAR-T and BsAbs in our analysis, the infection burden associated with CAR-T may be mechanistically distinct; sustained CAR-T-mediated lymphocyte aplasia and immunosuppression have been clinically shown to directly precipitate severe and late infectious events, and even autoimmune complications (8). This mechanistic insight reinforces the importance of enhanced infection surveillance and safety-optimization strategies in CAR-T recipients. In contrast, BsAbs demonstrate more favorable safety profiles, with lower incidences of CRS and ICANS, but comparatively lower efficacy, with odds ratios close to or below 1.

To further integrate efficacy and safety, we constructed a benefit–risk bubble plot incorporating ORR, CR, and grade ≥3 CRS and ICANS (**Figure 4**). In this integrated analysis, CD19/22 CAR-T occupied the most favorable efficacy–safety position, combining high efficacy with a relatively acceptable safety profile. CD19/20 CAR-T also showed strong efficacy, with slightly lower safety. Monovalent CD19 CAR-T (including tisa-cel and liso-cel) demonstrated an intermediate profile. Among BsAbs, CD3×CD20 BsAb was positioned in a region characterized by lower efficacy but higher safety. We further quantified the relative performance across all efficacy and safety outcomes using an indirect comparison summarized in a heatmap (**Supplementary Figure 6**). This visualization complements the benefit–risk bubble plot by providing a comprehensive overview of construct-level performance across both efficacy and safety dimensions.

Taken together, the advantage-risk bubble chart illustrates that CAR-T and BsAbs are not competing therapies but rather complementary tools that can be matched to patient characteristics. When the goal is maximal remission depth and durability—such as in patients with high tumor burden, younger age, or good organ reserve—CD19/22 or CD19/20 CAR-T may be preferred. When priority is placed on safety, tolerability, or treatment accessibility—for example, in elderly patients, those with multiple comorbidities, or those unable to wait for manufacturing—CD3×CD20 BsAb represents a reasonable choice, offering stable remission with low CRS and ICANS risk. Moreover, both modalities hold potential for sequential application (9, 10). BsAbs have been explored as bridging or holding therapy before CAR-T infusion, particularly for patients with rapidly progressive disease during the manufacturing interval, and retrospective studies together with recent meta-analytic evidence suggest that prior BsAb exposure does not appear to compromise subsequent CAR-T efficacy or safety (11, 12). Conversely, CD20×CD3 BsAbs have shown clinically meaningful activity as salvage therapy after CAR-T failure, although responses may be less favorable in patients with early relapse after CAR-T (13, 14). The retained efficacy of BsAbs after CD19-directed CAR-T may be partly explained by differences in target antigen and immune-engagement mechanism, while shared resistance factors should also be considered (15, 16). Therefore, CAR-T and BsAbs should not be viewed as mutually exclusive options but as components of a sequential therapeutic continuum.

The differences between CAR-T and BsAbs have clear biological and pharmacological foundations (15, 17, 18). CAR-T is a “living drug” capable of *in vivo* expansion, self-replication, and long-term persistence—key mechanisms underlying its higher efficacy but also contributing to greater inflammatory activation and higher risks of CRS and ICANS (19–21). In contrast, BsAbs are off-the-shelf agents with activation intensity that can be more readily controlled by dosage and administration, thereby maintaining lower levels of neurotoxicity and systemic inflammation (22–24). This observation is consistent with previous evidence linking CAR-T-associated neurotoxicity to robust immune effector activation, cytokine release, endothelial activation, blood-brain barrier disruption, and CAR-mediated immune synapse formation, rather than to antigen target alone (25–27).

In conclusion, rather than viewing CAR-T and BsAbs as competing strategies, they should be considered flexible therapeutic tools that can be combined across different disease stages and patient characteristics. The findings of this study provide an evidence base for developing individualized treatment strategies centered on the efficacy–risk balance.

### Research limitations

This study has several limitations. First, comparisons between CAR-T and BsAbs were largely indirect due to the absence of head-to-head randomized controlled trials, which may introduce residual confounding. Second, some early-phase studies had small sample sizes, leading to uncertainty in effect estimates for individual targets. Third, heterogeneity may have been influenced by differences across studies in patient eligibility criteria, disease burden, prior treatment exposure, and adverse event assessment systems. Exploratory meta-regression analyses (**Supplementary Figure 7**) did not identify age, refractory status, or prior treatment intensity as significant contributors to heterogeneity, suggesting that remaining variability may be driven by study-level or product-level differences rather than baseline patient characteristics. Finally, real-world evidence remains limited, and further data are needed to validate the external generalizability of these findings.

### Future research directions

Future research should focus on the following areas: (1) conducting head-to-head randomized controlled trials comparing CAR-T therapy with BsAbs to obtain more definitive comparative evidence; (2) developing predictive models based on target structure, biomarkers, and toxicity patterns to identify high-risk populations in advance; (3) strengthening real-world studies and longitudinal follow-up to evaluate long-term survival, sequential therapy strategies, and patient-reported outcomes; and (4) exploring optimal treatment sequencing, combination strategies, and novel target designs to further optimize the therapeutic landscape for relapsed/refractory B-cell non-Hodgkin lymphoma.
