# Supplementary material for: Efficacy-safety trade-off and patient selection: a meta-analysis informing clinical choice between CAR-T and bispecific antibodies for R/R B-NHL
Source: Front Immunol. 2026 Jul 9;17:1714145. doi: 10.3389/fimmu.2026.1714145 (PMC13391900; doi:10.3389/fimmu.2026.1714145)

**Supplemental Materials**

**Supplemental Table Legends**

**Table S1 The PRISMA checklist**

**Table S2 Search Strategy**

**Table S3 Detailed MINORS scores of included studies**

**Table S4 Study-level baseline characteristics of included studies**

**Table S5 Summary of pairwise comparisons for key clinical outcomes across CAR-T and bispecific antibody (BsAbs) subgroups.**

**Figure legends**

**Figure S1 A summary of the item-level assessment (MINORS tool)**

**Figure S2 Graphical abstract**

**Figure S3 Distribution of Median Age in CAR-T vs BsAbs Groups**

**Figure S4 CAT-T Egger Regression and sensitivity analysis**

**Figure S4.1 Overall CAR-T ORR**

**Figure S4.2 Overall CAR-T CR**

**Figure S4.3 Overall CAR-T CRS≥ Grade 3**

**Figure S4.4 Overall CAR-T Neurotoxicity≥ Grade 3**

**Figure S4.5 Overall CAR-T Infection≥ Grade 3**

**Figure S4.6 Overall CAR-T ICANS≥ Grade 3**

**Figure S5 Bispecific antibodies Egger Regression and sensitivity analysis**

**Figure S5.1 Overall Bispecific antibodies ORR**

**Figure S5.2 Overall Bispecific antibodies CR**

**Figure S5.3 Overall Bispecific antibodies CRS≥ Grade 3**

**Figure S5.4 Overall Bispecific antibodies Neurotoxicity≥ Grade 3**

**Figure S5.5 Overall Bispecific antibodies Infection≥ Grade 3**

**Figure S5.6 Overall Bispecific antibodies ICANS≥ Grade 3**

**Figure S6 Meta-regression Results**

**Figure S7 The heatmap of indirect comparison**

| **Section and Topic** | **Item** | **Checklist item** | **Location where item is reported** |
| --- | --- | --- | --- |
| **TITLE** | | |  |
| Title | 1 | Identify the report as a systematic review and a meta-analysis. | p1 |
| **ABSTRACT** | | |  |
| Abstract | 2 | Specify the background, methods, results, and conclusion. | p2-3 |
| **INTRODUCTION** | | |  |
| Rationale | 3 | Explained the underlying justification for conducting the review, considering the current state of knowledge in the field. | p2-3 |
| Objectives | 4 | Specified the central objective that guide the focus of the review in the introduction. | p2-3 |
| **METHODS** | | |  |
| Eligibility criteria | 5 | Describe the criteria used to include or exclude studies. | p3-4 |
| Information sources | 6 | Eligible studies were identified through systematic searches of PubMed, Embase, Cochrane Library, Web of Science, and other sources, with the last search date recorded for each database to ensure completeness. | p3-4 |
| Search strategy | 7 | Described in the supplementary Table S1 | Table S1 |
| Selection process | 8 | Two reviewers independently screened titles, abstracts, and full texts, with discrepancies resolved by a third reviewer. Searches were conducted in PubMed, Embase, Cochrane Library, Web of Science, and other sources. No automation tools were used. | p4, Figure 1 |
| Data collection process | 9 | Elucidate how data were collected, specifying the number of reviewers, any contact with study investigators to verify data. | p5 |
| Data items | 10 | Clarified whether all relevant results outcome were included, and described the data required for extraction under each outcome. | p5 |
| Study risk of bias assessment | 11 | Quality of included studies was assessed by two independent reviewers, with any disagreements resolved through discussion. Specific considerations during evaluation were documented as needed. | p5 |
| Effect measures | 12 | For each outcome, effect measures used in the synthesis included odds ratio, relative risk, weighted or simple mean difference, standard error, and adjusted regression coefficients. | p5 |
| Synthesis methods | 13 | Data from each eligible meta-analysis were reanalyzed according to predefined synthesis criteria, with relevant subgroups combined when appropriate. | p5 |
| Reporting bias assessment | 14 | Describe the method used to assess risk of bias in a synthesis. | p5 |
| Certainty assessment | 15 | Describe the methods used to assess evidence quality for included outcomes. | p5 |
| **RESULTS** | | |  |
| Study selection | 16 | A summary of the search and selection process was presented, including the number of records retrieved and studies finally included, preferably illustrated with a flow diagram. | Figure 1 |
| Study characteristics | 17 | List all included studies along with their key characteristics. | Table 1 |
| Risk of bias in studies | 18 | The risk of bias assessment results were reported for each included study. | Figure S3 |
| Results of individual studies | 19 | For each outcome, provide a pooled effect sizes with 95% confidence interval, preferably in figures and tables. | Figure 2 |
| Results of syntheses | 20 | For each synthesis, we summarized study characteristics and risk of bias, and reported statistical results including summary estimates with 95% confidence intervals and heterogeneity measures, preferably displayed in figures and tables. | Figure 2 |
| Reporting biases | 21 | Described the bias evaluation results for each included study and list potential bias that may influence the results, such as exposure measurements. | Figure S2 |
| **DISCUSSION** | | |  |
| Discussion | 23a | Findings were discussed in light of the existing body of evidence. | p9 |
|  | 23b | Discuss limitations of the included studies and the review methods. | p11 |
|  | 23c | Highlight implications for practice, policy, and future research. | p11 |
| **OTHER INFORMATION** | | |  |
| Registration and protocol | 24 | The study was registered in PROSPERO before initiation, and the registration number was provided. | p3 |
| Support | 25 | This work was supported by the Baiqiuen (Bethune) Excellence Medical Research Fund. The funder had no role in study design, data collection and analysis, decision to publish, or preparation of the manuscript. | p12 |
| Competing interests | 26 | None. | p12 |
| Availability of data, code and other materials | 27 | Data supporting the findings of this study are available from the corresponding authors upon reasonable request. | p12 |

**Table S1 PRISMA Checklist**

| **Pubmed** |  |  |
| --- | --- | --- |
| **NO.** | **Query** | **Records** |
| #1 | ("CAR-T therapy"[Title/Abstract] OR "Chimeric Antigen Receptor T-cell therapy"[Title/Abstract] OR "Axicabtagene ciloleucel"[Title/Abstract] OR "Tisagenlecleucel"[Title/Abstract] OR "Lisocabtagene maraleucel"[Title/Abstract] OR "CD19 CAR-T"[Title/Abstract] OR "CAR T-cell therapy"[Title/Abstract]) | 3658 |
| #2 | ("Bispecific Antibodies"[Mesh] OR "Bispecific T-cell Engager"[Title/Abstract] OR "CD19-CD3 bispecific antibody"[Title/Abstract] OR "Blincyto"[Title/Abstract] OR "Teclistamab"[Title/Abstract] OR "Elranatamab"[Title/Abstract] OR "BCMA/CD3 bispecific antibody"[Title/Abstract] OR "Linvoseltamab"[Title/Abstract]) | 291 |
| #3 | ("Diffuse Large B-cell Lymphoma"[Mesh] OR "DLBCL"[Title/Abstract] OR "Large B-cell lymphoma"[Title/Abstract] OR "Non-Hodgkin lymphoma"[Title/Abstract] OR "B-cell lymphoma"[Title/Abstract] OR "Refractory large B-cell lymphoma"[Title/Abstract] OR "Relapsed DLBCL"[Title/Abstract]) | 2134 |
| #4 | ("Efficacy"[Title/Abstract] OR "Safety"[Title/Abstract] OR "Adverse Events"[Title/Abstract] OR "Complete Response"[Title/Abstract] OR "Objective Response Rate"[Title/Abstract] OR "Cytokine Release Syndrome"[Title/Abstract] OR "Neurotoxicity"[Title/Abstract] OR "Side effects"[Title/Abstract] OR "Toxicity"[Title/Abstract]) | 297181 |
| #5 | ("clinical trial"[Publication Type] OR "phase II"[Title/Abstract] OR "phase III"[Title/Abstract] OR "phase I"[Title/Abstract] OR "clinical trials"[Title/Abstract] OR "clinicaltrials.gov"[Title/Abstract]) | 1015054 |
|  | (#1 OR#2) AND #3 AND #4 AND AND #5 | 314 |
|  |  |  |
| **Cochrane** |  |  |
| **NO.** | **Query** | **Records** |
| #1 | CAR-T therapy OR Chimeric Antigen Receptor T-cell therapy OR Axicabtagene ciloleucel OR Tisagenlecleucel OR Lisocabtagene maraleucel | 257 |
| #2 | Bispecific Antibodies OR Bispecific T-cell Engager OR CD19 and CD3 bispecific antibody OR Blincyto OR Teclistamab OR Elranatamab OR BCMA and CD3 bispecific antibody OR Linvoseltamab | 120 |
| #3 | Diffuse Large B-cell Lymphoma OR DLBCL OR Large B-cell lymphoma OR Non-Hodgkin lymphoma OR B-cell lymphoma OR Refractory large B-cell lymphoma OR Relapsed DLBCL | 4519 |
| #4 | clinical trial OR phase II OR phase III OR phase I OR clinical trials OR clinicaltrials.gov | 597199 |
| #5 | efficacy OR response rate OR clinical outcomes OR treatment safety OR adverse events | 277401 |
|  | (#1 OR#2) AND #3 AND #4 AND AND #5 | 637 |
|  |  |  |
| **Embase** |  |  |
| **NO.** | **Query** | **Records** |
| #1 | 'car t-cell therapies':ab,ti OR 'chimeric antigen receptor t-cell therapy':ab,ti OR 'Axicabtagene ciloleucel':ab,ti OR 'Tisagenlecleucel':ab,ti OR 'Lisocabtagene maraleucel':ab,ti | 6750 |
| #2 | 'Bispecific Antibodies':ab,ti OR 'Bispecific T-cell Engager':ab,ti OR 'CD19-CD3 bispecific antibody':ab,ti OR 'Blincyto':ab,ti OR 'Teclistamab':ab,ti OR 'Elranatamab':ab,ti OR 'BCMA/CD3 bispecific antibody':ab,ti OR 'Linvoseltamab':ab,ti | 5434 |
| #3 | large b-cell lymphoma':ab,ti OR 'non-hodgkin lymphoma':ab,ti OR 'b-cell lymphoma':ab,ti OR 'Diffuse Large B-cell Lymphoma'/exp | 95579 |
| #4 | 'clinical trial':ab,ti OR 'randomized controlled trial':ab,ti OR 'phase II':ab,ti OR 'phase III':ab,ti OR 'clinicaltrials.gov':ab,ti | 688696 |
| #5 | 'efficacy':ab,ti OR 'response rate':ab,ti OR 'treatment outcome':ab,ti OR 'adverse events':ab,ti OR 'safety':ab,ti | 2802926 |
|  | (#1 OR#2) AND #3 AND #4 AND #5 | 293 |
|  |  |  |
| **Web of Science** |  |  |
| **NO.** | **Query** | **Records** |
| #1 | TS=("CAR-T Therapy" OR "Chimeric Antigen Receptor T-cell Therapy" OR "Axicabtagene ciloleucel" OR "Tisagenlecleucel" OR "Lisocabtagene maraleucel" OR "CD19 CAR-T" OR "CAR T-cell Receptors" OR "Chimeric T-cell Receptors" OR "T-cell Therapy") | 27442 |
| #2 | TS=("Bispecific Antibodies" OR "Bispecific T-cell Engager" OR "CD19-CD3 Bispecific Antibody" OR "Blincyto" OR "Teclistamab" OR "Elranatamab" OR "Linvoseltamab" OR "Bispecific Monoclonal Antibodies" OR "Monoclonal Antibodies, Bispecific") | 7123 |
| #3 | TS=("Diffuse Large B-cell Lymphoma" OR "DLBCL" OR "Large B-cell Lymphoma" OR "Non-Hodgkin Lymphoma" OR "B-cell Lymphoma" OR "Refractory Large B-cell Lymphoma" OR "Relapsed DLBCL") | 115154 |
| #4 | TS=("clinical trial" OR "randomized controlled trial" OR "phase II" OR "phase III" OR "phase I" OR "clinicaltrials.gov") | 1211760 |
| #5 | TS=("efficacy" OR "response rate" OR "clinical outcomes" OR "treatment safety" OR "adverse events") | 3401168 |
|  | (#1 OR#2) AND #3 AND #4 AND AND #5 | 768 |

**Table S2 Search Strategy**

| Target | Registration number | Clearly stated aim | Inclusion of consecutive patients | Prospective data collection | Endpoints appropriate to the aim | Unbiased assessment of endpoint | Adequate follow-up period | Loss to follow-up less than 5% | Prospective calculation of study size | Total score (0–16) |
| --- | --- | --- | --- | --- | --- | --- | --- | --- | --- | --- |
| CD19 | NCT04531046 | 2 | 2 | 2 | 2 | 2 | 2 | 2 | 2 | 16 |
|  | NCT02348216 | 2 | 2 | 2 | 2 | 2 | 2 | 2 | 2 | 16 |
|  | NCT05108805 | 2 | 2 | 2 | 2 | 2 | 1 | 1 | 0 | 12 |
|  | NCT03761056 | 2 | 1 | 2 | 2 | 2 | 1 | 1 | 2 | 13 |
|  | JapicCTI-183914 | 2 | 1 | 2 | 2 | 2 | 2 | 2 | 2 | 13 |
|  | NCT03744676 | 2 | 1 | 2 | 2 | 2 | 2 | 1 | 2 | 14 |
|  | NCT03484702 | 2 | 1 | 2 | 2 | 2 | 2 | 1 | 2 | 14 |
|  | NCT02631044 | 2 | 1 | 2 | 2 | 2 | 2 | 1 | 1 | 13 |
|  | NCT03483103 | 2 | 1 | 2 | 2 | 1 | 2 | 2 | 1 | 13 |
|  | NCT04812691 | 2 | 2 | 2 | 2 | 2 | 2 | 2 | 2 | 16 |
|  | NCT03344367 | 2 | 2 | 2 | 2 | 2 | 2 | 2 | 2 | 16 |
|  | NCT04089215 | 2 | 2 | 2 | 2 | 2 | 2 | 2 | 0 | 14 |
|  | NCT02445248 | 2 | 2 | 2 | 2 | 2 | 2 | 2 | 1 | 15 |
|  | NCT03568461 | 2 | 2 | 2 | 2 | 2 | 2 | 2 | 1 | 15 |
|  | CTRI/2022/12/048211 | 2 | 1 | 2 | 2 | 1 | 2 | 1 | 1 | 12 |
|  | ChiCTR-OOC-16007779 | 2 | 1 | 2 | 2 | 1 | 2 | 2 | 1 | 13 |
|  | NCT03121625 | 2 | 1 | 2 | 2 | 1 | 2 | 2 | 1 | 13 |
|  | NCT03156101 | 2 | 1 | 2 | 2 | 1 | 1 | 2 | 0 | 11 |
|  | NCT02842138 | 2 | 1 | 2 | 2 | 1 | 1 | 2 | 1 | 12 |
|  | NCT03960840 | 2 | 1 | 2 | 2 | 1 | 2 | 2 | 1 | 13 |
|  | NCT04035434 | 2 | 1 | 2 | 2 | 1 | 2 | 2 | 1 | 13 |
|  | NCT03666000 | 2 | 1 | 2 | 2 | 1 | 1 | 2 | 1 | 12 |
|  | NCT03528421 | 2 | 1 | 2 | 2 | 1 | 1 | 2 | 1 | 12 |
|  | NCT04213469 | 2 | 1 | 2 | 2 | 1 | 2 | 2 | 1 | 13 |
|  | NCT03258047 | 2 | 2 | 2 | 2 | 2 | 1 | 1 | 0 | 12 |
|  | ChiCTR2100052677 and ChiCTR1900028692 | 2 | 1 | 2 | 2 | 1 | 2 | 2 | 1 | 13 |
|  | NCT04464200 | 2 | 2 | 2 | 2 | 2 | 1 | 1 | 0 | 12 |
|  | NCT04206943 | 2 | 2 | 2 | 2 | 2 | 1 | 1 | 0 | 12 |
|  | NCT01840566 | 2 | 1 | 2 | 2 | 1 | 2 | 2 | 1 | 13 |
|  | NCT02976857 | 2 | 1 | 2 | 2 | 1 | 1 | 2 | 1 | 12 |
|  | NCT02132624 | 2 | 1 | 2 | 2 | 1 | 2 | 2 | 1 | 13 |
|  | NCT02772198 | 2 | 1 | 2 | 2 | 1 | 1 | 2 | 0 | 11 |
| CD19/20 | NCT04317885 | 2 | 1 | 2 | 1 | 1 | 1 | 2 | 1 | 11 |
|  | NCT03207178 | 2 | 1 | 2 | 2 | 1 | 2 | 2 | 2 | 14 |
|  | NCT03019055 | 2 | 1 | 2 | 2 | 1 | 2 | 2 | 1 | 13 |
|  | NCT04007029 | 2 | 1 | 2 | 2 | 1 | 2 | 1 | 2 | 13 |
|  | NCT03870945 | 2 | 1 | 2 | 2 | 2 | 1 | 2 | 1 | 13 |
|  | NCT04723914 | 2 | 1 | 2 | 2 | 2 | 1 | 2 | 1 | 13 |
|  | NCT03097770 | 2 | 1 | 2 | 2 | 2 | 1 | 2 | 1 | 13 |
| CD20 | ChiCTR2000036350 | 2 | 1 | 2 | 2 | 2 | 1 | 2 | 1 | 13 |
|  | NCT03277729 | 2 | 1 | 2 | 2 | 2 | 2 | 1 | 2 | 14 |
|  | NCT02776813 | 2 | 1 | 2 | 2 | 1 | 1 | 2 | 1 | 12 |
| CD19/CD22 | NCT03196830 | 2 | 2 | 2 | 2 | 2 | 2 | 2 | 1 | 15 |
|  | NCT03233854 | 2 | 2 | 2 | 2 | 2 | 2 | 2 | 0 | 14 |
|  | NCT03289455 | 2 | 2 | 2 | 2 | 2 | 2 | 2 | 2 | 16 |
|  | ChiCTROPN16008526 | 1 | 1 | 2 | 2 | 2 | 1 | 2 | 2 | 13 |
| CD3**·**CD20 | NCT03888105 | 2 | 1 | 2 | 2 | 1 | 2 | 2 | 2 | 14 |
|  | NCT03625037 | 2 | 1 | 2 | 2 | 1 | 2 | 2 | 2 | 14 |
|  | NCT04663347 | 2 | 1 | 2 | 2 | 2 | 2 | 1 | 2 | 15 |
|  | NCT04923048 | 2 | 2 | 2 | 2 | 1 | 1 | 2 | 2 | 14 |
|  | NCT03075696 | 2 | 2 | 2 | 2 | 1 | 2 | 2 | 2 | 15 |
|  | NCT02500407 | 2 | 2 | 2 | 2 | 1 | 2 | 2 | 2 | 15 |
|  | NCT02290951 |  |  |  |  |  |  |  |  |  |
| CD3**·**CD19 | NCT00274742 | 2 | 1 | 2 | 2 | 2 | 2 | 2 | 1 | 14 |
|  | NCT03072771 | 2 | 1 | 2 | 2 | 2 | 2 | 2 | 1 | 14 |
|  | NCT03023878 | 2 | 2 | 2 | 2 | 2 | 2 | 2 | 2 | 16 |
|  | NCT02910063 | 2 | 2 | 2 | 2 | 2 | 2 | 2 | 2 | 16 |
|  | NCT01741792 | 2 | 1 | 2 | 2 | 2 | 1 | 2 | 1 | 13 |
|  | NCT04056975 | 2 | 1 | 2 | 2 | 2 | 1 | 2 | 1 | 13 |

**Table S3** Detailed MINORS scores of included studies

Note: MINORS items were scored as 0 (not reported), 1 (reported but inadequate), or 2 (reported and adequate). The maximum score for non-comparative studies was 16. Two reviewers independently assessed study quality, and disagreements were resolved by discussion.

| Target | Registration Number | Phase | Age (med, range) | ECOG 0–2, n (%) | IPI≥3 n (%) | Regime | Country | Total | DH/TH  (%) | Stage III/IV (%) | Prior lines (med, range) | ASCT (%) | Refractory (%) | Disease status before infusion | autologous or allogenic | Bridging Therapy n (%) | Gen | Co-stim domain(s) |
| --- | --- | --- | --- | --- | --- | --- | --- | --- | --- | --- | --- | --- | --- | --- | --- | --- | --- | --- |
| CD19 | NCT04531046(1) | II | 70(49-81) | 62(100) | 35(56.5) | axi-cel | France | 62 | NR | 74.2 | 1 | 3.2 | 54.8 | 19(30.7) | autologous | 52(83.9) | second | CD28 |
|  | NCT02348216(2) | II | 58(23-76) | 101(100) | 48(48) | axi-cel | multi-center | 108 | 29.6 | 79.6 | 3(1-10) | 19.4 | 72.2 | / | autologous | / | second | CD28 |
|  | NCT05108805(3) | I/II | 69(54-79) | 15(100) | / | axi-cel | America | 15 | NR | 93.3 | 2(1-4) | 0.0 | NR | / | autologous | / | second | CD28 |
|  | NCT03761056(4) | II | 61(23-86) | 40(100) | 31(78) | axi-cel | multi-center | 40 | 40 | 95.0 | 1 | NR | 40 | 21(53) | autologous | 7(17.5) | second | CD28 |
|  | JapicCTI-183914(5) | II | 58(44-70) | 16(100) | 6(37.5) | axi-cel | Japan | 16 | NR | 50.0 | 3 | 37.5 | 62.5 | / | autologous | / | second | CD28 |
|  | NCT03744676(6) | II | 66(28-86) | 82(100) | / | liso-cel | America | 82 | 18.3 | 34.4 | 2(2-6) | 15.9 | 91.5 | 7(9) | autologous | 44(54) | second | 4-1BB |
|  | NCT03484702(7) | II | 57(47-73) | 10(100) | / | liso-cel | Japan | 10 | NR | NR | 3(1-9) | 20.0 | 70.0 | / | autologous | 10(100) | second | 4-1BB |
|  | NCT02631044(8) | I/II | 63(18-86) | 269(100) | / | liso-cel | America | 269 | 13.4 | NR | 3(1-8) | 71.7 | 67.3 | / | autologous | 159(59) | second | 4-1BB |
|  | NCT03483103(9) | II | 74(53-84) | 61(100) | 10(16) | liso-cel | America | 61 | 32.8 | NR | ≥2 | NR | 54.1 | 0(0) | autologous | 32(52) | second | 4-1BB |
|  | NCT04812691(10) | I | 60(45-71) | / | / | liso-cel | China | 12 | NR | 75.0 | ≥2 | NR | 100.0 | / | autologous | / | second | 4-1BB |
|  | NCT03344367(11) | I | 57(29-68) | 23(100) | / | relma-cel | China | 23 | NR | NR | 2(2-5) | 8.7 | 100.0 | 0(0) | autologous | 8(34.8) | second | 4-1BB |
|  | NCT04089215(12) | I/II | 56(18-75) | 59(100) | 23(47.9) | relma-cel | China | 59 | 5.1 | NR | 2(2-7） | 10.2 | 81.4 | / | autologous | 26(44.2) | second | 4-1BB |
|  | NCT02445248(13) | II | 56 (22-76 ) | 111(100) | 58(52) | tisa-cel | multi-center | 115 | 27.0 | 75.7 | 3 (1-6) | 47.0 | 53.0 | 0(0) | autologous | 102(92) | second | 4-1BB |
|  | NCT03568461(14) | II | 57(49-64) | 97(100) | 58(60) | tisa-cel | multi-center | 97 | NR | 85.6 | 4(2-13) | 36.1 | 78.4 | 0(0) | autologous | 44(45) | second | 4-1BB |
|  | NCT04008251(15) | II | 43(16-71) | 47(100) | / | Actaly-cel | India | 47 | NR | NR | 2(1-6) | NR | 80.9 | / | autologous | / | second | CD28 |
|  | ChiCTR-OOC-16007779(16) | I | NR | 21(100) | 13(61.9) | 4SCAR19 | China | 21 | 5.0 | 95.2 | NR | 23.8 | 66.7 | / | autologous | 7(33.3) | forth | CD28 and CD27 |
|  | NCT03121625(17) | I | 49(29-69) | / | / | FMC63 scFv | China | 11 | 22.2 | 81.8 | 3(2-6) | NR | NR | / | autologous | / | second | 4-1BB |
|  | NCT03156101(18) | I | 45(18-59) | 14(100) | / | NA | China | 14 | NR | NR | 8(5-19) | 0.0 | 100.0 | / | autologous | / | second | 4-1BB |
|  | NCT02842138(19) | I | 53(24-76) | 25(100) | 10(40) | CD19-BBz(86) | China | 25 | 4.0 | 92.0 | 3(1-6) | 16.0 | 52.0 | / | autologous | / | second | 4-1BB |
|  | NCT03960840(20) | II | 64(26-81) | / | 24(38) | Rap-cel | multi-center | 63 | 25.0 | NR | 2(2-6) | 30.2 | 58.7 | / | autologous | / | second | 4-1BB |
|  | NCT04035434(21) | I | 64(25-75) | 32(100) | / | CTX110 | multi-center | 32 | NR | NR | 3(2-10) | 34.4 | 53.1 | / | autologous | / | first | CD28 |
|  | NCT03666000(22) | II | 59(34-76) | / | / | Azer-cel | America | 16 | NR | NR | 4(2-15) | 37.5 | 37.5 | / | allogenic | / | second | CD28 |
|  | NCT03528421(23) | I/II | 61(27-69) | 22(100) | / | IM19 | China | 22 | NR | NR | 2(1-4) | NR | 100.0 | / | autologous | / | first | 4-1BB |
|  | NCT04213469(24) | I | 56(46-62) | / | 15(71.4) | PD1-19bbz | China | 21 | NR | 90.5 | 5(3-5) | 19.0 | 100.0 | / | autologous | 10(48) | second | 4-1BB |
|  | NCT03258047(25) | I | 55(29-73) | 23(58.9) | 12(30.8) | NA | China | 39 | 2.6 | 66.7 | 3(1-6) | 7.7 | 51.3 | / | autologous | 0(0) | forth | 4-1BB |
|  | ChiCTR2100052677(26) | I | 47.7(21-70) | / | / | CXCR5 CD19 CAR-T | China | 10 | 30.0 | 10.0 | NR | 10.0 | 90.0 | / | autologous | 2(20) | second | 4-1BB |
|  | NCT04464200(27) | I | 62(48-86) | / | / | 19(T2)28z1xx CAR T | America | 28 | NR | NR | NR | 10.7 | 71.4 | / | autologous | / | third | CD28 |
|  | NCT04206943(28) | I/II | NA | / | / | ISIKOK-19 | Turkey | 9 | 11.1 | NR | NR | 44.4 | 100.0 | 0(0) | autologous | 2(50) | second | CD28 |
|  | NCT01840566(29) | I | NA | / | / | 19-28z CAR T | America | 17 | NR | NR | 3(2-8) | NR | NR | 15(100) | autologous | / | second | CD28 |
|  | NCT02976857(11) | I | 44.5(25.4-69.2) | 10(100) | / | C-CAR011 | China | 10 | NR | 80.0 | 3(1-5) | NR | 10.0 | / | autologous | / | second | 4-1BB |
|  | NCT02132624(30) | I/II | 61(24-71) | 11(100) | / | NA | Sweden | 15 | NR | NR | 4(1-7) | 20.0 | NR | 5(45.5) | autologous | 11(100) | third | CD28 and 4-1BB |
|  | NCT02772198(31) | I/II | 49(20-73) | 65(89.1) | / | POC | Israel | 73 | 9.0 | 72.6 | 3(2-6) | 39.7 | 51.4 | 10(13.4) | autologous | 6(8) | second | CD28 |
| CD19/20 | NCT04317885(32) | I | 55(25-71) | / | / | priz-cel | China | 48 | 31.3 | 75.0 | 3(1-7) | 16.7 | 70.8 | / | autologous | / | third | 4-1BB |
|  | NCT03207178(33) | II | 55 (23-72) | 21(100) | 15(71.4) | NA | China | 21 | 19.0 | 85.7 | 3 (1-6) | 4.0 | 71.4 | 0(0) | autologous | 0(0) | second | 4-1BB |
|  | NCT03019055(34) | I | 57(38-72) | / | / | LV20.19 | America | 22 | 23.0 | NR | 4(2-12) | 37.0 | 82.0 | / | autologous | / | second | 4-1BB |
|  | NCT04007029(35) | I | 58(34-70) | / | / | NA | America | 10 | 10.0 | 100.0 | 3(2-4) | 10.0 | 70.0 | 1(10) | autologous | 9(90) | second | 4-1BB |
|  | NCT03870945(36) | I | 72(20-78) | / | 7(58.3) | MB-CART2019.1 | Germany | 12 | NR | NR | NR | NR | 41.7 | / | autologous | / | second | 4-1BB |
|  | NCT04723914(37) | I/II | 59(43-68) | 11(100) | / | NA | China | 11 | 36.4 | 100.0 | NR | NR | 27.3 | / | autologous | / | second | 4-1BB |
|  | NCT03097770(38) | I/II | 50(17-68) | 87(100) | / | TanCAR7 T | China | 87 | 34.0 | 85.0 | 3(2-9) | 14.0 | 80.5 | / | autologous | / | second | 4-1BB |
| CD19/22 | NCT03196830(39) | II | 55(31-72) | / | 27(81.8) | NA | China | 33 | NR | 81.8 | 3(2-6) | NR | 78.8 | 9(27.3) | autologous | / | second | 4-1BB |
|  | NCT03233854(40) | I | 69(25-78) | / | / | CD19-22.BB.z-CAR | America | 38 | 81.8 | NR | NR | 10.6 | NR | / | autologous | 0(0) | third | 4-1BB |
|  | NCT03289455(41) | I/II | 57(28-83) | 6(100) | / | AUTO3 | multi-center | 23 | NR | NR | 3(2-10) | 17.4 | NR | / | autologous | / | third | 4-1BB |
|  | ChiCTROPN16008526(42) | I/II | 36(9-71) | / | / | CAR-22/19 cocktail | China | 89 | 4.5 | NR | NR | NR | NR | / | autologous | / | second | 4-1BB |
| CD20 | ChiCTR2000036350(43) | I | 48(30-66) | 15(100) | / | NA | China | 15 | NR | 73.3 | 4(2-8) | NR | 40.0 | 0(0) | autologous | 0(0) | second | 4-1BB |
|  | NCT03277729(44) | I/II | NR | / | / | MB-106 | America | 16 | NR | NR | NR | NR | NR | / | autologous | / | third | CD28 and 4-1BB |
|  | NCT02776813(45) | I | 64(36-71) | / | / | ACTR087 | America | 26 | NR | NR | 3(1-9) | NR | 84.6 | / | autologous | / | second | 4-1BB |
| CD3**·**CD20 | NCT03888105(46) | II | 66(24-88) | 481(100) | / | Odronextamab | multi-center | 140 | NR | 80.0 | 2(2-8) | NR | 65.7 | / | / | / | / | / |
|  | NCT03625037(47) | I/II | 64(20-83) | 67(98) | / | Epcoritamab | multi-center | 157 | 8.3 | 75.1 | 3(2-11) | 19.7 | 82.8 | 0(0) | / | / | / | / |
|  | NCT04663347(48) | I/II | 72(20-87) | 103(100) | 65(63.1) | Epcoritamab、GemOx | multi-center | 103 | 5.5 | 78.6 | 2(1-6) | 9.7 | 69.9 | 0(0) | / | / | / | / |
|  | NCT04923048(49) | I/II | 60(28-81) | / | / | GB261 | multi-center | 47 | NR | NR | 3(1-10) | NR | 70.2 | (0) | / | / | / | / |
|  | NCT03075696(50) | I/II | 66(21-90) | 154(100) | / | Glofitamab | multi-center | 155 | 12.9 | 74.8 | 3(2-7) | 18.0 | 85.1 | 0(0) | / | / | / | / |
|  | NCT02500407(51) | I/II | 66(24-96) | 88(100) | / | Mosunetuzumab | multi-center | 88 | 19.3 | 84.1 | 3(2-13) | 17.0 | 79.5 | 0(0) | / | / | / | / |
|  | NCT02290951(52) | I | 67(57-73) | / | / | Odronextamab | America | 145 | NR | 85.0 | 3(2-5) | 8.2 | 82.0 | 0(0) | / | / | / | / |
| CD3**·**CD19 | NCT00274742(53) | I | 60(40-80) | 76(100) | 5(7) | Blinatumomab | multi-center | 38 | NR | NR | 3(1-6) | 31.6 | 42.1 | 0(0) | / | / | / | / |
|  | NCT03072771(54) | I | 64(NR) | 14(100) | 10(71) | Blinatumomab | America | 14 | NR | 71.4 | 2(2-7) | 0 | NR | 14(100) | / | / | / | / |
|  | NCT03023878(55) | II | 64(35-81) | 28(100) | 26(92.9) | Blinatumomab | multi-center | 47 | 25.5 | 89.3 | NR | NR | NR | 24(85.7) | / | / | / | / |
|  | NCT02910063(56) | II | 56 (19-75) | 40(100) | 15(37) | Blinatumomab | multi-center | 41 | 22 | 73.1 | 2(NR) | NR | 68.3 | 0(0) | / | / | / | / |
|  | NCT01741792(57) | II | 66(34-85) | 25(100) | 5(20) | Blinatumomab | Germany | 25 | NR | 80.0 | 4(1-8) | 28.0 | 64.0 | 0(0) | / | / | / | / |
|  | NCT04056975(58) | I | NR | / | / | A-319 | China | 21 | NR | NR | NR | NR | NR | / | / | / | / | / |

**Table S4** **Study-level baseline characteristics of included studies**

**References**：

1. R H, E B, G C, FX G, F M, L O. Axicabtagene ciloleucel as second-line therapy in large B cell lymphoma ineligible for autologous stem cell transplantation: A phase 2 trial %J Nat Med. 2023;29(10):2593-601.

2. SS N, FL L, NL B, LJ L, DB M, CA J. Axicabtagene ciloleucel CAR T-cell therapy in refractory large B-cell lymphoma %J N Engl J Med. 2017;377(26):2531-44.

3. B D, ST B, VG P, E B, R J, S S. Feasibility of axicabtagene ciloleucel in the outpatient setting: Primary analysis of prospective trial %J Bone Marrow Transplant. 2025;60(6):769-72.

4. SS N, M D, J M, ML U, C T, OO O. Axicabtagene ciloleucel as first-line therapy in high-risk large B-cell lymphoma: The phase 2 ZUMA-12 trial %J Nat Med. 2022;28(4):735-42.

5. K K, S M, H G, J K, N F, K S. Phase 2 study of axicabtagene ciloleucel in japanese patients with relapsed or refractory large B-cell lymphoma %J Int J Clin Oncol. 2022;27(1):213-23.

6. Y L, CO F, M C, C B, M M, D H. OUTREACH: Phase 2 study of lisocabtagene maraleucel as outpatient or inpatient treatment at community sites for R/R LBCL %J Blood Adv. 2024;8(23):6114-26.

7. S M, G Y, D M, Y A-M, D K, R A. Phase 2 results of lisocabtagene maraleucel in japanese patients with relapsed/refractory aggressive B-cell non-hodgkin lymphoma %J Cancer Med. 2022;11(24):4889-99.

8. M W, T S, LI G, M K, M L, AV H. Lisocabtagene maraleucel in relapsed/refractory mantle cell lymphoma: Primary analysis of the mantle cell lymphoma cohort from TRANSCEND NHL 001, a phase I multicenter seamless design study %J J Clin Oncol Off J Am Soc Clin Oncol. 2024;42(10):1146-57.

9. A S, D H, PA R, N G, M H, GC H. Lisocabtagene maraleucel for R/R LBCL in patients not intended for HSCT: Final results of the phase 2 PILOT study %J Blood Adv. 2025;9(15):3694-705.

10. Y W, S Y, Y Y, P X, C X, C Z. Preliminary safety and efficacy of relmacabtagene autoleucel (relma-cel) in adults with moderately to severely active systemic lupus erythematosus: A phase I dose-escalation study %J J Autoimmun. 2025;157:103489.

11. M E, A O, B B, J C-V, M AEA, I M. Chimeric antigen receptor (CAR) T-cell therapy for people with relapsed or refractory diffuse large B-cell lymphoma %J Cochrane Database Syst Rev. 2021;2021(9):CD013365.

12. Z Y, H Y, Y G, W L, D Z, D Z. Relmacabtagene autoleucel (relma-cel) CD19 CAR-T therapy for adults with heavily pretreated relapsed/refractory large B-cell lymphoma in China %J Cancer Med. 2021;10(3):999-1011.

13. MJ F, J D, K G, M R, JT J, D F. Safety and efficacy of tisagenlecleucel in primary CNS lymphoma: A phase 1/2 clinical trial %J Blood. 2022;139(15):2306-15.

14. G S, SJ S, M D, L F, J K, PEM P. Efficacy comparison of tisagenlecleucel vs usual care in patients with relapsed or refractory follicular lymphoma %J Blood Adv. 2022;6(22):5835-43.

15. H J, A K, D K, S R, N G, R P. High efficacy and excellent safety profile of actalycabtagene autoleucel, a humanized CD19 CAR-T product in r/r B-cell malignancies: A phase II pivotal trial %J Blood. 2023;142(Supplement 1):4838.

16. X Z, S T, C W, R H, L D, C S. Phase I trial of fourth-generation anti-CD19 chimeric antigen receptor T cells against relapsed or refractory B cell non-hodgkin lymphomas %J Front Immunol. 2020;11:564099.

17. C H, L W, R L, W L, Z L, J L. Efficacy and safety of CD19 chimeric antigen receptor T cells in the treatment of 11 patients with relapsed/refractory B-cell lymphoma: A single-center study %J Ann Transl Med. 2020;8(17):1048.

18. X C, X L, Y L, Z Z, X Z, J H. A phase I clinical trial of chimeric antigen receptor-modified T cells in patients with relapsed and refractory lymphoma %J Immunotherapy. 2020;12(10):681-96.

19. Z Y, XF H, X X, Y L, X K, Y S. A safe and potent anti-CD19 CAR T cell therapy %J Nat Med. 2019;25(6):947-53.

20. MJ D, P B, U J, NN S, D B, J B. A novel autologous CAR-T therapy, YTB323, with preserved T-cell stemness shows enhanced CAR T-cell efficacy in preclinical and early clinical development %J Cancer Discov. 2023;13(9):1982-97.

21. J M, CR B, MR B, PJ H, HS M, MJ D, et al. A phase 1 dose escalation and cohort expansion study of the safety and efficacy of allogeneic CRISPR-Cas9-engineered T cells (CTX110) in patients (Pts) with relapsed or refractory (R/R) B-cell malignancies (CARBON) %J J Clin Oncol2021; 39(15_suppl):[TPS7570-TPS pp.]. Available from: <https://ascopubs.org/doi/10.1200/JCO.2021.39.15_suppl.TPS7570>.

22. N J, H K, SR S, F H, CS S, CR H, et al. Preliminary Safety and Efficacy of PBCAR0191, an Allogeneic 'Off-the-Shelf' CD19-Directed CAR-T for Patients with Relapsed/Refractory (R/R) CD19+ B-ALL %J Blood. 2021;138(Supplement 1):650-.

23. Z Y, T H, X W, W Z, N L, M T. Distribution of chimeric antigen receptor-modified T cells against CD19 in B-cell malignancies %J BMC Cancer. 2021;21(1):198.

24. Y H, C Z, M Z, G W, W L, S F. Safety and efficacy of CRISPR-based non-viral PD1 locus specifically integrated anti-CD19 CAR-T cells in patients with relapsed or refractory non-hodgkin’s lymphoma: A first-in-human phase I study %J EClinicalMedicine. 2023;60:102010.

25. W L, A Z, H L, C Y, C W, S G. Safety and feasibility of anti-CD19 CAR T cells expressing inducible IL-7 and CCL19 in patients with relapsed or refractory large B-cell lymphoma %J Cell Discov. 2024;10(1):5.

26. J W, Y J, M L, W L, J H, M Z. Phase I clinical trial of CD19 CAR-T cells expressing CXCR5 protein for the treatment of relapsed or refractory B-cell lymphoma %J Curr Cancer Drug Targets. 2025;25(9):1170-9.

27. JH P, ML P, K P, SM D, G S, PB D. Results from first-in-human phase I study of a novel CD19-1XX chimeric antigen receptor with calibrated signaling in large B-cell lymphoma %J J Clin Oncol Off J Am Soc Clin Oncol. 2025;43(21):2418-28.

28. E E, K Y, C H, A S, U S, DD K. Preliminary report of the academic CAR-T (ISIKOK-19) cell clinical trial in turkey: Characterization of product and outcomes of clinical application %J Turk J Haematol Off J Turk Soc Haematol. 2022;39(3):206-10.

29. CS S, B S, I R, A N, Y B, X W. CD19 CAR T cells following autologous transplantation in poor-risk relapsed and refractory B-cell non-hodgkin lymphoma %J Blood. 2019;134(7):626-35.

30. G E, H K, G G, J W, T L, RM A. A phase I/IIa trial using CD19-targeted third-generation CAR T cells for lymphoma and leukemia %J Clin Cancer Res Off J Am Assoc Cancer Res. 2018;24(24):6185-94.

31. I D, G C, R S, MJ B, E J, A S. Treatment with anti CD19 chimeric antigen receptor T cells after antibody-based immunotherapy in adults with acute lymphoblastic leukemia %J Curr Res Transl Med. 2020;68(1):17-22.

32. W Y, P L, L Z, M Y, S Y, D Z. A phase 1 trial of prizloncabtagene autoleucel, a CD19/CD20 CAR T-cell therapy for relapsed/refractory B-cell non-hodgkin lymphoma %J Blood. 2025;145(14):1526-35.

33. W S, M S, J Y, J C, L X, D Y. Phase II trial of co-administration of CD19- and CD20-targeted chimeric antigen receptor T cells for relapsed and refractory diffuse large B cell lymphoma %J Cancer Med. 2020;9(16):5827-38.

34. JM K, A S, I A, R W, A E, E H. Patient-reported outcomes and neurotoxicity markers in patients treated with bispecific LV20.19 CAR T cell therapy %J Commun Med. 2022;2(1):49.

35. SM L, CM W, B J, SN G, J N, J T. CD19/CD20 bispecific chimeric antigen receptor (CAR) in naive/memory T cells for the treatment of relapsed or refractory non-hodgkin lymphoma %J Cancer Discov. 2023;13(3):580-97.

36. P B, A J, P G, H B-W, C S, FA A. Phase I trial of MB-CART2019.1, a novel CD20 and CD19 targeting tandem chimeric antigen receptor, in patients with relapsed or refractory B-cell non-hodgkin lymphoma %J Blood. 2020;136:48.

37. L W, C F, Q K, W H, Z C, W Z, et al. Bispecific CAR-T cells targeting CD19/20 in patients with relapsed or refractory B cell non-Hodgkin lymphoma: a phase I/II trial %J Blood Cancer J2024; 14(1). Available from: <https://www.nature.com/articles/s41408-024-01105-8>.

38. F H, Y L, Y Z, X M, C T, J W. Intrinsic NPRL2 and NPRL3 regulate the sensitivity of B-cell malignancies to CAR-T cell therapy %J J Genet Genomics Yi Chuan Xue Bao. 2025;S1673-8527(25).

39. J Z, Y Z, M S, X Z, H G, J L. Cytopenia after chimeric antigen receptor T cell immunotherapy in relapsed or refractory lymphoma %J Front Immunol. 2022;13:997589.

40. H S, C J, P S, N J, M H, E E. A phase 1 clinical trial of NKTR-255 with CD19-22 CAR T-cell therapy for refractory B-cell acute lymphoblastic leukemia %J Blood. 2024;144(16):1689-98.

41. C R, LJ L, MAV M, A R, Y Z, Y H. Dual targeting of CD19 and CD22 with bicistronic CAR-T cells in patients with relapsed/refractory large B-cell lymphoma %J Blood. 2023;141(20):2470-82.

42. N W, X H, W C, C L, Y X, Y C. Efficacy and safety of CAR19/22 T-cell cocktail therapy in patients with refractory/relapsed B-cell malignancies %J Blood. 2020;135(1):17-27.

43. Q C, J T, R L, L K, Y Z, E W. CD20-specific chimeric antigen receptor-expressing T cells as salvage therapy in rituximab-refractory/relapsed B-cell non-hodgkin lymphoma %J Cytotherapy. 2022;24(10):1026-34.

44. M S, AK G, SD S, RC L, CS U, CJ T. CD20 targeted CAR-T for high-risk B-cell non-hodgkin lymphomas %J Blood. 2019;134:3235.

45. J M, S J, MS M, I I, PJ S, J S. A phase 1 study of ACTR087 in combination with rituximab, in subjects with relapsed or refractory CD20-positive B-cell lymphoma %J Blood. 2019;134(Supplement_1):244.

46. WS K, TM K, SG C, I J, E I-J, LM P. Odronextamab monotherapy in patients with relapsed/refractory diffuse large B cell lymphoma: Primary efficacy and safety analysis in phase 2 ELM-2 trial %J Nat Cancer. 2025;6(3):528-39.

47. M H, R M, MR C, P J, KM L, MED C. Dose escalation of subcutaneous epcoritamab in patients with relapsed or refractory B-cell non-hodgkin lymphoma: An open-label, phase 1/2 study %J Lancet Lond Engl. 2021;398(10306):1157-69.

48. JD B, J J, D B, R C, M T, U V. Epcoritamab plus GemOx in transplant-ineligible relapsed/refractory DLBCL: Results from the EPCORE NHL-2 trial %J Blood. 2025;145(15):1621-31.

49. Y S, Z L, L L, Z Q, K Z, L F. GB261, an fc-function enabled and CD3 affinity de-tuned CD20/CD3 bispecific antibody, demonstrated a highly advantageous safety/efficacy balance in an ongoing first-in-human dose-escalation study in patients with relapsed/refractory non-hodgkin lymphoma %J Blood. 2023;142(Supplement 1):1719.

50. TJ P, C C-S, F M, E B, M C, M T. Glofitamab in relapsed/refractory mantle cell lymphoma: Results from a phase I/II study %J J Clin Oncol Off J Am Soc Clin Oncol. 2025;43(3):318-28.

51. LE B, LH S, M M, SJ S, S A, P G, et al. Safety and efficacy of mosunetuzumab, a bispecific antibody, in patients with relapsed or refractory follicular lymphoma: a single-arm, multicentre, phase 2 study %J Lancet Oncol2022; 23(8):[1055-65 pp.]. Available from: <https://pubmed.ncbi.nlm.nih.gov/35803286/>.

52. R B, JE A, RH A, JR B, JN A, SM A. Odronextamab, a human CD20×CD3 bispecific antibody in patients with CD20-positive B-cell malignancies (ELM-1): Results from the relapsed or refractory non-hodgkin lymphoma cohort in a single-arm, multicentre, phase 1 trial %J Lancet Haematol. 2022;9(5):e327-39.

53. V N, G Z, ME G, A V, R B, P K. Relationship of T- and B-cell kinetics to clinical response in patients with relapsed/refractory non-hodgkin lymphoma treated with blinatumomab %J Exp Hematol. 2021;100:32-6.

54. A G, NC F, J C, MP R, AF C, L G, et al. Blinatumomab consolidation post-autologous stem cell transplantation in patients with diffuse large B-cell lymphoma %J Blood Adv2024; 8(3):[513-22 pp.]. Available from: <https://pubmed.ncbi.nlm.nih.gov/37871306/>.

55. DA K, JD M, MP C, KA D, C T, NJ M. Open-label, phase 2 study of blinatumomab after frontline R-chemotherapy in adults with newly diagnosed, high-risk DLBCL %J Leuk Lymphoma. 2022;63(9):2063-73.

56. L C, NJ M, A R, KD M, G V, CL F, et al. Open-label, phase 2 study of blinatumomab as second salvage therapy in adults with relapsed/refractory aggressive B-cell non-Hodgkin lymphoma %J Leuk Lymphoma2020; 61(9):[2103-12 pp.]. Available from: <https://pubmed.ncbi.nlm.nih.gov/32546071/>.

57. A V, ME G, G H, S N, M P, N A. Phase 2 study of the bispecific T-cell engager (BiTE) antibody blinatumomab in relapsed/refractory diffuse large B-cell lymphoma %J Blood. 2016;127(11):1410-6.

58. WP L, B J, YR S, X Z, XF Z, Y T. Safety and efficacy of a-319 (a CD3xCD19 T cell engager) in patients with relapsed or refractory B-cell non-hodgkin lymphoma %J Blood. 2023;142:6158.

| **Comparison** | **Outcome** | **Event1** | **N1** | **Rate1** | **Event2** | **N2** | **Rate2** | **RD_CI** | **p_value** |
| --- | --- | --- | --- | --- | --- | --- | --- | --- | --- |
| Overall vs CD19 | ORR | 1332 | 1893 | 0.704 | 966 | 1442 | 0.67 | 0.034 (0.002, 0.066) | 0.037022493 |
| Overall vs CD19/20 | ORR | 1332 | 1893 | 0.704 | 174 | 211 | 0.825 | -0.121 (-0.176, -0.066) | 0.000218817 |
| Overall vs CD20 | ORR | 1332 | 1893 | 0.704 | 43 | 57 | 0.754 | -0.051 (-0.164, 0.063) | 0.407807526 |
| Overall vs CD19/22 | ORR | 1332 | 1893 | 0.704 | 149 | 183 | 0.814 | -0.111 (-0.171, -0.051) | 0.001585594 |
| CD19 vs CD19/20 | ORR | 966 | 1442 | 0.67 | 174 | 211 | 0.825 | -0.155 (-0.212, -0.098) | 5.68131E-06 |
| CD19 vs CD20 | ORR | 966 | 1442 | 0.67 | 43 | 57 | 0.754 | -0.084 (-0.199, 0.03) | 0.182313576 |
| CD19 vs CD19/22 | ORR | 966 | 1442 | 0.67 | 149 | 183 | 0.814 | -0.144 (-0.206, -0.083) | 7.4102E-05 |
| CD19/20 vs CD20 | ORR | 174 | 211 | 0.825 | 43 | 57 | 0.754 | 0.07 (-0.053, 0.193) | 0.230515802 |
| CD19/20 vs CD19/22 | ORR | 174 | 211 | 0.825 | 149 | 183 | 0.814 | 0.01 (-0.066, 0.087) | 0.788070883 |
| CD20 vs CD19/22 | ORR | 43 | 57 | 0.754 | 149 | 183 | 0.814 | -0.06 (-0.185, 0.065) | 0.324156409 |
| Overall vs CD19 | CR | 1041 | 1893 | 0.55 | 747 | 1442 | 0.518 | 0.032 (-0.002, 0.066) | 0.067323214 |
| Overall vs CD19/20 | CR | 1041 | 1893 | 0.55 | 146 | 211 | 0.692 | -0.142 (-0.208, -0.076) | 7.93801E-05 |
| Overall vs CD20 | CR | 1041 | 1893 | 0.55 | 29 | 57 | 0.509 | 0.041 (-0.091, 0.173) | 0.538480649 |
| Overall vs CD19/22 | CR | 1041 | 1893 | 0.55 | 119 | 183 | 0.65 | -0.1 (-0.173, -0.028) | 0.009034304 |
| CD19 vs CD19/20 | CR | 747 | 1442 | 0.518 | 146 | 211 | 0.692 | -0.174 (-0.241, -0.106) | 2.19776E-06 |
| CD19 vs CD20 | CR | 747 | 1442 | 0.518 | 29 | 57 | 0.509 | 0.009 (-0.123, 0.142) | 0.890869891 |
| CD19 vs CD19/22 | CR | 747 | 1442 | 0.518 | 119 | 183 | 0.65 | -0.132 (-0.206, -0.058) | 0.00073083 |
| CD19/20 vs CD20 | CR | 146 | 211 | 0.692 | 29 | 57 | 0.509 | 0.183 (0.039, 0.327) | 0.00994426 |
| CD19/20 vs CD19/22 | CR | 146 | 211 | 0.692 | 119 | 183 | 0.65 | 0.042 (-0.051, 0.135) | 0.379367568 |
| CD20 vs CD19/22 | CR | 29 | 57 | 0.509 | 119 | 183 | 0.65 | -0.142 (-0.289, 0.006) | 0.055023901 |
| Overall vs CD19 | CRS | 138 | 1893 | 0.073 | 84 | 1442 | 0.058 | 0.015 (-0.002, 0.031) | 0.092730995 |
| Overall vs CD19/20 | CRS | 138 | 1893 | 0.073 | 17 | 211 | 0.081 | -0.008 (-0.046, 0.031) | 0.685870389 |
| Overall vs CD20 | CRS | 138 | 1893 | 0.073 | 9 | 57 | 0.158 | -0.085 (-0.18, 0.01) | 0.016630563 |
| Overall vs CD19/22 | CRS | 138 | 1893 | 0.073 | 28 | 183 | 0.153 | -0.08 (-0.134, -0.027) | 0.000136132 |
| CD19 vs CD19/20 | CRS | 84 | 1442 | 0.058 | 17 | 211 | 0.081 | -0.022 (-0.061, 0.016) | 0.206200021 |
| CD19 vs CD20 | CRS | 84 | 1442 | 0.058 | 9 | 57 | 0.158 | -0.1 (-0.195, -0.004) | 0.002223329 |
| CD19 vs CD19/22 | CRS | 84 | 1442 | 0.058 | 28 | 183 | 0.153 | -0.095 (-0.148, -0.041) | 1.87454E-06 |
| CD19/20 vs CD20 | CRS | 17 | 211 | 0.081 | 9 | 57 | 0.158 | -0.077 (-0.179, 0.024) | 0.080091001 |
| CD19/20 vs CD19/22 | CRS | 17 | 211 | 0.081 | 28 | 183 | 0.153 | -0.072 (-0.136, -0.009) | 0.024163232 |
| CD20 vs CD19/22 | CRS | 9 | 57 | 0.158 | 28 | 183 | 0.153 | 0.005 (-0.103, 0.113) | 0.928874129 |
| Overall vs CD19 | Neurotoxicity≥ Grade 3 | 162 | 1893 | 0.086 | 132 | 1442 | 0.092 | -0.006 (-0.025, 0.014) | 0.547494644 |
| Overall vs CD19/20 | Neurotoxicity≥ Grade 3 | 162 | 1893 | 0.086 | 7 | 211 | 0.033 | 0.052 (0.025, 0.08) | 0.007895265 |
| Overall vs CD20 | Neurotoxicity≥ Grade 3 | 162 | 1893 | 0.086 | 3 | 57 | 0.053 | 0.033 (-0.026, 0.092) | 0.378529514 |
| Overall vs CD19/22 | Neurotoxicity≥ Grade 3 | 162 | 1893 | 0.086 | 20 | 183 | 0.109 | -0.024 (-0.071, 0.023) | 0.27879361 |
| CD19 vs CD19/20 | Neurotoxicity≥ Grade 3 | 132 | 1442 | 0.092 | 7 | 211 | 0.033 | 0.058 (0.03, 0.087) | 0.004327773 |
| CD19 vs CD20 | Neurotoxicity≥ Grade 3 | 132 | 1442 | 0.092 | 3 | 57 | 0.053 | 0.039 (-0.021, 0.099) | 0.314207014 |
| CD19 vs CD19/22 | Neurotoxicity≥ Grade 3 | 132 | 1442 | 0.092 | 20 | 183 | 0.109 | -0.018 (-0.065, 0.03) | 0.4372736 |
| CD19/20 vs CD20 | Neurotoxicity≥ Grade 3 | 7 | 211 | 0.033 | 3 | 57 | 0.053 | -0.019 (-0.082, 0.043) | 0.491645987 |
| CD19/20 vs CD19/22 | Neurotoxicity≥ Grade 3 | 7 | 211 | 0.033 | 20 | 183 | 0.109 | -0.076 (-0.127, -0.025) | 0.002859973 |
| CD20 vs CD19/22 | Neurotoxicity≥ Grade 3 | 3 | 57 | 0.053 | 20 | 183 | 0.109 | -0.057 (-0.13, 0.017) | 0.204467408 |
| Overall vs CD19 | Infection≥ Grade 3 | 164 | 1893 | 0.087 | 153 | 1442 | 0.106 | -0.019 (-0.04, 0.001) | 0.057561319 |
| Overall vs CD19/20 | Infection≥ Grade 3 | 164 | 1893 | 0.087 | 10 | 211 | 0.047 | 0.039 (0.008, 0.071) | 0.049639558 |
| CD19 vs CD19/20 | Infection≥ Grade 3 | 153 | 1442 | 0.106 | 10 | 211 | 0.047 | 0.059 (0.026, 0.091) | 0.007547868 |
| Overall vs CD19 | ICANS≥ Grade 3 | 262 | 1893 | 0.138 | 214 | 1442 | 0.148 | -0.01 (-0.034, 0.014) | 0.413403483 |
| Overall vs CD19/20 | ICANS≥ Grade 3 | 262 | 1893 | 0.138 | 24 | 211 | 0.114 | 0.025 (-0.021, 0.07) | 0.321475043 |
| Overall vs CD20 | ICANS≥ Grade 3 | 262 | 1893 | 0.138 | 4 | 57 | 0.07 | 0.068 (0, 0.136) | 0.13921282 |
| Overall vs CD19/22 | ICANS≥ Grade 3 | 262 | 1893 | 0.138 | 20 | 183 | 0.109 | 0.029 (-0.019, 0.077) | 0.272321876 |
| CD19 vs CD19/20 | ICANS≥ Grade 3 | 214 | 1442 | 0.148 | 24 | 211 | 0.114 | 0.035 (-0.012, 0.081) | 0.180417066 |
| CD19 vs CD20 | ICANS≥ Grade 3 | 214 | 1442 | 0.148 | 4 | 57 | 0.07 | 0.078 (0.009, 0.147) | 0.100342739 |
| CD19 vs CD19/22 | ICANS≥ Grade 3 | 214 | 1442 | 0.148 | 20 | 183 | 0.109 | 0.039 (-0.01, 0.088) | 0.155681436 |
| CD19/20 vs CD20 | ICANS≥ Grade 3 | 24 | 211 | 0.114 | 4 | 57 | 0.07 | 0.044 (-0.035, 0.123) | 0.339986885 |
| CD19/20 vs CD19/22 | ICANS≥ Grade 3 | 24 | 211 | 0.114 | 20 | 183 | 0.109 | 0.004 (-0.058, 0.067) | 0.888654542 |
| CD20 vs CD19/22 | ICANS≥ Grade 3 | 4 | 57 | 0.07 | 20 | 183 | 0.109 | -0.039 (-0.119, 0.041) | 0.390038053 |
| Overall vs CD3xCD20 | ORR | 537 | 1021 | 0.526 | 456 | 835 | 0.546 | -0.02 (-0.066, 0.025) | 0.386508313 |
| Overall vs CD3xCD19 | ORR | 537 | 1021 | 0.526 | 81 | 186 | 0.435 | 0.09 (0.013, 0.168) | 0.023190149 |
| CD3xCD20 vs CD3xCD19 | ORR | 456 | 835 | 0.546 | 81 | 186 | 0.435 | 0.111 (0.032, 0.189) | 0.006286575 |
| Overall vs CD3xCD20 | CR | 367 | 1021 | 0.359 | 310 | 835 | 0.371 | -0.012 (-0.056, 0.032) | 0.599132461 |
| Overall vs CD3xCD19 | CR | 367 | 1021 | 0.359 | 57 | 186 | 0.306 | 0.053 (-0.019, 0.125) | 0.163733147 |
| CD3xCD20 vs CD3xCD19 | CR | 310 | 835 | 0.371 | 57 | 186 | 0.306 | 0.065 (-0.009, 0.139) | 0.095766878 |
| Overall vs CD3xCD20 | CRS | 32 | 1021 | 0.031 | 31 | 835 | 0.037 | -0.006 (-0.022, 0.011) | 0.493625654 |
| Overall vs CD3xCD19 | CRS | 32 | 1021 | 0.031 | 1 | 186 | 0.005 | 0.026 (0.011, 0.041) | 0.045800106 |
| CD3xCD20 vs CD3xCD19 | CRS | 31 | 835 | 0.037 | 1 | 186 | 0.005 | 0.032 (0.015, 0.048) | 0.024616242 |
| Overall vs CD3xCD20 | Neurotoxicity≥ Grade 3 | 47 | 1021 | 0.046 | 14 | 835 | 0.017 | 0.029 (0.014, 0.045) | 0.000434443 |
| Overall vs CD3xCD19 | Neurotoxicity≥ Grade 3 | 47 | 1021 | 0.046 | 33 | 186 | 0.177 | -0.131 (-0.188, -0.075) | 3.47987E-11 |
| CD3xCD20 vs CD3xCD19 | Neurotoxicity≥ Grade 3 | 14 | 835 | 0.017 | 33 | 186 | 0.177 | -0.161 (-0.216, -0.105) | 3.22272E-21 |
| Overall vs CD3xCD20 | Infection≥ Grade 3 | 165 | 1021 | 0.162 | 152 | 835 | 0.182 | -0.02 (-0.055, 0.014) | 0.244636583 |
| Overall vs CD3xCD19 | Infection≥ Grade 3 | 165 | 1021 | 0.162 | 13 | 186 | 0.07 | 0.092 (0.049, 0.135) | 0.001176812 |
| CD3xCD20 vs CD3xCD19 | Infection≥ Grade 3 | 152 | 835 | 0.182 | 13 | 186 | 0.07 | 0.112 (0.067, 0.157) | 0.000171568 |
| Overall vs CD3xCD20 | ICANS≥ Grade 3 | 47 | 1021 | 0.046 | 19 | 835 | 0.023 | 0.023 (0.007, 0.04) | 0.00705873 |
| Overall vs CD3xCD19 | ICANS≥ Grade 3 | 47 | 1021 | 0.046 | 28 | 186 | 0.151 | -0.105 (-0.157, -0.052) | 5.63398E-08 |
| CD3xCD20 vs CD3xCD19 | ICANS≥ Grade 3 | 19 | 835 | 0.023 | 28 | 186 | 0.151 | -0.128 (-0.18, -0.075) | 5.44875E-14 |
| CAR-T_Overall vs BsAbs_Overall | ORR | 1332 | 1893 | 0.704 | 537 | 1021 | 0.526 | 0.178 (0.141, 0.215) | 1.40286E-21 |
| CAR-T_Overall vs BsAbs_CD3xCD20 | ORR | 1332 | 1893 | 0.704 | 456 | 835 | 0.546 | 0.158 (0.118, 0.197) | 1.46915E-15 |
| CAR-T_Overall vs BsAbs_CD3xCD19 | ORR | 1332 | 1893 | 0.704 | 81 | 186 | 0.435 | 0.268 (0.194, 0.342) | 7.48595E-14 |
| CAR-T_CD19 vs BsAbs_Overall | ORR | 966 | 1442 | 0.67 | 537 | 1021 | 0.526 | 0.144 (0.105, 0.183) | 5.33979E-13 |
| CAR-T_CD19 vs BsAbs_CD3xCD20 | ORR | 966 | 1442 | 0.67 | 456 | 835 | 0.546 | 0.124 (0.082, 0.165) | 4.1361E-09 |
| CAR-T_CD19 vs BsAbs_CD3xCD19 | ORR | 966 | 1442 | 0.67 | 81 | 186 | 0.435 | 0.234 (0.159, 0.31) | 3.37374E-10 |
| CAR-T_CD19/20 vs BsAbs_Overall | ORR | 174 | 211 | 0.825 | 537 | 1021 | 0.526 | 0.299 (0.239, 0.358) | 1.2943E-15 |
| CAR-T_CD19/20 vs BsAbs_CD3xCD20 | ORR | 174 | 211 | 0.825 | 456 | 835 | 0.546 | 0.279 (0.217, 0.34) | 1.51184E-13 |
| CAR-T_CD19/20 vs BsAbs_CD3xCD19 | ORR | 174 | 211 | 0.825 | 81 | 186 | 0.435 | 0.389 (0.301, 0.477) | 6.88775E-16 |
| CAR-T_CD20 vs BsAbs_Overall | ORR | 43 | 57 | 0.754 | 537 | 1021 | 0.526 | 0.228 (0.113, 0.344) | 0.000761126 |
| CAR-T_CD20 vs BsAbs_CD3xCD20 | ORR | 43 | 57 | 0.754 | 456 | 835 | 0.546 | 0.208 (0.092, 0.325) | 0.002180306 |
| CAR-T_CD20 vs BsAbs_CD3xCD19 | ORR | 43 | 57 | 0.754 | 81 | 186 | 0.435 | 0.319 (0.186, 0.451) | 2.51151E-05 |
| CAR-T_CD19/22 vs BsAbs_Overall | ORR | 149 | 183 | 0.814 | 537 | 1021 | 0.526 | 0.288 (0.224, 0.352) | 4.08625E-13 |
| CAR-T_CD19/22 vs BsAbs_CD3xCD20 | ORR | 149 | 183 | 0.814 | 456 | 835 | 0.546 | 0.268 (0.202, 0.334) | 2.24102E-11 |
| CAR-T_CD19/22 vs BsAbs_CD3xCD19 | ORR | 149 | 183 | 0.814 | 81 | 186 | 0.435 | 0.379 (0.288, 0.47) | 6.06573E-14 |
| CAR-T_Overall vs BsAbs_Overall | CR | 1041 | 1893 | 0.55 | 367 | 1021 | 0.359 | 0.19 (0.153, 0.227) | 9.58774E-23 |
| CAR-T_Overall vs BsAbs_CD3xCD20 | CR | 1041 | 1893 | 0.55 | 310 | 835 | 0.371 | 0.179 (0.139, 0.218) | 7.85834E-18 |
| CAR-T_Overall vs BsAbs_CD3xCD19 | CR | 1041 | 1893 | 0.55 | 57 | 186 | 0.306 | 0.243 (0.174, 0.313) | 2.19579E-10 |
| CAR-T_CD19 vs BsAbs_Overall | CR | 747 | 1442 | 0.518 | 367 | 1021 | 0.359 | 0.159 (0.119, 0.198) | 6.71297E-15 |
| CAR-T_CD19 vs BsAbs_CD3xCD20 | CR | 747 | 1442 | 0.518 | 310 | 835 | 0.371 | 0.147 (0.105, 0.188) | 1.30914E-11 |
| CAR-T_CD19 vs BsAbs_CD3xCD19 | CR | 747 | 1442 | 0.518 | 57 | 186 | 0.306 | 0.212 (0.14, 0.283) | 5.57757E-08 |
| CAR-T_CD19/20 vs BsAbs_Overall | CR | 146 | 211 | 0.692 | 367 | 1021 | 0.359 | 0.332 (0.264, 0.401) | 4.70443E-19 |
| CAR-T_CD19/20 vs BsAbs_CD3xCD20 | CR | 146 | 211 | 0.692 | 310 | 835 | 0.371 | 0.321 (0.25, 0.391) | 4.73566E-17 |
| CAR-T_CD19/20 vs BsAbs_CD3xCD19 | CR | 146 | 211 | 0.692 | 57 | 186 | 0.306 | 0.385 (0.295, 0.476) | 1.75258E-14 |
| CAR-T_CD20 vs BsAbs_Overall | CR | 29 | 57 | 0.509 | 367 | 1021 | 0.359 | 0.149 (0.016, 0.282) | 0.022856419 |
| CAR-T_CD20 vs BsAbs_CD3xCD20 | CR | 29 | 57 | 0.509 | 310 | 835 | 0.371 | 0.138 (0.004, 0.271) | 0.038506532 |
| CAR-T_CD20 vs BsAbs_CD3xCD19 | CR | 29 | 57 | 0.509 | 57 | 186 | 0.306 | 0.202 (0.057, 0.348) | 0.005194553 |
| CAR-T_CD19/22 vs BsAbs_Overall | CR | 119 | 183 | 0.65 | 367 | 1021 | 0.359 | 0.291 (0.216, 0.366) | 1.53477E-13 |
| CAR-T_CD19/22 vs BsAbs_CD3xCD20 | CR | 119 | 183 | 0.65 | 310 | 835 | 0.371 | 0.279 (0.203, 0.355) | 4.42639E-12 |
| CAR-T_CD19/22 vs BsAbs_CD3xCD19 | CR | 119 | 183 | 0.65 | 57 | 186 | 0.306 | 0.344 (0.248, 0.44) | 3.80704E-11 |
| CAR-T_Overall vs BsAbs_Overall | CRS | 138 | 1893 | 0.073 | 32 | 1021 | 0.031 | 0.042 (0.026, 0.057) | 4.96156E-06 |
| CAR-T_Overall vs BsAbs_CD3xCD20 | CRS | 138 | 1893 | 0.073 | 31 | 835 | 0.037 | 0.036 (0.018, 0.053) | 0.000354007 |
| CAR-T_Overall vs BsAbs_CD3xCD19 | CRS | 138 | 1893 | 0.073 | 1 | 186 | 0.005 | 0.068 (0.052, 0.083) | 0.000434655 |
| CAR-T_CD19 vs BsAbs_Overall | CRS | 84 | 1442 | 0.058 | 32 | 1021 | 0.031 | 0.027 (0.011, 0.043) | 0.00189801 |
| CAR-T_CD19 vs BsAbs_CD3xCD20 | CRS | 84 | 1442 | 0.058 | 31 | 835 | 0.037 | 0.021 (0.004, 0.039) | 0.02651992 |
| CAR-T_CD19 vs BsAbs_CD3xCD19 | CRS | 84 | 1442 | 0.058 | 1 | 186 | 0.005 | 0.053 (0.037, 0.069) | 0.002281289 |
| CAR-T_CD19/20 vs BsAbs_Overall | CRS | 17 | 211 | 0.081 | 32 | 1021 | 0.031 | 0.049 (0.011, 0.087) | 0.00086542 |
| CAR-T_CD19/20 vs BsAbs_CD3xCD20 | CRS | 17 | 211 | 0.081 | 31 | 835 | 0.037 | 0.043 (0.005, 0.082) | 0.007048677 |
| CAR-T_CD19/20 vs BsAbs_CD3xCD19 | CRS | 17 | 211 | 0.081 | 1 | 186 | 0.005 | 0.075 (0.037, 0.113) | 0.000326336 |
| CAR-T_CD20 vs BsAbs_Overall | CRS | 9 | 57 | 0.158 | 32 | 1021 | 0.031 | 0.127 (0.031, 0.222) | 1.1663E-06 |
| CAR-T_CD20 vs BsAbs_CD3xCD20 | CRS | 9 | 57 | 0.158 | 31 | 835 | 0.037 | 0.121 (0.025, 0.216) | 2.02114E-05 |
| CAR-T_CD20 vs BsAbs_CD3xCD19 | CRS | 9 | 57 | 0.158 | 1 | 186 | 0.005 | 0.153 (0.057, 0.248) | 3.94574E-07 |
| CAR-T_CD19/22 vs BsAbs_Overall | CRS | 28 | 183 | 0.153 | 32 | 1021 | 0.031 | 0.122 (0.068, 0.175) | 3.28302E-12 |
| CAR-T_CD19/22 vs BsAbs_CD3xCD20 | CRS | 28 | 183 | 0.153 | 31 | 835 | 0.037 | 0.116 (0.062, 0.17) | 1.23249E-09 |
| CAR-T_CD19/22 vs BsAbs_CD3xCD19 | CRS | 28 | 183 | 0.153 | 1 | 186 | 0.005 | 0.148 (0.094, 0.201) | 1.37164E-07 |
| CAR-T_Overall vs BsAbs_Overall | Neurotoxicity≥ Grade 3 | 162 | 1893 | 0.086 | 47 | 1021 | 0.046 | 0.04 (0.022, 0.058) | 7.91299E-05 |
| CAR-T_Overall vs BsAbs_CD3xCD20 | Neurotoxicity≥ Grade 3 | 162 | 1893 | 0.086 | 14 | 835 | 0.017 | 0.069 (0.053, 0.084) | 1.55898E-11 |
| CAR-T_Overall vs BsAbs_CD3xCD19 | Neurotoxicity≥ Grade 3 | 162 | 1893 | 0.086 | 33 | 186 | 0.177 | -0.092 (-0.148, -0.036) | 4.13933E-05 |
| CAR-T_CD19 vs BsAbs_Overall | Neurotoxicity≥ Grade 3 | 132 | 1442 | 0.092 | 47 | 1021 | 0.046 | 0.046 (0.026, 0.065) | 1.82147E-05 |
| CAR-T_CD19 vs BsAbs_CD3xCD20 | Neurotoxicity≥ Grade 3 | 132 | 1442 | 0.092 | 14 | 835 | 0.017 | 0.075 (0.058, 0.092) | 2.23225E-12 |
| CAR-T_CD19 vs BsAbs_CD3xCD19 | Neurotoxicity≥ Grade 3 | 132 | 1442 | 0.092 | 33 | 186 | 0.177 | -0.086 (-0.143, -0.029) | 0.000259673 |
| CAR-T_CD19/20 vs BsAbs_Overall | Neurotoxicity≥ Grade 3 | 7 | 211 | 0.033 | 47 | 1021 | 0.046 | -0.013 (-0.04, 0.015) | 0.40623252 |
| CAR-T_CD19/20 vs BsAbs_CD3xCD20 | Neurotoxicity≥ Grade 3 | 7 | 211 | 0.033 | 14 | 835 | 0.017 | 0.016 (-0.009, 0.042) | 0.128938527 |
| CAR-T_CD19/20 vs BsAbs_CD3xCD19 | Neurotoxicity≥ Grade 3 | 7 | 211 | 0.033 | 33 | 186 | 0.177 | -0.144 (-0.204, -0.084) | 1.89227E-06 |
| CAR-T_CD20 vs BsAbs_Overall | Neurotoxicity≥ Grade 3 | 3 | 57 | 0.053 | 47 | 1021 | 0.046 | 0.007 (-0.053, 0.066) | 0.817687668 |
| CAR-T_CD20 vs BsAbs_CD3xCD20 | Neurotoxicity≥ Grade 3 | 3 | 57 | 0.053 | 14 | 835 | 0.017 | 0.036 (-0.023, 0.094) | 0.055358756 |
| CAR-T_CD20 vs BsAbs_CD3xCD19 | Neurotoxicity≥ Grade 3 | 3 | 57 | 0.053 | 33 | 186 | 0.177 | -0.125 (-0.205, -0.045) | 0.020327971 |
| CAR-T_CD19/22 vs BsAbs_Overall | Neurotoxicity≥ Grade 3 | 20 | 183 | 0.109 | 47 | 1021 | 0.046 | 0.063 (0.016, 0.11) | 0.000587193 |
| CAR-T_CD19/22 vs BsAbs_CD3xCD20 | Neurotoxicity≥ Grade 3 | 20 | 183 | 0.109 | 14 | 835 | 0.017 | 0.093 (0.046, 0.139) | 2.80961E-10 |
| CAR-T_CD19/22 vs BsAbs_CD3xCD19 | Neurotoxicity≥ Grade 3 | 20 | 183 | 0.109 | 33 | 186 | 0.177 | -0.068 (-0.139, 0.003) | 0.062078162 |
| CAR-T_Overall vs BsAbs_Overall | Infection≥ Grade 3 | 164 | 1893 | 0.087 | 165 | 1021 | 0.162 | -0.075 (-0.101, -0.049) | 1.05419E-09 |
| CAR-T_Overall vs BsAbs_CD3xCD20 | Infection≥ Grade 3 | 164 | 1893 | 0.087 | 152 | 835 | 0.182 | -0.095 (-0.124, -0.066) | 7.19643E-13 |
| CAR-T_Overall vs BsAbs_CD3xCD19 | Infection≥ Grade 3 | 164 | 1893 | 0.087 | 13 | 186 | 0.07 | 0.017 (-0.022, 0.056) | 0.43497557 |
| CAR-T_CD19 vs BsAbs_Overall | Infection≥ Grade 3 | 153 | 1442 | 0.106 | 165 | 1021 | 0.162 | -0.056 (-0.083, -0.028) | 5.19016E-05 |
| CAR-T_CD19 vs BsAbs_CD3xCD20 | Infection≥ Grade 3 | 153 | 1442 | 0.106 | 152 | 835 | 0.182 | -0.076 (-0.107, -0.045) | 2.94896E-07 |
| CAR-T_CD19 vs BsAbs_CD3xCD19 | Infection≥ Grade 3 | 153 | 1442 | 0.106 | 13 | 186 | 0.07 | 0.036 (-0.004, 0.076) | 0.124557616 |
| CAR-T_CD19/20 vs BsAbs_Overall | Infection≥ Grade 3 | 10 | 211 | 0.047 | 165 | 1021 | 0.162 | -0.114 (-0.151, -0.078) | 1.51625E-05 |
| CAR-T_CD19/20 vs BsAbs_CD3xCD20 | Infection≥ Grade 3 | 10 | 211 | 0.047 | 152 | 835 | 0.182 | -0.135 (-0.173, -0.096) | 1.36519E-06 |
| CAR-T_CD19/20 vs BsAbs_CD3xCD19 | Infection≥ Grade 3 | 10 | 211 | 0.047 | 13 | 186 | 0.07 | -0.022 (-0.069, 0.024) | 0.338292973 |
| CAR-T_Overall vs BsAbs_Overall | ICANS≥ Grade 3 | 262 | 1893 | 0.138 | 47 | 1021 | 0.046 | 0.092 (0.072, 0.113) | 1.10477E-14 |
| CAR-T_Overall vs BsAbs_CD3xCD20 | ICANS≥ Grade 3 | 262 | 1893 | 0.138 | 19 | 835 | 0.023 | 0.116 (0.097, 0.134) | 5.27012E-20 |
| CAR-T_Overall vs BsAbs_CD3xCD19 | ICANS≥ Grade 3 | 262 | 1893 | 0.138 | 28 | 186 | 0.151 | -0.012 (-0.066, 0.042) | 0.648572885 |
| CAR-T_CD19 vs BsAbs_Overall | ICANS≥ Grade 3 | 214 | 1442 | 0.148 | 47 | 1021 | 0.046 | 0.102 (0.08, 0.125) | 4.23486E-16 |
| CAR-T_CD19 vs BsAbs_CD3xCD20 | ICANS≥ Grade 3 | 214 | 1442 | 0.148 | 19 | 835 | 0.023 | 0.126 (0.105, 0.147) | 1.52041E-21 |
| CAR-T_CD19 vs BsAbs_CD3xCD19 | ICANS≥ Grade 3 | 214 | 1442 | 0.148 | 28 | 186 | 0.151 | -0.002 (-0.057, 0.052) | 0.938665266 |
| CAR-T_CD19/20 vs BsAbs_Overall | ICANS≥ Grade 3 | 24 | 211 | 0.114 | 47 | 1021 | 0.046 | 0.068 (0.023, 0.112) | 0.000121972 |
| CAR-T_CD19/20 vs BsAbs_CD3xCD20 | ICANS≥ Grade 3 | 24 | 211 | 0.114 | 19 | 835 | 0.023 | 0.091 (0.047, 0.135) | 2.71749E-09 |
| CAR-T_CD19/20 vs BsAbs_CD3xCD19 | ICANS≥ Grade 3 | 24 | 211 | 0.114 | 28 | 186 | 0.151 | -0.037 (-0.104, 0.03) | 0.278227302 |
| CAR-T_CD20 vs BsAbs_Overall | ICANS≥ Grade 3 | 4 | 57 | 0.07 | 47 | 1021 | 0.046 | 0.024 (-0.043, 0.092) | 0.403416063 |
| CAR-T_CD20 vs BsAbs_CD3xCD20 | ICANS≥ Grade 3 | 4 | 57 | 0.07 | 19 | 835 | 0.023 | 0.047 (-0.02, 0.115) | 0.028848998 |
| CAR-T_CD20 vs BsAbs_CD3xCD19 | ICANS≥ Grade 3 | 4 | 57 | 0.07 | 28 | 186 | 0.151 | -0.08 (-0.164, 0.004) | 0.116471498 |
| CAR-T_CD19/22 vs BsAbs_Overall | ICANS≥ Grade 3 | 20 | 183 | 0.109 | 47 | 1021 | 0.046 | 0.063 (0.016, 0.11) | 0.000587193 |
| CAR-T_CD19/22 vs BsAbs_CD3xCD20 | ICANS≥ Grade 3 | 20 | 183 | 0.109 | 19 | 835 | 0.023 | 0.087 (0.04, 0.133) | 3.32382E-08 |
| CAR-T_CD19/22 vs BsAbs_CD3xCD19 | ICANS≥ Grade 3 | 20 | 183 | 0.109 | 28 | 186 | 0.151 | -0.041 (-0.11, 0.027) | 0.23892654 |

**Table S5. Summary of pairwise comparisons for key clinical outcomes across CAR-T and bispecific antibody (BsAbs) subgroups.**

For each comparison, the following data are presented:

Comparison: The two groups being compared (e.g., CAR-T_Overall vs BsAbs_Overall).

Outcome: The clinical outcome evaluated, including overall response rate (ORR), complete response (CR), cytokine release syndrome (CRS), neurotoxicity ≥ grade 3, infection ≥ grade 3, and immune effector cell-associated neurotoxicity syndrome (ICANS) ≥ grade 3.

Event1 / N1 / Rate1: Number of patients with the event, total sample size, and corresponding incidence rate in the first group.

Event2 / N2 / Rate2: Number of patients with the event, total sample size, and corresponding incidence rate in the second group.

p_value: Two-sided p-value from chi-square test (without continuity correction) comparing incidence rates between the two groups. A p-value < 0.05 was considered statistically significant.

RD (95% CI): Risk difference (RD) calculated as Rate1-Rate2, with its 95% confidence interval derived from the Wald method. A positive RD indicates a higher incidence in the first group, whereas a negative RD indicates a higher incidence in the second group. The 95% CI excluding zero corresponds to statistical significance at the 0.05 level.

*Abbreviations:* CAR-T, chimeric antigen receptor T-cell; BsAbs, bispecific antibodies; ORR, overall response rate; CR, complete response; CRS, cytokine release syndrome; ICANS, immune effector cell-associated neurotoxicity syndrome; CI, confidence interval; RD, risk difference.

*Note:* Comparisons with missing data (e.g., infection ≥ grade 3 in CAR-T CD20 and CD19/22 subgroups) were excluded from the analysis.


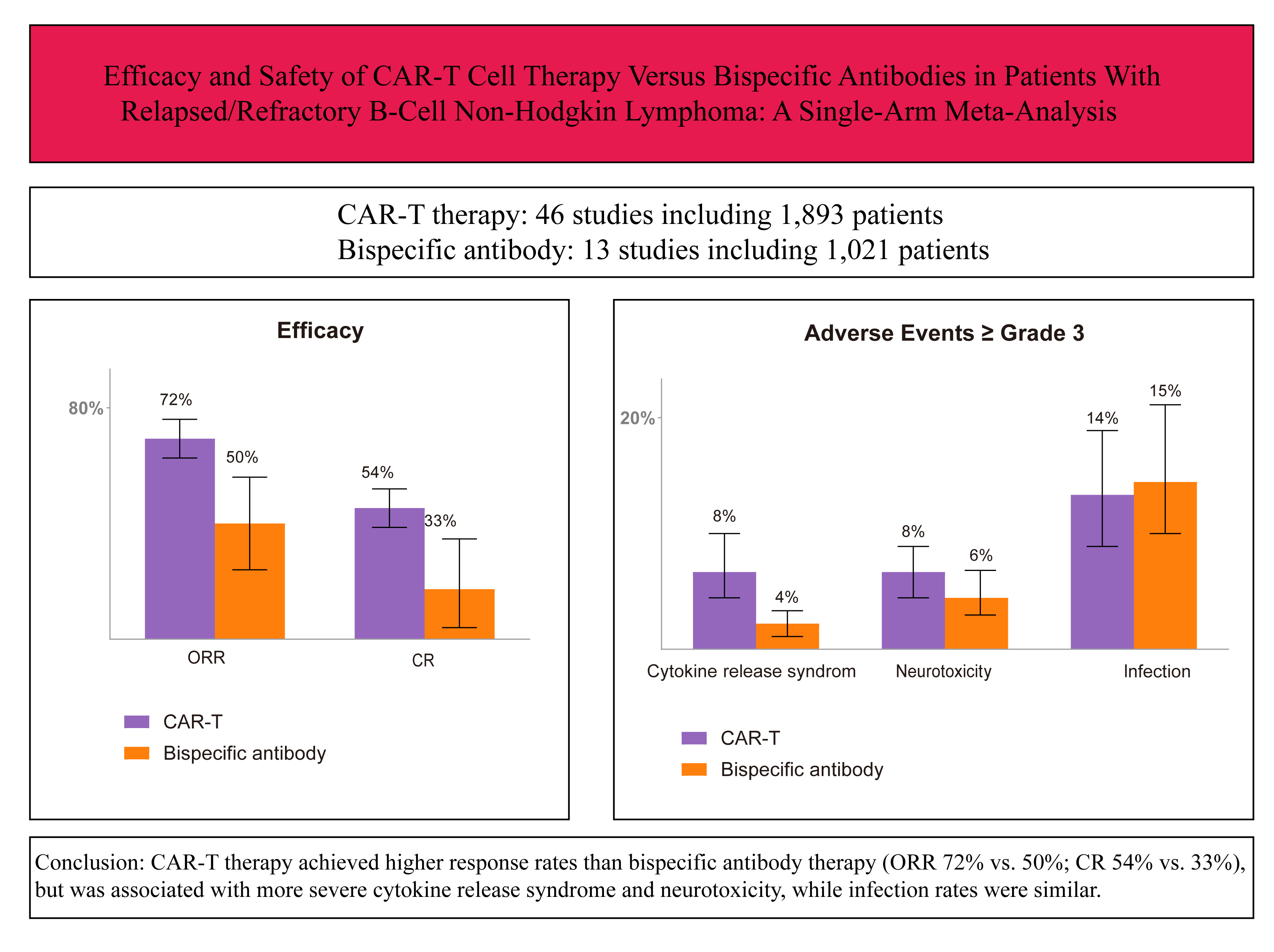


**Figure S1.** Graphical abstract


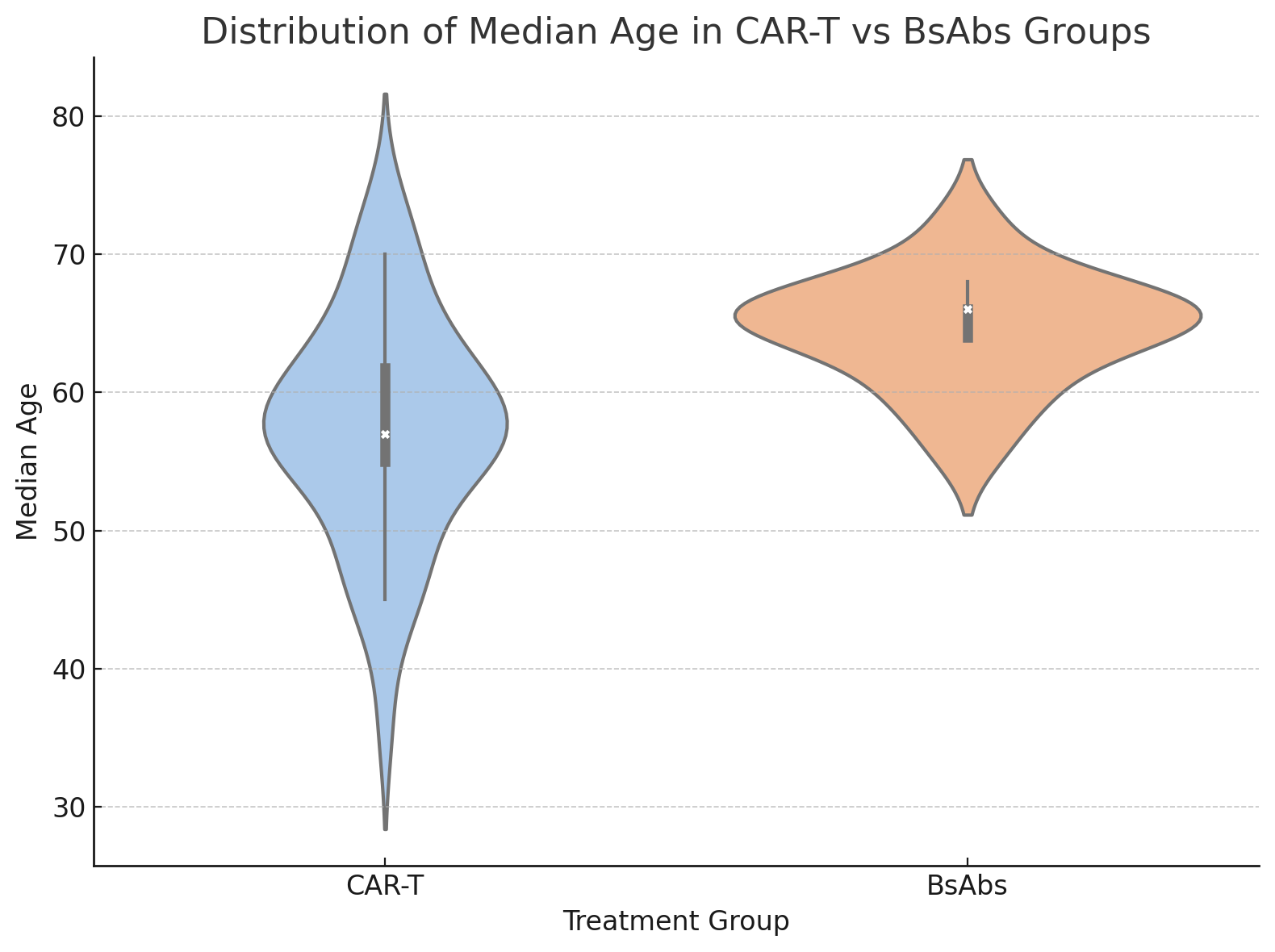


**Figure S2 Distribution of Median Age in CAR-T vs BsAbs Groups**

**
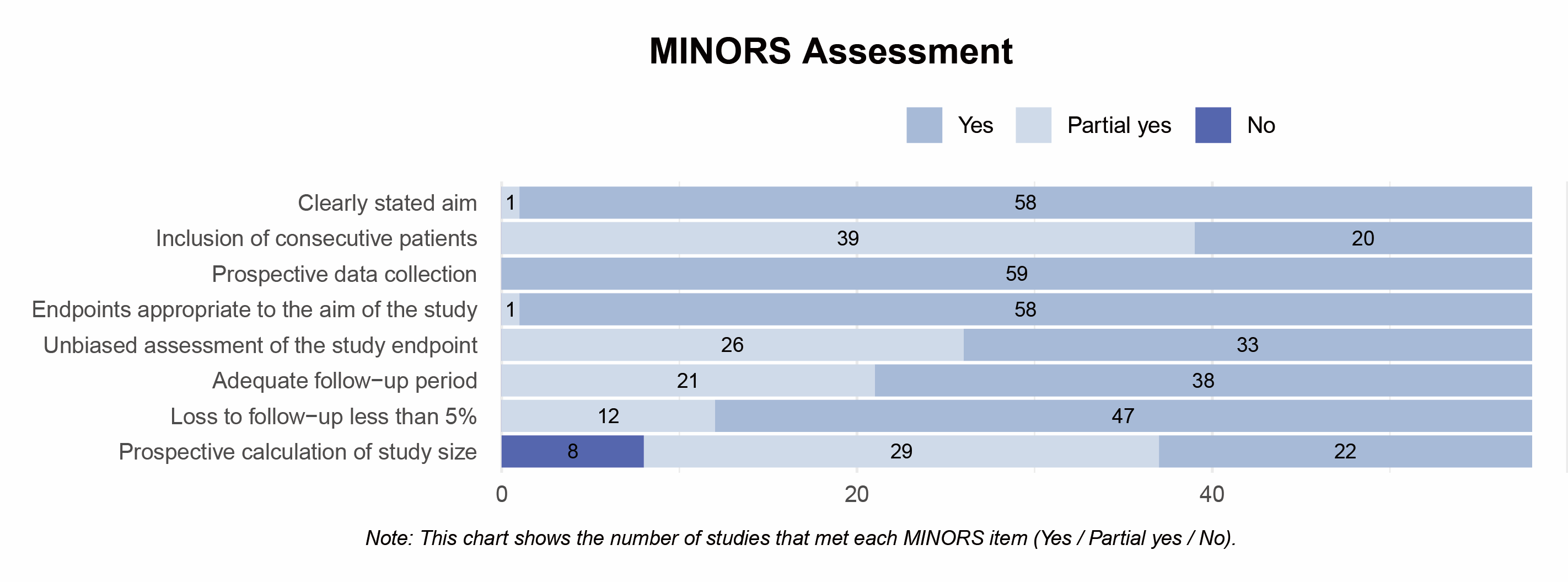
**

**Figure S3 Distribution of Median Age in CAR-T vs BsAbs Groups**

**Figure S4 CAT-T Egger Regression and sensitivity analysis**

Figure S4.1 Overall CAR-T ORR


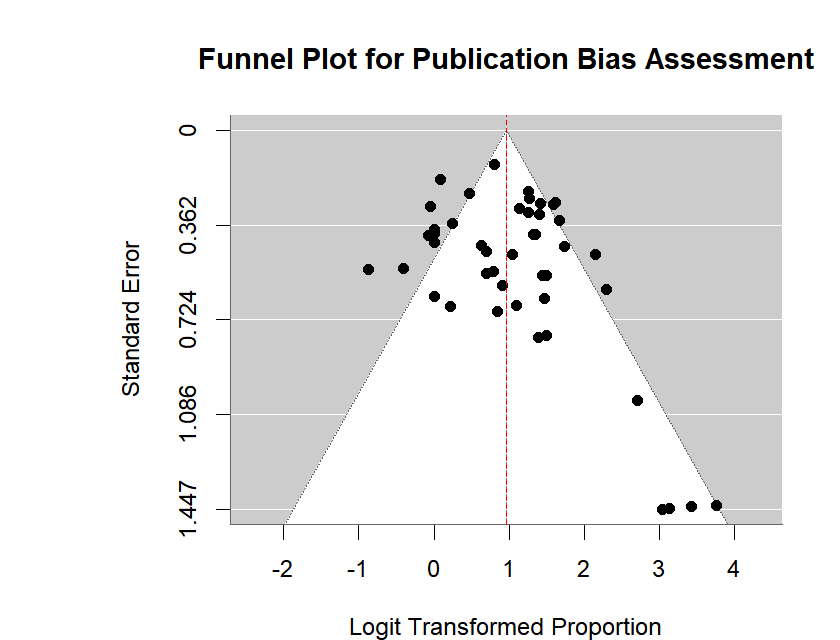


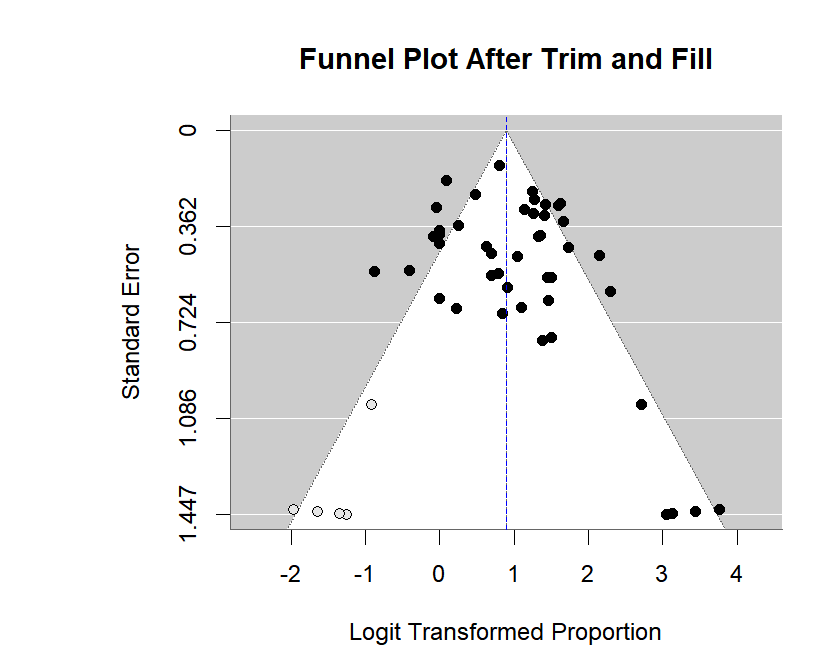


Analysis Effect_Size CI_95 p_value Tau2 I2 Egger_p_value

1 Before Imputation 0.724 [0.678, 0.766] 1.19e-17 0.344 71.2% 9.55e-03

2 After Trim and Fill 0.710 [0.662, 0.754] 2.84e-15 0.367 70.6% 9.18e-01


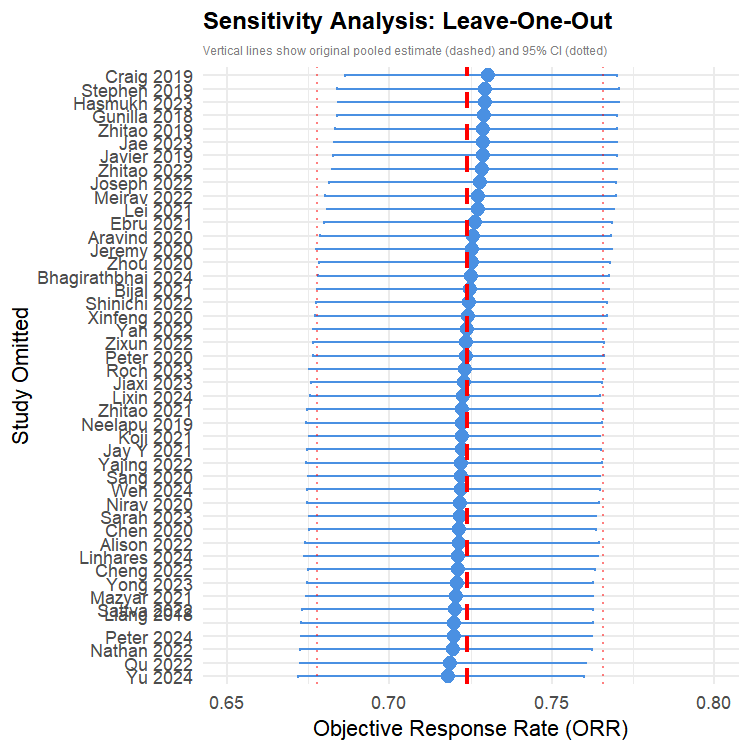

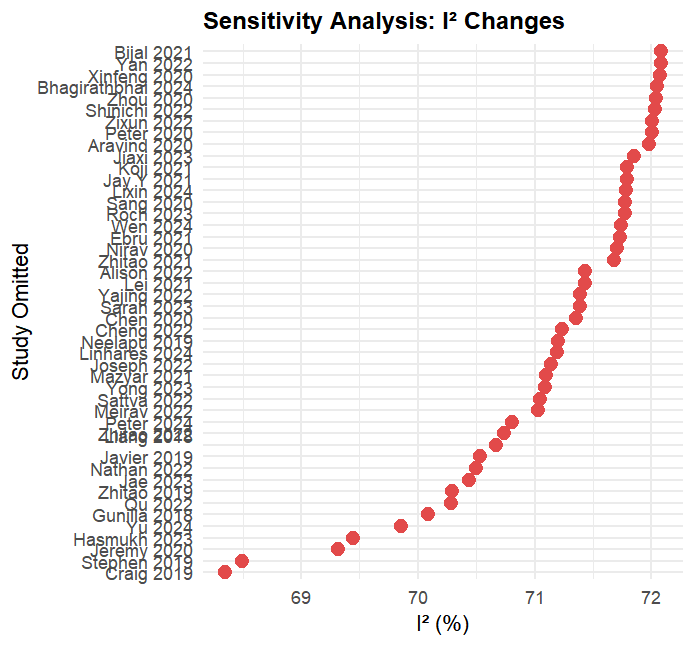


| **Study_Omitted** | **Effect_CI** | **I2** | **p_value** |
| --- | --- | --- | --- |
| Roch 2023 | 0.723 [0.675, 0.766] | 71.80% | 1.31e-16 |
| Neelapu 2019 | 0.722 [0.674, 0.765] | 71.20% | 1.73e-16 |
| Bhagirathbhai 2024 | 0.725 [0.678, 0.768] | 72% | 3.26e-17 |
| Sattva 2022 | 0.72 [0.673, 0.763] | 71% | 8.91e-17 |
| Koji 2021 | 0.722 [0.675, 0.765] | 71.80% | 6.01e-17 |
| Linhares 2024 | 0.721 [0.674, 0.764] | 71.20% | 1.56e-16 |
| Shinichi 2022 | 0.724 [0.677, 0.767] | 72% | 3.29e-17 |
| Jeremy 2020 | 0.725 [0.678, 0.769] | 69.30% | 9.25e-17 |
| Alison 2022 | 0.721 [0.674, 0.764] | 71.40% | 1.39e-16 |
| Zixun 2022 | 0.724 [0.677, 0.766] | 72% | 4.50e-17 |
| Yan 2022 | 0.724 [0.676, 0.767] | 72.10% | 6.82e-17 |
| Zhitao 2021 | 0.722 [0.675, 0.765] | 71.70% | 1.38e-16 |
| Stephen 2019 | 0.73 [0.684, 0.771] | 68.50% | 1.06e-18 |
| Nathan 2022 | 0.72 [0.672, 0.762] | 70.50% | 1.33e-16 |
| Hasmukh 2023 | 0.73 [0.684, 0.771] | 69.40% | 7.41e-19 |
| Zhou 2020 | 0.725 [0.678, 0.768] | 72% | 3.72e-17 |
| Chen 2020 | 0.721 [0.675, 0.764] | 71.40% | 2.67e-17 |
| Xinfeng 2020 | 0.724 [0.677, 0.767] | 72.10% | 4.33e-17 |
| Zhitao 2019 | 0.729 [0.683, 0.77] | 70.30% | 1.42e-18 |
| Peter 2024 | 0.72 [0.673, 0.762] | 70.80% | 1.12e-16 |
| Joseph 2022 | 0.728 [0.681, 0.77] | 71.10% | 7.57e-18 |
| Bijal 2021 | 0.725 [0.678, 0.767] | 72.10% | 3.95e-17 |
| Zhitao 2022 | 0.728 [0.682, 0.77] | 70.70% | 2.68e-18 |
| Yong 2023 | 0.721 [0.674, 0.763] | 71.10% | 2.64e-17 |
| Wen 2024 | 0.722 [0.675, 0.765] | 71.70% | 1.12e-16 |
| Jiaxi 2023 | 0.723 [0.676, 0.765] | 71.90% | 4.22e-17 |
| Jae 2023 | 0.729 [0.683, 0.77] | 70.40% | 2.00e-18 |
| Ebru 2021 | 0.726 [0.68, 0.768] | 71.70% | 1.14e-17 |
| Craig 2019 | 0.73 [0.686, 0.77] | 68.30% | 4.52e-20 |
| Lei 2021 | 0.727 [0.681, 0.769] | 71.40% | 6.06e-18 |
| Gunilla 2018 | 0.729 [0.684, 0.77] | 70.10% | 6.86e-19 |
| Meirav 2022 | 0.727 [0.68, 0.77] | 71% | 2.04e-17 |
| Yu 2024 | 0.718 [0.672, 0.76] | 69.90% | 4.81e-17 |
| Sang 2020 | 0.722 [0.675, 0.765] | 71.80% | 7.36e-17 |
| Nirav 2020 | 0.722 [0.675, 0.764] | 71.70% | 7.50e-17 |
| Sarah 2023 | 0.722 [0.675, 0.764] | 71.40% | 2.65e-17 |
| Peter 2020 | 0.724 [0.677, 0.766] | 72% | 4.50e-17 |
| Lixin 2024 | 0.722 [0.676, 0.765] | 71.80% | 4.51e-17 |
| Yajing 2022 | 0.722 [0.674, 0.765] | 71.40% | 1.62e-16 |
| Cheng 2022 | 0.721 [0.675, 0.763] | 71.20% | 2.67e-17 |
| Mazyar 2021 | 0.721 [0.674, 0.763] | 71.10% | 3.39e-17 |
| Javier 2019 | 0.729 [0.683, 0.77] | 70.50% | 2.19e-18 |
| Qu 2022 | 0.719 [0.672, 0.761] | 70.30% | 4.39e-17 |
| Jay Y 2021 | 0.722 [0.675, 0.765] | 71.80% | 1.10e-16 |
| Aravind 2020 | 0.726 [0.679, 0.768] | 72% | 3.27e-17 |
| Liang 2018 | 0.72 [0.673, 0.763] | 70.70% | 1.35e-16 |

Analysis Effect_Size CI_95 p_value Tau2 I2 Egger_p_value

1 Before Imputation 0.724 [0.678, 0.766] 1.19e-17 0.344 71.2% 9.55e-03

2 After Trim and Fill 0.710 [0.662, 0.754] 2.84e-15 0.367 70.6% 9.18e-01

Figure S4.2 Overall CAR-T CR


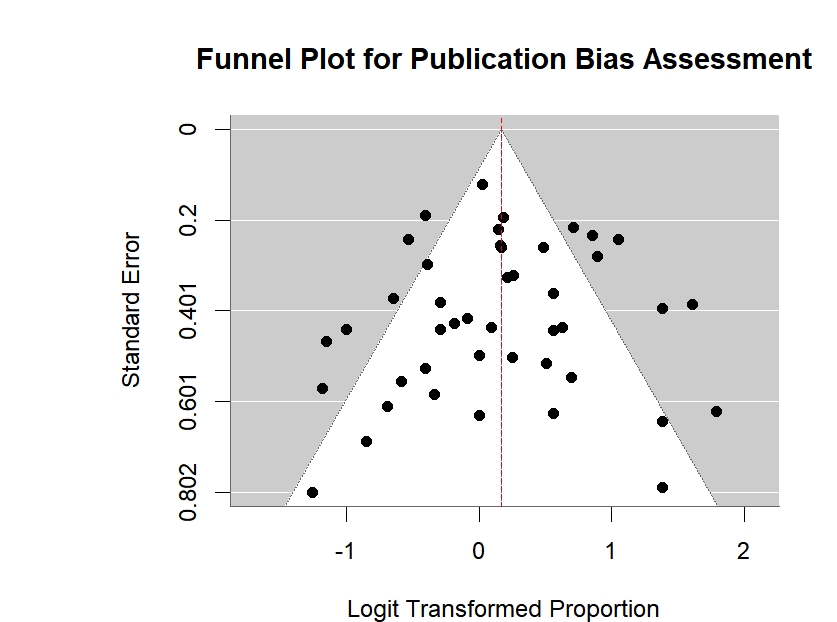


Analysis Effect_Size CI_95 p_value Tau2 I2 Egger_p_value

1 Before Imputation 0.542 [0.494, 0.59] 8.80e-02 0.275 71.4% 5.00e-01


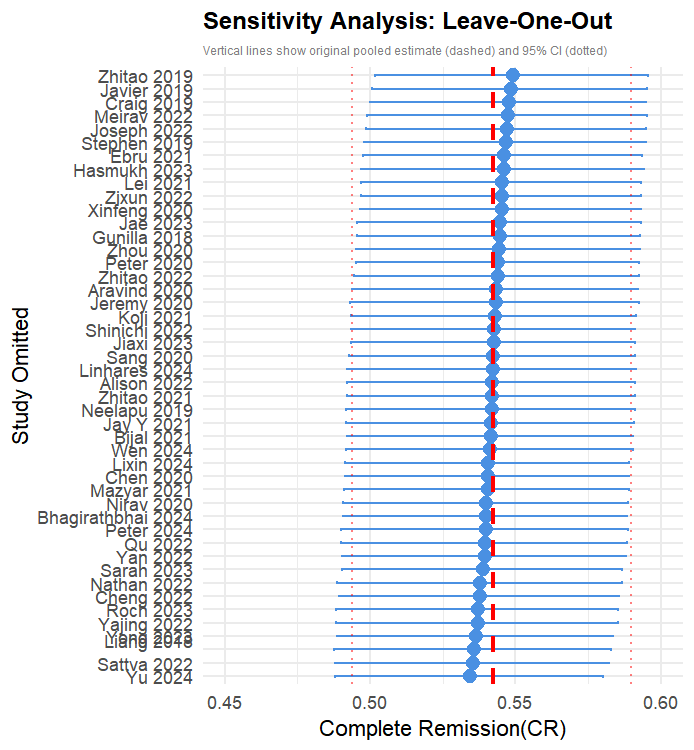


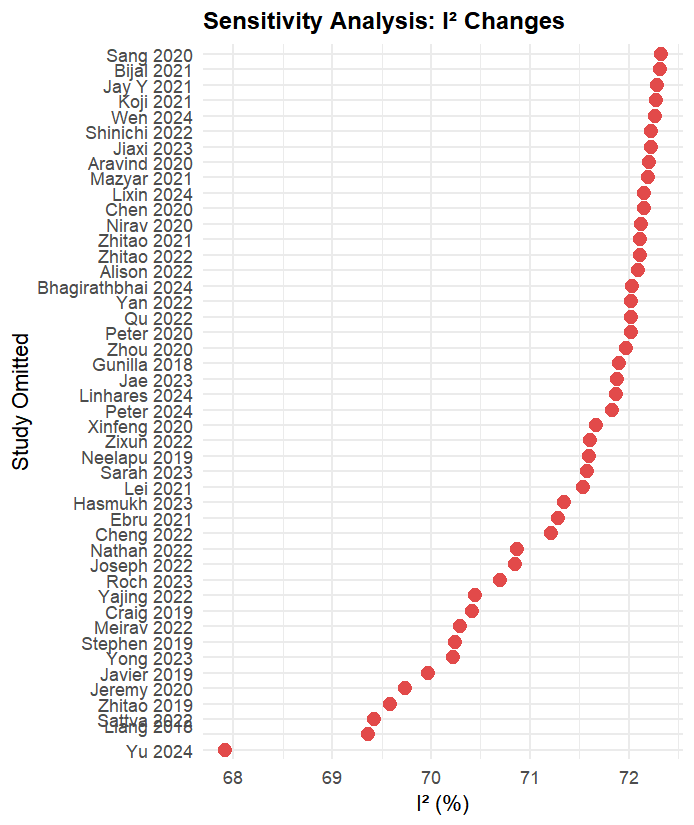


The sensitivity analysis showed that after sequentially removing individual studies, the heterogeneity index I² fluctuated between 68% and 72%, with a small variation, further confirming the robustness of the pooled results.

| **Study_Omitted** | **Effect_CI** | **I2** | **p_value** |
| --- | --- | --- | --- |
| Roch 2023 | 0.537 [0.488, 0.585] | 70.70% | 1.36e-01 |
| Neelapu 2019 | 0.542 [0.492, 0.591] | 71.60% | 1.01e-01 |
| Bhagirathbhai 2024 | 0.54 [0.491, 0.588] | 72% | 1.12e-01 |
| Sattva 2022 | 0.535 [0.488, 0.582] | 69.40% | 1.43e-01 |
| Koji 2021 | 0.543 [0.493, 0.591] | 72.30% | 8.89e-02 |
| Linhares 2024 | 0.542 [0.492, 0.591] | 71.90% | 9.86e-02 |
| Shinichi 2022 | 0.543 [0.494, 0.591] | 72.20% | 8.88e-02 |
| Jeremy 2020 | 0.543 [0.493, 0.593] | 69.70% | 9.08e-02 |
| Alison 2022 | 0.542 [0.492, 0.591] | 72.10% | 9.91e-02 |
| Zixun 2022 | 0.545 [0.497, 0.593] | 71.60% | 6.73e-02 |
| Yan 2022 | 0.54 [0.49, 0.588] | 72% | 1.15e-01 |
| Zhitao 2021 | 0.542 [0.492, 0.591] | 72.10% | 9.94e-02 |
| Stephen 2019 | 0.547 [0.498, 0.595] | 70.20% | 6.12e-02 |
| Nathan 2022 | 0.538 [0.489, 0.587] | 70.90% | 1.32e-01 |
| Hasmukh 2023 | 0.546 [0.497, 0.594] | 71.30% | 6.66e-02 |
| Zhou 2020 | 0.545 [0.495, 0.593] | 72% | 7.64e-02 |
| Chen 2020 | 0.541 [0.491, 0.589] | 72.20% | 1.05e-01 |
| Xinfeng 2020 | 0.545 [0.497, 0.593] | 71.70% | 6.85e-02 |
| Zhitao 2019 | 0.549 [0.502, 0.595] | 69.60% | 4.23e-02 |
| Peter 2024 | 0.54 [0.49, 0.589] | 71.80% | 1.17e-01 |
| Joseph 2022 | 0.547 [0.499, 0.595] | 70.80% | 5.66e-02 |
| Bijal 2021 | 0.542 [0.492, 0.59] | 72.30% | 9.84e-02 |
| Zhitao 2022 | 0.544 [0.495, 0.593] | 72.10% | 8.08e-02 |
| Yong 2023 | 0.536 [0.489, 0.583] | 70.20% | 1.34e-01 |
| Wen 2024 | 0.541 [0.492, 0.59] | 72.30% | 1.03e-01 |
| Jiaxi 2023 | 0.543 [0.494, 0.591] | 72.20% | 8.88e-02 |
| Jae 2023 | 0.545 [0.495, 0.593] | 71.90% | 7.48e-02 |
| Ebru 2021 | 0.546 [0.498, 0.593] | 71.30% | 6.18e-02 |
| Craig 2019 | 0.548 [0.5, 0.595] | 70.40% | 4.99e-02 |
| Lei 2021 | 0.546 [0.497, 0.593] | 71.50% | 6.62e-02 |
| Gunilla 2018 | 0.545 [0.496, 0.593] | 71.90% | 7.39e-02 |
| Meirav 2022 | 0.548 [0.499, 0.595] | 70.30% | 5.51e-02 |
| Yu 2024 | 0.534 [0.488, 0.58] | 67.90% | 1.48e-01 |
| Sang 2020 | 0.542 [0.493, 0.591] | 72.30% | 9.32e-02 |
| Nirav 2020 | 0.54 [0.491, 0.589] | 72.10% | 1.12e-01 |
| Sarah 2023 | 0.539 [0.49, 0.587] | 71.60% | 1.17e-01 |
| Peter 2020 | 0.544 [0.495, 0.592] | 72% | 7.78e-02 |
| Lixin 2024 | 0.541 [0.491, 0.589] | 72.20% | 1.05e-01 |
| Yajing 2022 | 0.537 [0.488, 0.585] | 70.40% | 1.38e-01 |
| Cheng 2022 | 0.538 [0.49, 0.586] | 71.20% | 1.25e-01 |
| Mazyar 2021 | 0.541 [0.491, 0.589] | 72.20% | 1.07e-01 |
| Javier 2019 | 0.549 [0.501, 0.595] | 70% | 4.60e-02 |
| Qu 2022 | 0.54 [0.49, 0.588] | 72% | 1.16e-01 |
| Jay Y 2021 | 0.542 [0.492, 0.591] | 72.30% | 1.00e-01 |
| Aravind 2020 | 0.543 [0.494, 0.592] | 72.20% | 8.50e-02 |
| Liang 2018 | 0.536 [0.488, 0.583] | 69.40% | 1.45e-01 |

| Min_Effect | Max_Effect | Original_Effect | Range | Min_I2 | Max_I2 | I2_Range |
| --- | --- | --- | --- | --- | --- | --- |
| 0.5342557 | 0.5490347 | 0.542162 | 0.01477894 | 67.9112 | 72.32518 | 4.413972 |

Range of effect sizes in sensitivity analysis: 0.534 to 0.549

Figure S4.3 Overall CAR-T CRS≥ Grade 3


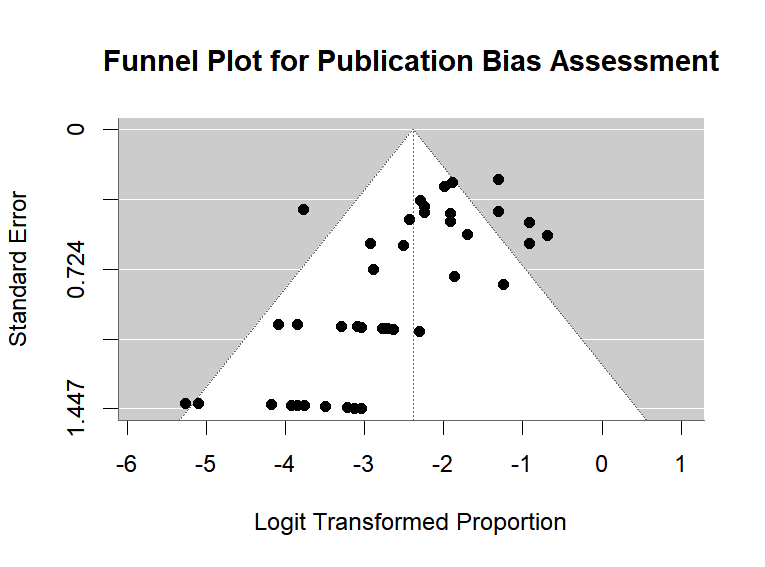


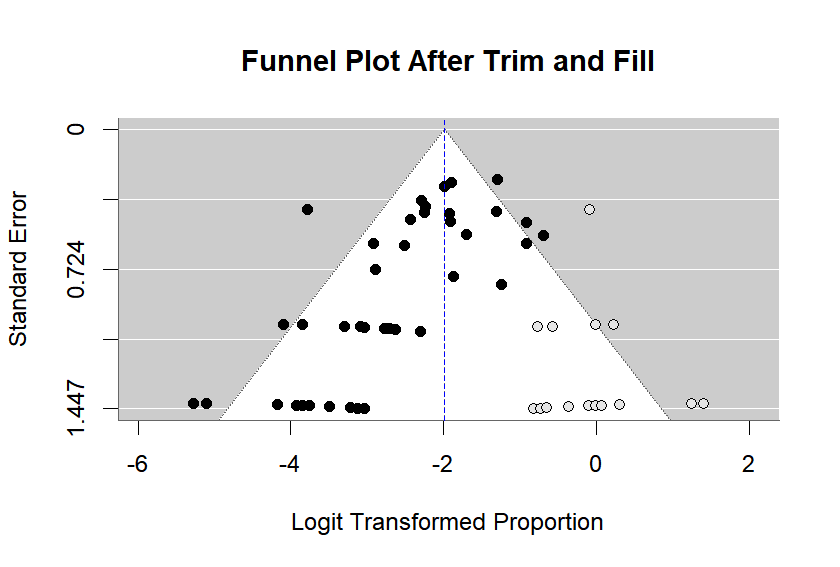


Analysis Effect_Size CI_95 p_value Tau2 I2 Egger_p_value

1 Before Imputation 0.084 [0.064, 0.11] 1.52e-55 0.43 53.2% 7.28e-06

2 After Trim and Fill 0.120 [0.091, 0.156] 3.88e-37 0.70 60.1% 4.67e-01


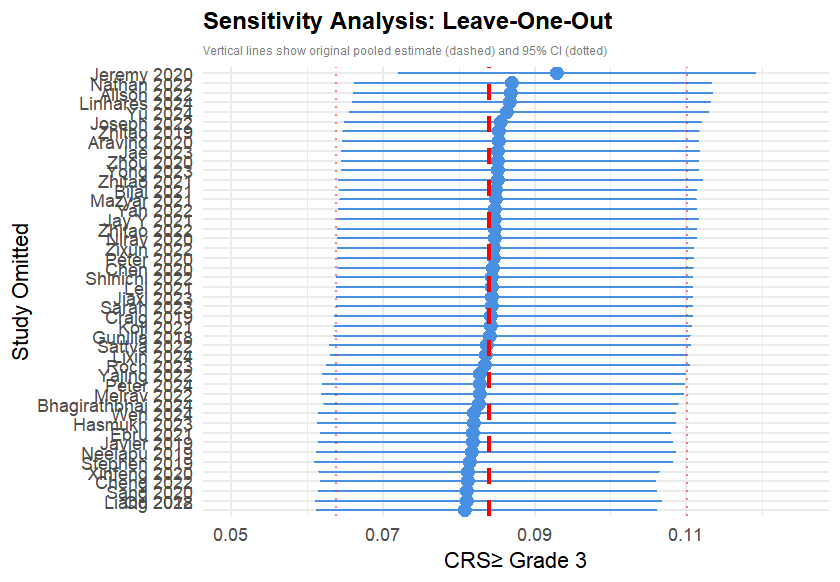


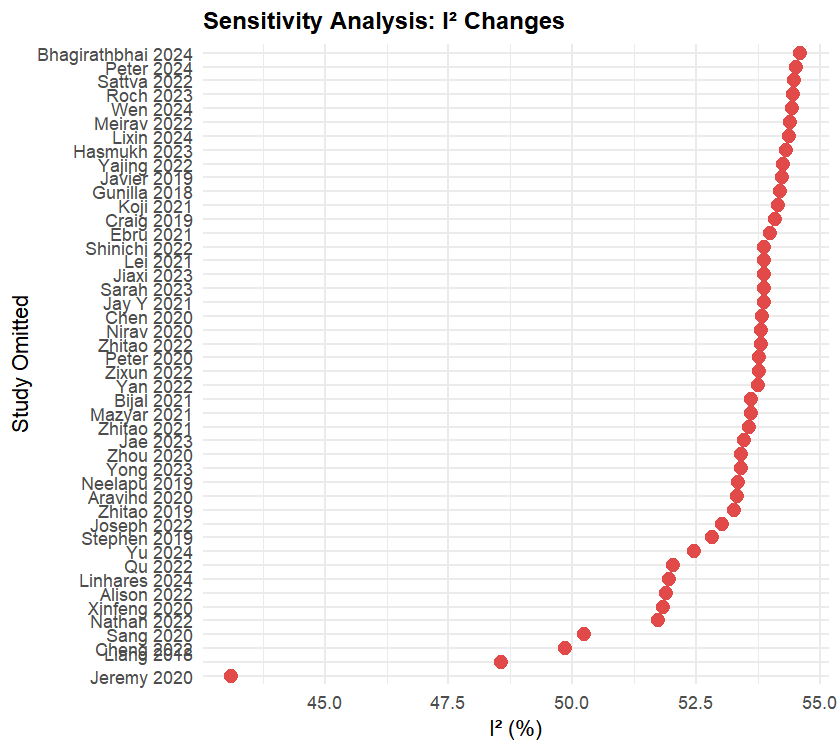


Range of effect sizes in sensitivity analysis: 0.081 to 0.093

| **Study_Omitted** | **Effect_CI** | **I2** | **p_value** |
| --- | --- | --- | --- |
| Roch 2023 | 0.083 [0.063, 0.11] | 54.50% | 4.17e-52 |
| Neelapu 2019 | 0.082 [0.061, 0.108] | 53.30% | 2.89e-52 |
| Bhagirathbhai 2024 | 0.083 [0.062, 0.109] | 54.60% | 9.08e-54 |
| Sattva 2022 | 0.084 [0.063, 0.111] | 54.50% | 1.14e-52 |
| Koji 2021 | 0.084 [0.064, 0.111] | 54.20% | 5.57e-54 |
| Linhares 2024 | 0.087 [0.066, 0.113] | 52% | 1.91e-55 |
| Shinichi 2022 | 0.084 [0.064, 0.111] | 53.90% | 1.33e-54 |
| Jeremy 2020 | 0.093 [0.072, 0.119] | 43.10% | 1.13e-58 |
| Alison 2022 | 0.087 [0.066, 0.113] | 51.90% | 5.64e-55 |
| Zixun 2022 | 0.085 [0.064, 0.111] | 53.80% | 1.29e-54 |
| Yan 2022 | 0.085 [0.064, 0.111] | 53.80% | 4.78e-54 |
| Zhitao 2021 | 0.085 [0.064, 0.112] | 53.60% | 6.87e-53 |
| Stephen 2019 | 0.082 [0.061, 0.108] | 52.80% | 1.35e-52 |
| Nathan 2022 | 0.087 [0.066, 0.113] | 51.70% | 1.40e-55 |
| Hasmukh 2023 | 0.082 [0.061, 0.108] | 54.30% | 7.25e-53 |
| Zhou 2020 | 0.085 [0.065, 0.112] | 53.40% | 1.01e-54 |
| Chen 2020 | 0.085 [0.064, 0.111] | 53.80% | 1.31e-54 |
| Xinfeng 2020 | 0.081 [0.062, 0.106] | 51.80% | 3.68e-57 |
| Zhitao 2019 | 0.085 [0.065, 0.112] | 53.30% | 8.90e-55 |
| Peter 2024 | 0.083 [0.062, 0.11] | 54.50% | 4.16e-52 |
| Joseph 2022 | 0.086 [0.065, 0.112] | 53% | 7.09e-55 |
| Bijal 2021 | 0.085 [0.064, 0.111] | 53.60% | 1.18e-54 |
| Zhitao 2022 | 0.085 [0.064, 0.111] | 53.80% | 4.96e-54 |
| Yong 2023 | 0.085 [0.065, 0.112] | 53.40% | 1.01e-54 |
| Wen 2024 | 0.082 [0.062, 0.109] | 54.50% | 5.45e-53 |
| Jiaxi 2023 | 0.084 [0.064, 0.111] | 53.90% | 1.33e-54 |
| Jae 2023 | 0.085 [0.065, 0.112] | 53.50% | 3.79e-54 |
| Ebru 2021 | 0.082 [0.062, 0.108] | 54% | 5.33e-55 |
| Craig 2019 | 0.084 [0.064, 0.111] | 54.10% | 5.57e-54 |
| Lei 2021 | 0.084 [0.064, 0.111] | 53.90% | 1.33e-54 |
| Gunilla 2018 | 0.084 [0.064, 0.111] | 54.20% | 5.52e-54 |
| Meirav 2022 | 0.083 [0.062, 0.11] | 54.40% | 5.52e-52 |
| Yu 2024 | 0.086 [0.066, 0.113] | 52.50% | 1.20e-54 |
| Sang 2020 | 0.081 [0.062, 0.106] | 50.20% | 1.97e-58 |
| Nirav 2020 | 0.085 [0.064, 0.111] | 53.80% | 4.96e-54 |
| Sarah 2023 | 0.084 [0.064, 0.111] | 53.90% | 1.33e-54 |
| Peter 2020 | 0.085 [0.064, 0.111] | 53.80% | 1.29e-54 |
| Lixin 2024 | 0.084 [0.063, 0.11] | 54.40% | 4.50e-54 |
| Yajing 2022 | 0.083 [0.062, 0.11] | 54.30% | 8.35e-52 |
| Cheng 2022 | 0.081 [0.062, 0.106] | 49.90% | 2.85e-59 |
| Mazyar 2021 | 0.085 [0.064, 0.111] | 53.60% | 1.18e-54 |
| Javier 2019 | 0.082 [0.062, 0.108] | 54.20% | 9.51e-54 |
| Qu 2022 | 0.081 [0.061, 0.107] | 52% | 7.31e-56 |
| Jay Y 2021 | 0.085 [0.064, 0.112] | 53.90% | 2.92e-53 |
| Aravind 2020 | 0.085 [0.065, 0.112] | 53.30% | 9.49e-55 |
| Liang 2018 | 0.081 [0.061, 0.106] | 48.60% | 2.77e-57 |

| Min_Effect | Max_Effect | Original_Effect | Range | Min_I2 | Max_I2 | I2_Range |
| --- | --- | --- | --- | --- | --- | --- |
| 0.08083699 | 0.0929509 | 0.08404677 | 0.01211391 | 43.1127 | 54.60729 | 11.49459 |

Figure S4.4 Overall CAR-T Neurotoxicity≥ Grade 3


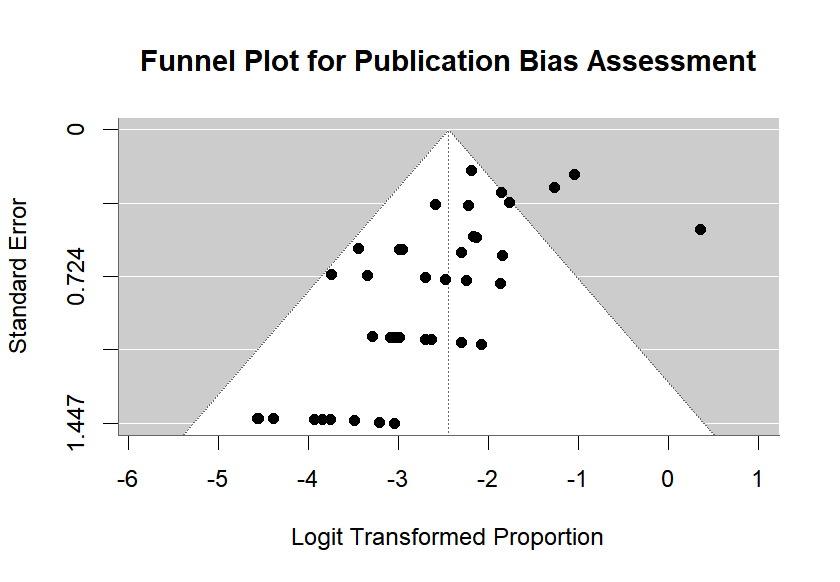


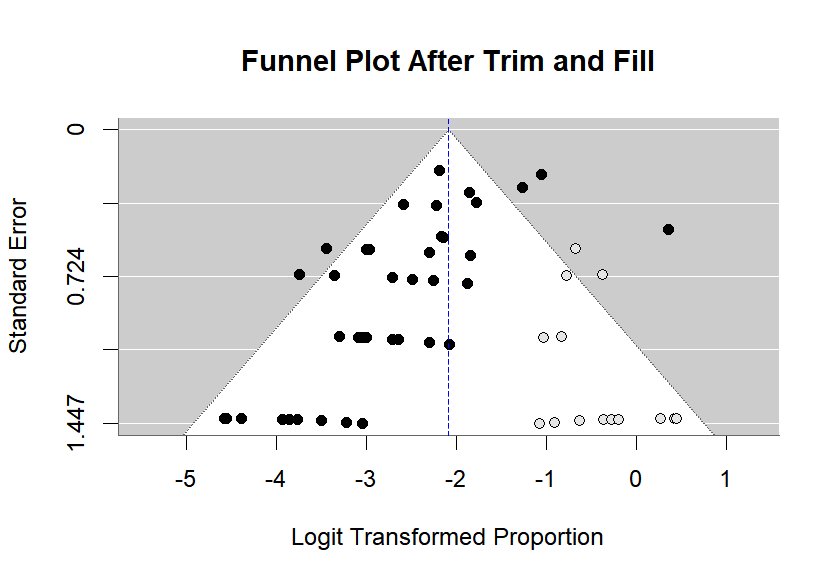


Analysis Effect_Size CI_95 p_value Tau2 I2 Egger_p_value

1 Before Imputation 0.080 [0.06, 0.106] 8.96e-52 0.503 60.5% 1.24e-05

2 After Trim and Fill 0.111 [0.085, 0.143] 1.66e-44 0.556 57.6% 3.02e-01


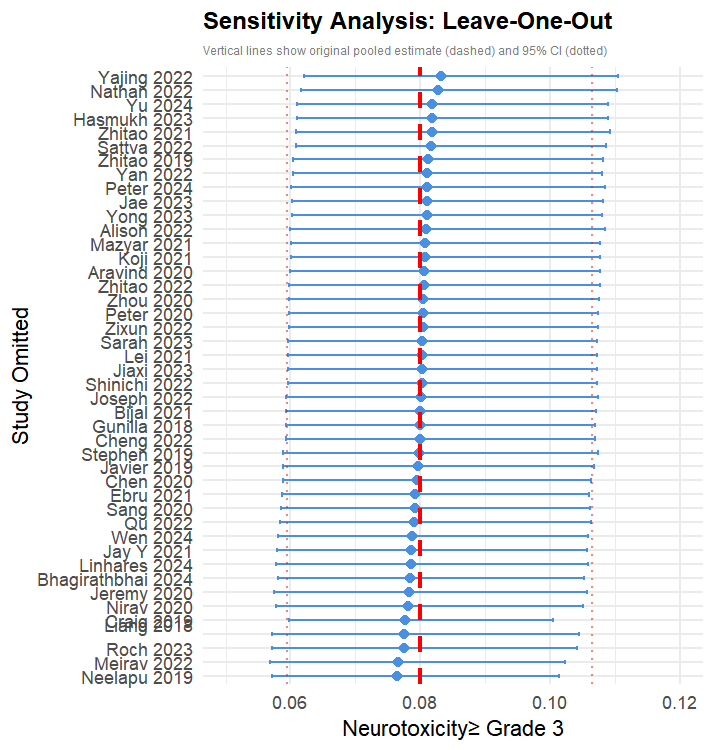


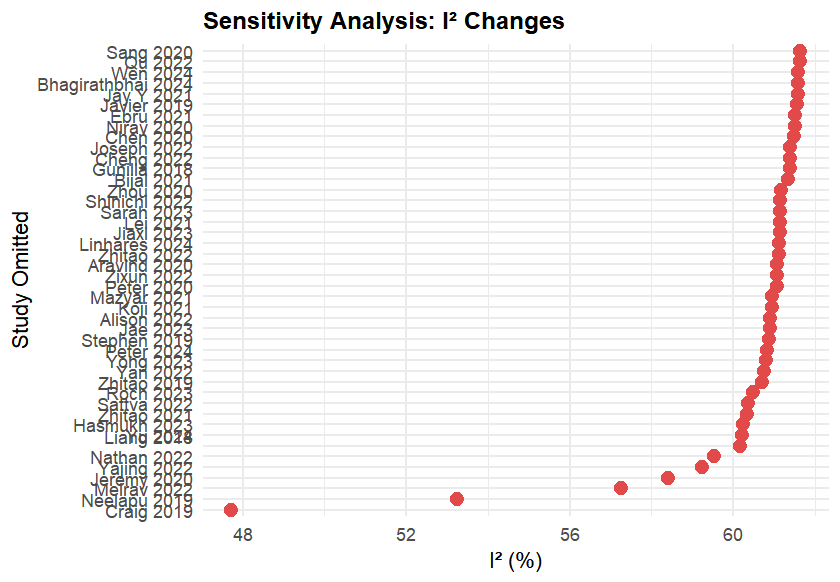


| **Study_Omitted** | **Effect_CI** | **I2** | **p_value** |
| --- | --- | --- | --- |
| Roch 2023 | 0.077 [0.057, 0.104] | 60.50% | 4.95e-50 |
| Neelapu 2019 | 0.076 [0.057, 0.101] | 53.20% | 8.15e-56 |
| Bhagirathbhai 2024 | 0.078 [0.058, 0.105] | 61.60% | 2.47e-50 |
| Sattva 2022 | 0.082 [0.061, 0.109] | 60.40% | 5.59e-51 |
| Koji 2021 | 0.081 [0.06, 0.108] | 61% | 7.80e-51 |
| Linhares 2024 | 0.079 [0.058, 0.106] | 61.10% | 8.05e-49 |
| Shinichi 2022 | 0.08 [0.06, 0.107] | 61.10% | 7.53e-51 |
| Jeremy 2020 | 0.078 [0.057, 0.106] | 58.40% | 1.90e-48 |
| Alison 2022 | 0.081 [0.06, 0.108] | 60.90% | 3.05e-49 |
| Zixun 2022 | 0.08 [0.06, 0.107] | 61.10% | 7.76e-51 |
| Yan 2022 | 0.081 [0.06, 0.108] | 60.80% | 7.29e-51 |
| Zhitao 2021 | 0.082 [0.061, 0.109] | 60.30% | 7.58e-50 |
| Stephen 2019 | 0.08 [0.059, 0.107] | 60.90% | 2.09e-48 |
| Nathan 2022 | 0.083 [0.062, 0.11] | 59.50% | 8.35e-50 |
| Hasmukh 2023 | 0.082 [0.061, 0.109] | 60.20% | 4.99e-51 |
| Zhou 2020 | 0.08 [0.06, 0.108] | 61.20% | 2.76e-50 |
| Chen 2020 | 0.079 [0.059, 0.106] | 61.50% | 1.76e-50 |
| Zhitao 2019 | 0.081 [0.06, 0.108] | 60.70% | 7.09e-51 |
| Peter 2024 | 0.081 [0.06, 0.109] | 60.80% | 2.92e-49 |
| Joseph 2022 | 0.08 [0.059, 0.107] | 61.40% | 1.30e-49 |
| Bijal 2021 | 0.08 [0.059, 0.107] | 61.30% | 2.54e-50 |
| Zhitao 2022 | 0.081 [0.06, 0.108] | 61.10% | 2.76e-50 |
| Yong 2023 | 0.081 [0.06, 0.108] | 60.80% | 7.47e-51 |
| Wen 2024 | 0.079 [0.058, 0.106] | 61.60% | 2.24e-49 |
| Jiaxi 2023 | 0.08 [0.06, 0.107] | 61.10% | 7.53e-51 |
| Jae 2023 | 0.081 [0.06, 0.108] | 60.90% | 2.58e-50 |
| Ebru 2021 | 0.079 [0.059, 0.106] | 61.50% | 1.29e-50 |
| Craig 2019 | 0.078 [0.06, 0.1] | 47.70% | 2.60e-66 |
| Lei 2021 | 0.08 [0.06, 0.107] | 61.10% | 7.53e-51 |
| Gunilla 2018 | 0.08 [0.059, 0.107] | 61.40% | 2.43e-50 |
| Meirav 2022 | 0.077 [0.057, 0.102] | 57.20% | 5.35e-53 |
| Yu 2024 | 0.082 [0.061, 0.109] | 60.20% | 4.91e-51 |
| Sang 2020 | 0.079 [0.059, 0.106] | 61.60% | 7.08e-50 |
| Nirav 2020 | 0.078 [0.058, 0.105] | 61.50% | 3.74e-50 |
| Sarah 2023 | 0.08 [0.06, 0.107] | 61.10% | 7.53e-51 |
| Peter 2020 | 0.08 [0.06, 0.107] | 61.10% | 7.76e-51 |
| Yajing 2022 | 0.083 [0.062, 0.111] | 59.20% | 2.21e-50 |
| Cheng 2022 | 0.08 [0.059, 0.107] | 61.40% | 2.43e-50 |
| Mazyar 2021 | 0.081 [0.06, 0.108] | 61% | 7.80e-51 |
| Javier 2019 | 0.08 [0.059, 0.107] | 61.60% | 1.06e-49 |
| Qu 2022 | 0.079 [0.058, 0.106] | 61.60% | 1.92e-49 |
| Jay Y 2021 | 0.079 [0.058, 0.106] | 61.60% | 2.01e-49 |
| Aravind 2020 | 0.081 [0.06, 0.108] | 61.10% | 2.75e-50 |
| Liang 2018 | 0.078 [0.057, 0.104] | 60.20% | 1.15e-49 |

| Min_Effect | Max_Effect | Original_Effect | Range | Min_I2 | Max_I2 | I2_Range |
| --- | --- | --- | --- | --- | --- | --- |
| 0.07638679 | 0.08312771 | 0.07990824 | 0.006740921 | 47.72115 | 61.63874 | 13.9176 |

Figure S4.5 Overall CAR-T Infection≥ Grade 3


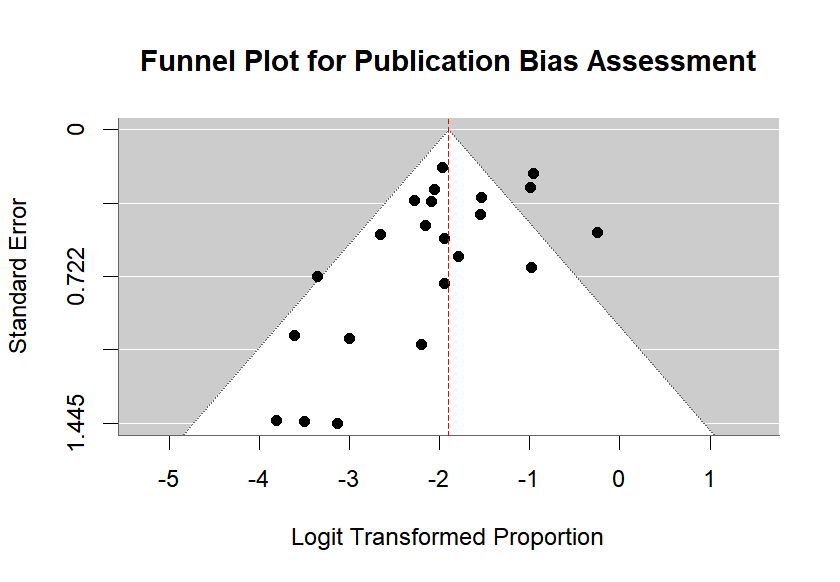


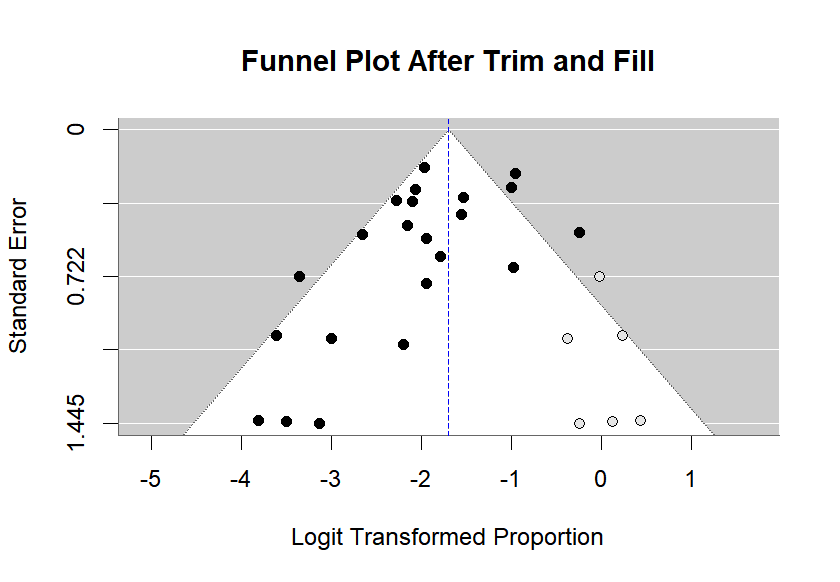


Analysis Effect_Size CI_95 p_value Tau2 I2 Egger_p_value

1 Before Imputation 0.130 [0.098, 0.171] 2.44e-31 0.300 61.3% 4.55e-03

2 After Trim and Fill 0.155 [0.118, 0.202] 2.13e-25 0.367 61.1% 8.07e-01


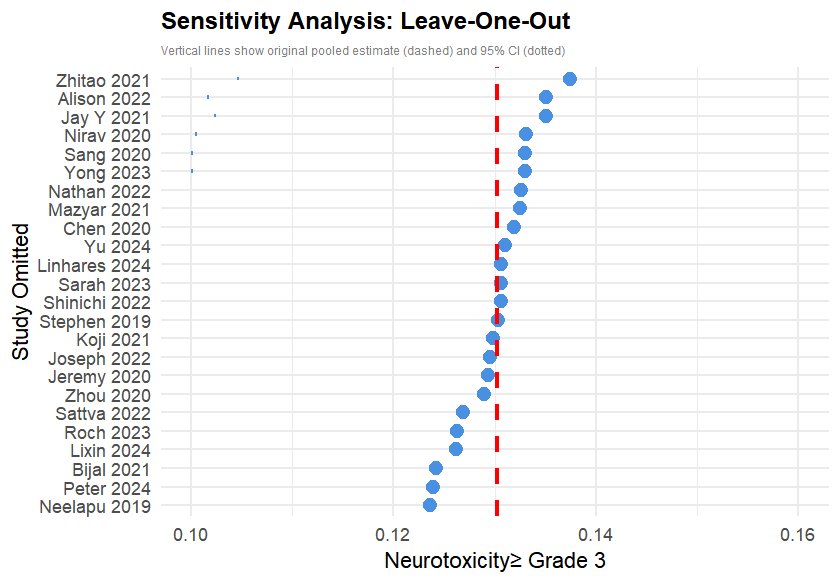


| **Study_Omitted** | **Effect_CI** | **I2** | **p_value** |
| --- | --- | --- | --- |
| Roch 2023 | 0.126 [0.093, 0.169] | 63% | 1.24e-28 |
| Neelapu 2019 | 0.124 [0.094, 0.161] | 51.40% | 1.73e-35 |
| Sattva 2022 | 0.127 [0.094, 0.169] | 63.60% | 8.04e-29 |
| Koji 2021 | 0.13 [0.097, 0.172] | 63.40% | 1.67e-29 |
| Linhares 2024 | 0.131 [0.097, 0.174] | 63% | 8.48e-28 |
| Shinichi 2022 | 0.131 [0.098, 0.172] | 62.90% | 3.65e-30 |
| Jeremy 2020 | 0.129 [0.095, 0.174] | 60.30% | 5.10e-27 |
| Alison 2022 | 0.135 [0.102, 0.177] | 60.30% | 1.41e-29 |
| Zhitao 2021 | 0.137 [0.105, 0.178] | 58% | 2.61e-31 |
| Stephen 2019 | 0.13 [0.096, 0.174] | 62.60% | 1.71e-27 |
| Nathan 2022 | 0.133 [0.099, 0.176] | 61.80% | 4.59e-28 |
| Zhou 2020 | 0.129 [0.096, 0.171] | 63.70% | 3.84e-29 |
| Chen 2020 | 0.132 [0.099, 0.173] | 62.10% | 1.10e-30 |
| Peter 2024 | 0.124 [0.093, 0.163] | 56.70% | 3.62e-33 |
| Joseph 2022 | 0.13 [0.096, 0.172] | 63.70% | 1.14e-28 |
| Bijal 2021 | 0.124 [0.095, 0.161] | 54.20% | 3.10e-37 |
| Yong 2023 | 0.133 [0.1, 0.174] | 61.70% | 2.23e-30 |
| Yu 2024 | 0.131 [0.097, 0.174] | 63.20% | 2.09e-28 |
| Sang 2020 | 0.133 [0.1, 0.174] | 61.70% | 2.23e-30 |
| Nirav 2020 | 0.133 [0.1, 0.174] | 61.40% | 7.30e-31 |
| Sarah 2023 | 0.131 [0.098, 0.172] | 62.90% | 3.65e-30 |
| Lixin 2024 | 0.126 [0.094, 0.167] | 62.60% | 7.44e-31 |
| Mazyar 2021 | 0.133 [0.1, 0.174] | 61.80% | 9.17e-31 |
| Jay Y 2021 | 0.135 [0.102, 0.176] | 60.10% | 5.89e-31 |


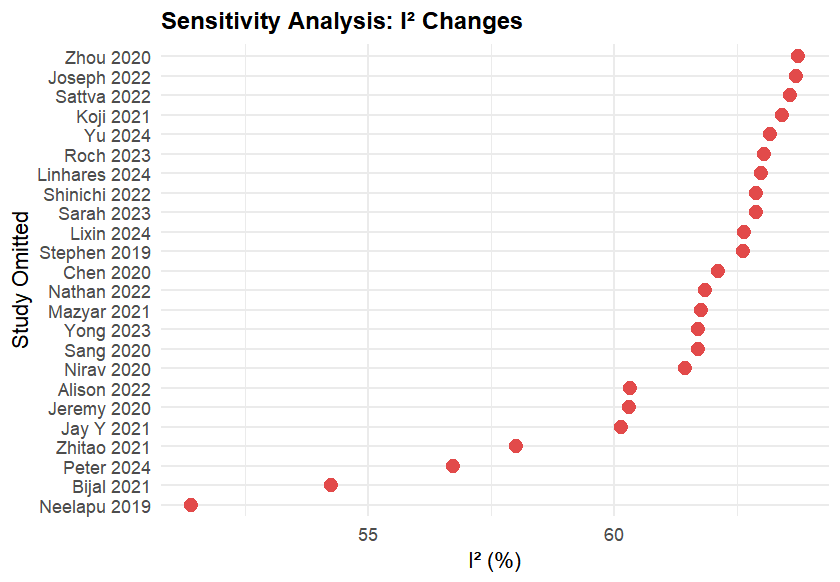


| Min_Effect | Max_Effect | Original_Effect | Range | Min_I2 | Max_I2 | I2_Range |
| --- | --- | --- | --- | --- | --- | --- |
| 0.123633 | 0.1374073 | 0.1302665 | 0.01377431 | 51.40322 | 63.73912 | 12.3359 |

Figure S4.6 Overall CAR-T ICANS≥ Grade 3


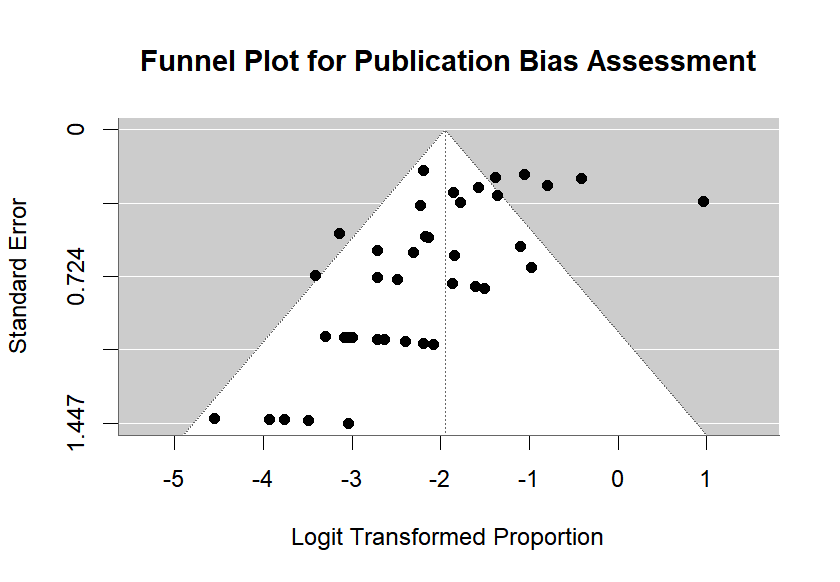


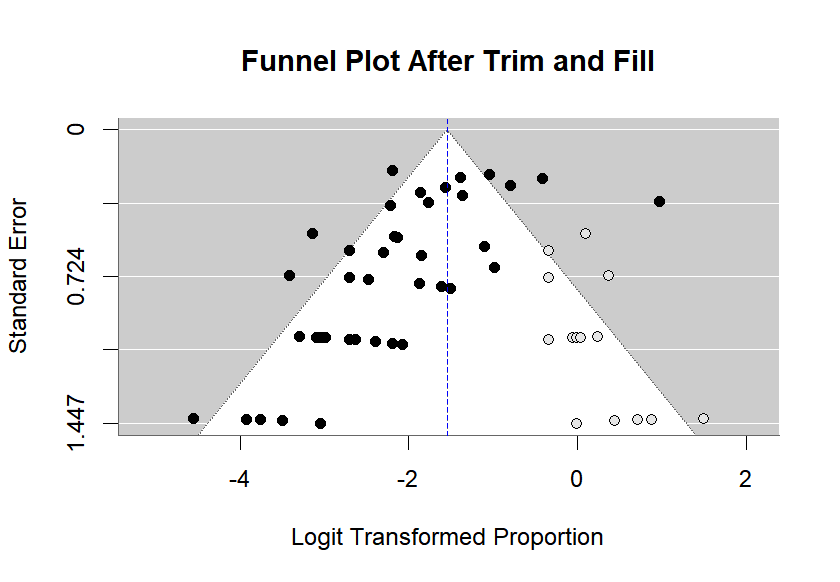


Analysis Effect_Size CI_95 p_value Tau2 I2 Egger_p_value

1 Before Imputation 0.125 [0.093, 0.165] 3.38e-31 0.672 75.6% 1.16e-05

2 After Trim and Fill 0.175 [0.134, 0.226] 1.94e-21 0.861 76.1% 4.48e-01


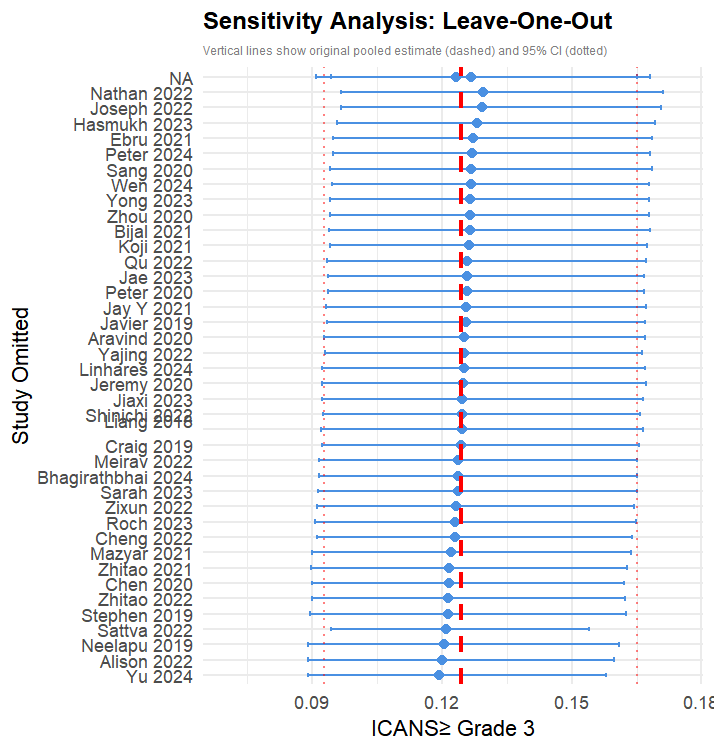


| **Study_Omitted** | **Effect_CI** | **I2** | **p_value** |
| --- | --- | --- | --- |
| Roch 2023 | 0.123 [0.091, 0.165] | 76.20% | 1.23e-29 |
| Neelapu 2019 | 0.12 [0.089, 0.161] | 74.20% | 5.80e-31 |
| Bhagirathbhai 2024 | 0.124 [0.092, 0.165] | 76.50% | 4.16e-30 |
| Sattva 2022 | 0.121 [0.094, 0.154] | 63% | 4.81e-44 |
| Koji 2021 | 0.126 [0.094, 0.168] | 75.90% | 1.62e-30 |
| Linhares 2024 | 0.125 [0.092, 0.167] | 76% | 2.38e-29 |
| Shinichi 2022 | 0.125 [0.093, 0.166] | 76.30% | 2.51e-30 |
| Jeremy 2020 | 0.125 [0.092, 0.167] | 74.50% | 3.91e-29 |
| Alison 2022 | 0.12 [0.089, 0.16] | 74.40% | 9.73e-32 |
| Zixun 2022 | 0.123 [0.091, 0.164] | 76.50% | 2.72e-30 |
| Zhitao 2021 | 0.122 [0.09, 0.163] | 75.80% | 3.35e-30 |
| Stephen 2019 | 0.121 [0.09, 0.163] | 75% | 4.05e-30 |
| Nathan 2022 | 0.129 [0.097, 0.171] | 74.80% | 6.67e-30 |
| Hasmukh 2023 | 0.128 [0.096, 0.169] | 75.40% | 1.27e-30 |
| Zhou 2020 | 0.126 [0.094, 0.168] | 76% | 3.21e-30 |
| Chen 2020 | 0.122 [0.09, 0.162] | 76.20% | 6.59e-31 |
| Peter 2024 | 0.127 [0.095, 0.168] | 75.70% | 1.54e-30 |
| Joseph 2022 | 0.129 [0.097, 0.171] | 75% | 3.62e-30 |
| Bijal 2021 | 0.126 [0.094, 0.168] | 76% | 7.05e-30 |
| Zhitao 2022 | 0.122 [0.09, 0.162] | 76.20% | 9.70e-31 |
| Yong 2023 | 0.127 [0.094, 0.168] | 75.90% | 3.19e-30 |
| Wen 2024 | 0.127 [0.095, 0.168] | 75.80% | 1.58e-30 |
| Jiaxi 2023 | 0.125 [0.092, 0.167] | 76.30% | 1.28e-29 |
| Jae 2023 | 0.126 [0.094, 0.167] | 76.10% | 1.56e-30 |
| Ebru 2021 | 0.127 [0.095, 0.169] | 75.70% | 2.98e-30 |
| Craig 2019 | 0.124 [0.092, 0.166] | 76.40% | 2.30e-30 |
| Meirav 2022 | 0.124 [0.092, 0.165] | 76.50% | 4.16e-30 |
| Yu 2024 | 0.119 [0.089, 0.158] | 72.40% | 1.77e-33 |
| Sang 2020 | 0.127 [0.094, 0.169] | 75.90% | 1.05e-29 |
| Sarah 2023 | 0.124 [0.091, 0.165] | 76.50% | 6.17e-30 |
| Peter 2020 | 0.126 [0.094, 0.167] | 76.10% | 1.56e-30 |
| Yajing 2022 | 0.125 [0.093, 0.166] | 76.30% | 2.83e-30 |
| Cheng 2022 | 0.123 [0.091, 0.164] | 76.50% | 2.23e-30 |
| Mazyar 2021 | 0.122 [0.09, 0.164] | 75.70% | 8.04e-30 |
| Javier 2019 | 0.126 [0.093, 0.167] | 76.20% | 3.11e-30 |
| Qu 2022 | 0.126 [0.094, 0.167] | 76.10% | 3.16e-30 |
| Jay Y 2021 | 0.126 [0.093, 0.167] | 76.20% | 6.99e-30 |
| Aravind 2020 | 0.125 [0.093, 0.167] | 76.30% | 1.04e-29 |
| Liang 2018 | 0.125 [0.092, 0.166] | 76.40% | 1.25e-29 |
| NA | 0.127 [0.094, 0.168] | 75.90% | 3.16e-30 |
| NA | 0.123 [0.091, 0.165] | 75.90% | 1.75e-29 |


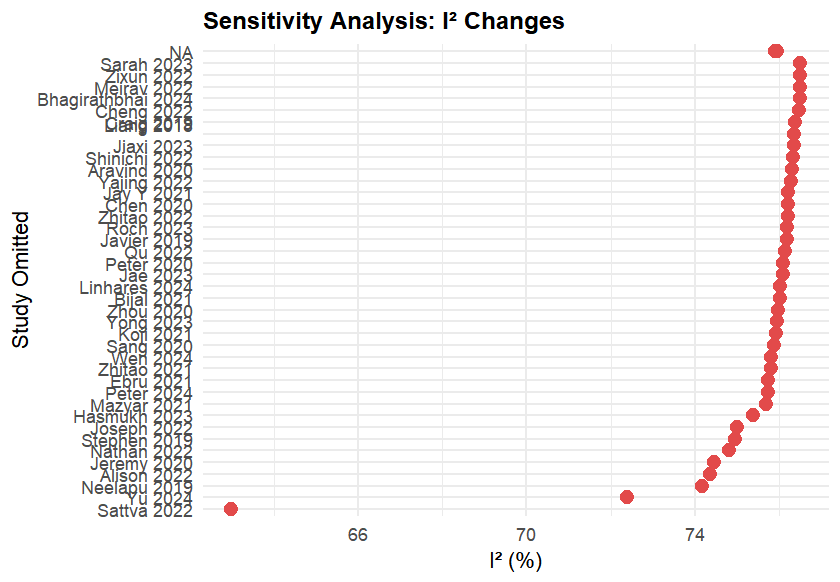


| Min_Effect | Max_Effect | Original_Effect | Range | Min_I2 | Max_I2 | I2_Range |
| --- | --- | --- | --- | --- | --- | --- |
| 0.1192782 | 0.1294133 | 0.1245201 | 0.01013509 | 62.98946 | 76.50283 | 13.51337 |


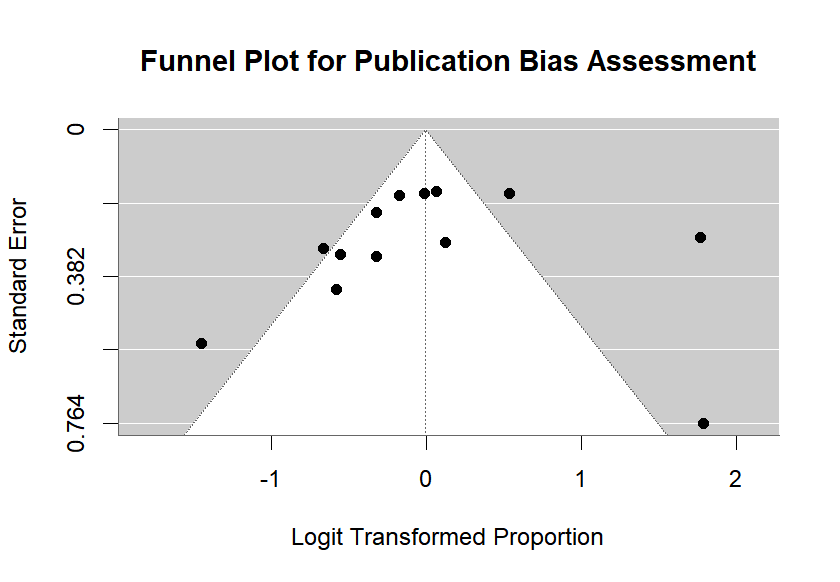


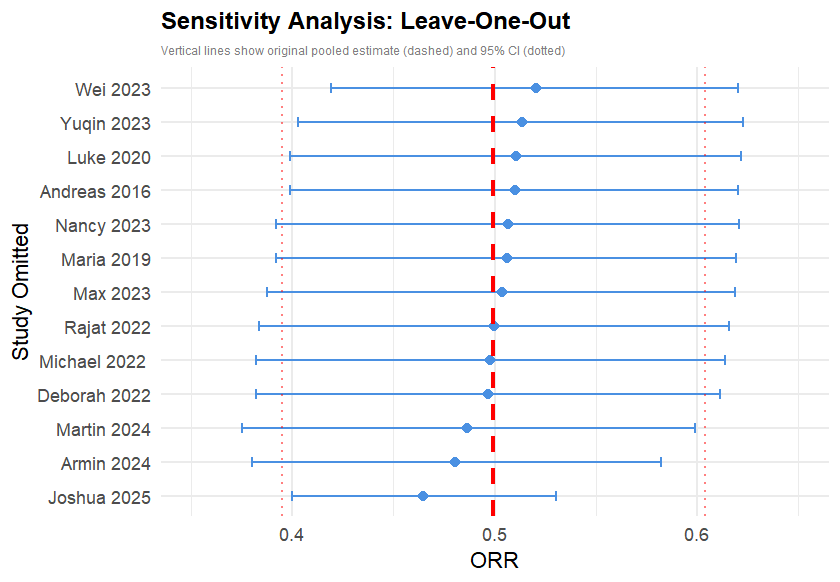


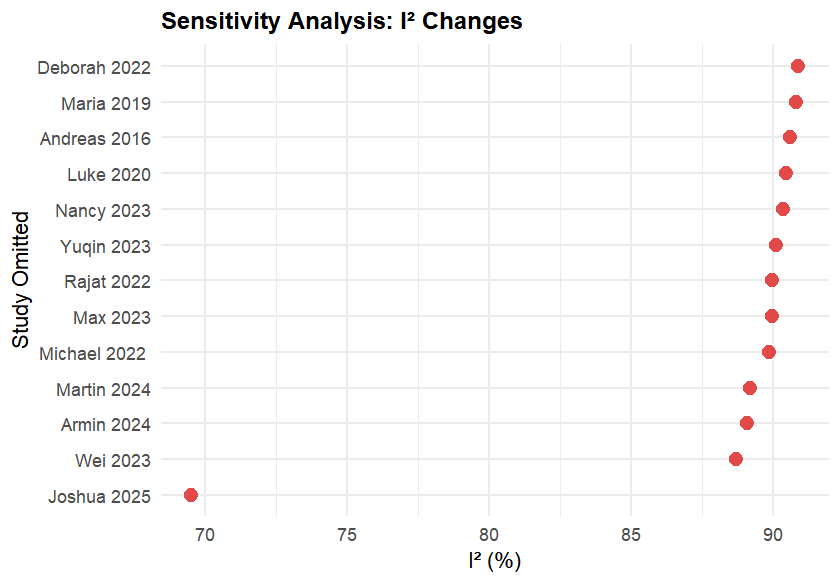


| **Study_Omitted** | **Effect_CI** | **I2** | **p_value** |
| --- | --- | --- | --- |
| Max 2023 | 0.503 [0.388, 0.619] | 90% | 9.55e-01 |
| Martin 2024 | 0.486 [0.375, 0.599] | 89.20% | 8.16e-01 |
| Joshua 2025 | 0.465 [0.4, 0.53] | 69.50% | 2.91e-01 |
| Yuqin 2023 | 0.513 [0.403, 0.623] | 90.10% | 8.15e-01 |
| Michael 2022 | 0.498 [0.382, 0.614] | 89.90% | 9.71e-01 |
| Nancy 2023 | 0.507 [0.392, 0.621] | 90.40% | 9.10e-01 |
| Rajat 2022 | 0.5 [0.384, 0.616] | 90% | 9.95e-01 |
| Maria 2019 | 0.506 [0.392, 0.619] | 90.80% | 9.20e-01 |
| Armin 2024 | 0.48 [0.38, 0.582] | 89.10% | 7.05e-01 |
| Deborah 2022 | 0.497 [0.382, 0.611] | 90.90% | 9.54e-01 |
| Luke 2020 | 0.511 [0.399, 0.622] | 90.50% | 8.52e-01 |
| Andreas 2016 | 0.51 [0.399, 0.62] | 90.60% | 8.61e-01 |
| Wei 2023 | 0.521 [0.419, 0.62] | 88.70% | 6.93e-01 |

| Min_Effect | Max_Effect | Original_Effect | Range | Min_I2 | Max_I2 | I2_Range |
| --- | --- | --- | --- | --- | --- | --- |
| 0.4645439 | 0.5206004 | 0.4993452 | 0.05605652 | 69.51718 | 90.90199 | 21.3848 |

Figure S5.2 Overall Bispecific antibodies CR


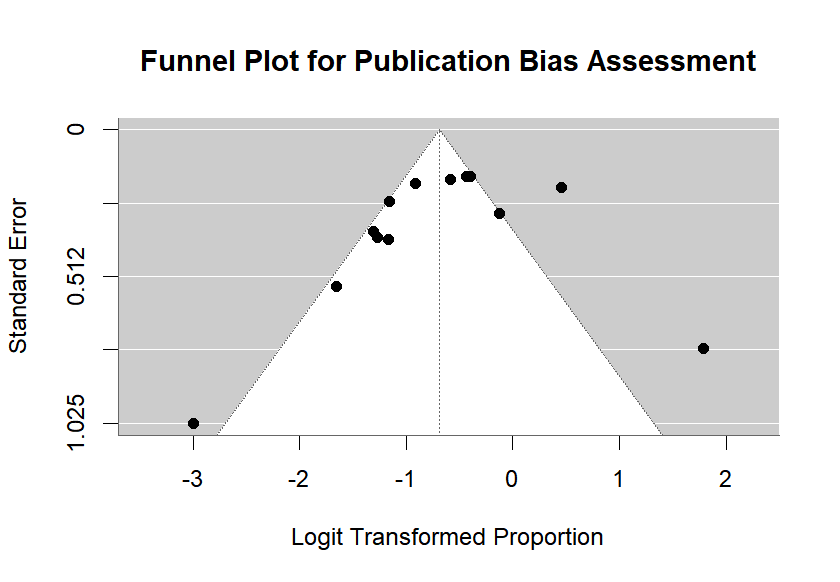


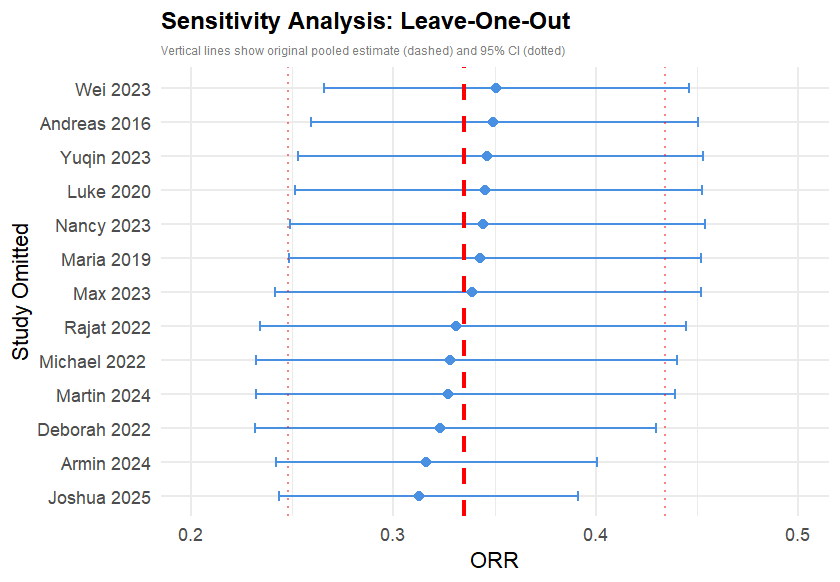


| **Study_Omitted** | **Effect_CI** | **I2** | **p_value** |
| --- | --- | --- | --- |
| Max 2023 | 0.339 [0.241, 0.452] | 89.10% | 5.86e-03 |
| Martin 2024 | 0.327 [0.232, 0.439] | 88.60% | 3.00e-03 |
| Joshua 2025 | 0.313 [0.243, 0.391] | 78.80% | 7.93e-06 |
| Yuqin 2023 | 0.346 [0.253, 0.453] | 88.90% | 5.36e-03 |
| Michael 2022 | 0.328 [0.232, 0.44] | 88.70% | 3.21e-03 |
| Nancy 2023 | 0.344 [0.249, 0.454] | 89% | 6.02e-03 |
| Rajat 2022 | 0.331 [0.234, 0.445] | 89.10% | 4.15e-03 |
| Maria 2019 | 0.343 [0.248, 0.452] | 89.50% | 5.29e-03 |
| Armin 2024 | 0.316 [0.242, 0.4] | 84.50% | 4.10e-05 |
| Deborah 2022 | 0.323 [0.232, 0.43] | 89.20% | 1.50e-03 |
| Luke 2020 | 0.345 [0.251, 0.453] | 89.20% | 5.30e-03 |
| Andreas 2016 | 0.349 [0.259, 0.451] | 88.20% | 4.08e-03 |
| Wei 2023 | 0.351 [0.266, 0.446] | 87.20% | 2.55e-03 |


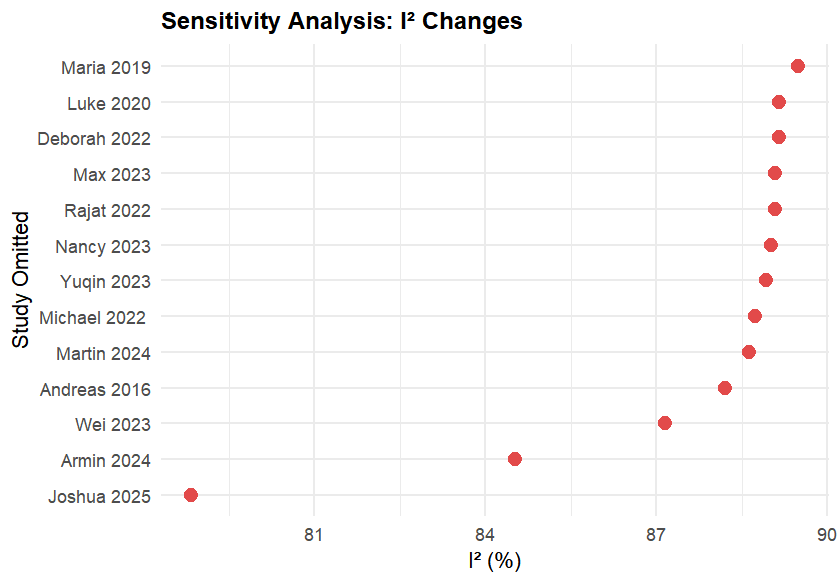


| Min_Effect | Max_Effect | Original_Effect | Range | Min_I2 | Max_I2 | I2_Range |
| --- | --- | --- | --- | --- | --- | --- |
| 0.3125475 | 0.3507271 | 0.3346554 | 0.03817957 | 78.8351 | 89.49426 | 10.65917 |

Figure S5.3 Overall Bispecific antibodies CRS≥ Grade 3


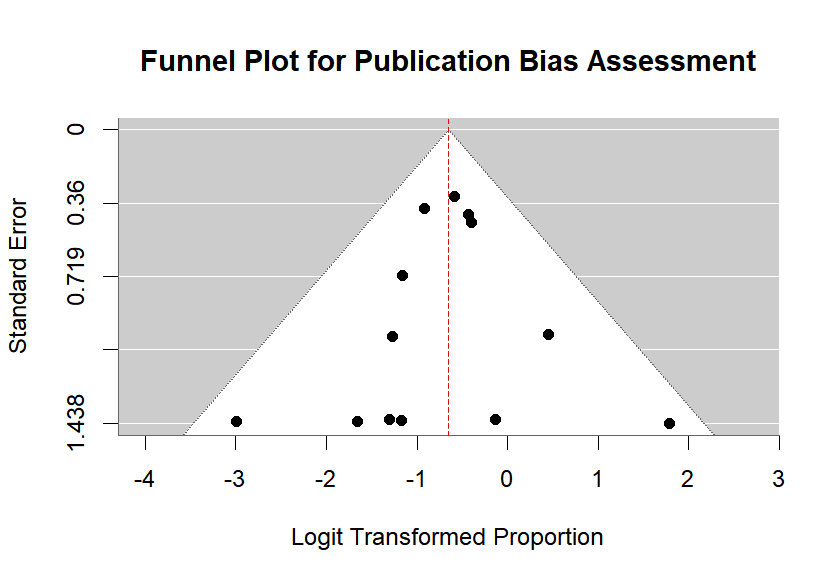


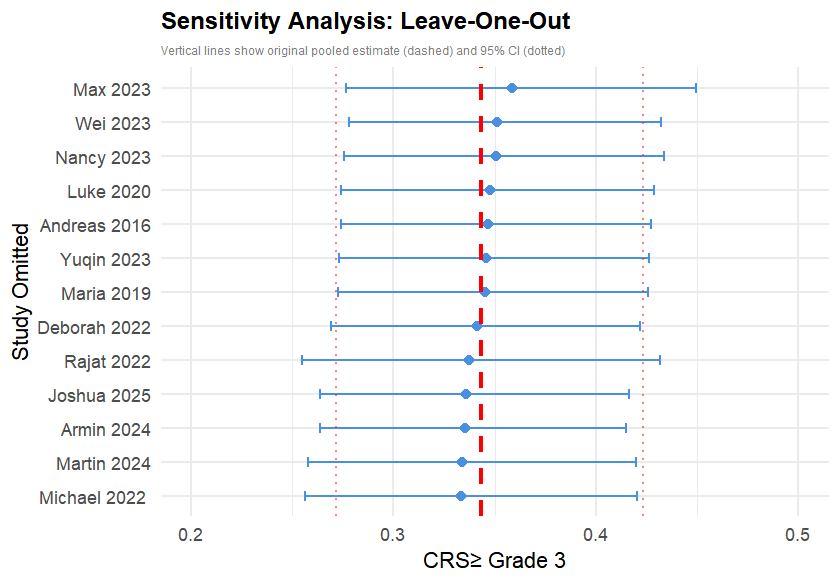


| **Study_Omitted** | **Effect_CI** | **I2** | **p_value** |
| --- | --- | --- | --- |
| Max 2023 | 0.358 [0.277, 0.449] | 0% | 2.58e-03 |
| Martin 2024 | 0.334 [0.258, 0.42] | 0% | 2.21e-04 |
| Joshua 2025 | 0.336 [0.264, 0.416] | 0% | 1.01e-04 |
| Yuqin 2023 | 0.346 [0.273, 0.426] | 0% | 2.46e-04 |
| Michael 2022 | 0.333 [0.256, 0.42] | 0% | 2.63e-04 |
| Nancy 2023 | 0.35 [0.276, 0.433] | 0% | 5.36e-04 |
| Rajat 2022 | 0.337 [0.255, 0.431] | 0% | 9.19e-04 |
| Maria 2019 | 0.345 [0.272, 0.426] | 0% | 2.34e-04 |
| Armin 2024 | 0.335 [0.264, 0.415] | 0% | 8.51e-05 |
| Deborah 2022 | 0.342 [0.269, 0.422] | 0% | 1.64e-04 |
| Luke 2020 | 0.347 [0.274, 0.429] | 0% | 3.31e-04 |
| Andreas 2016 | 0.347 [0.274, 0.427] | 0% | 2.75e-04 |
| Wei 2023 | 0.351 [0.278, 0.432] | 0% | 4.25e-04 |


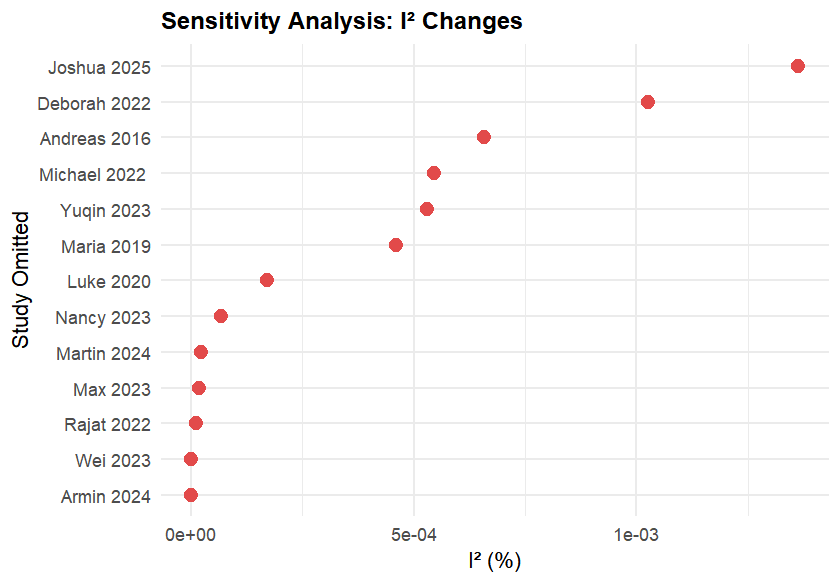


| Min_Effect | Max_Effect | Original_Effect | Range | Min_I2 | Max_I2 | I2_Range |
| --- | --- | --- | --- | --- | --- | --- |
| 0.3331716 | 0.3583912 | 0.3432662 | 0.02521957 | 0 | 0.00136318 | 0.00136318 |

Figure S5.4 Overall Bispecific antibodies Neurotoxicity≥ Grade 3


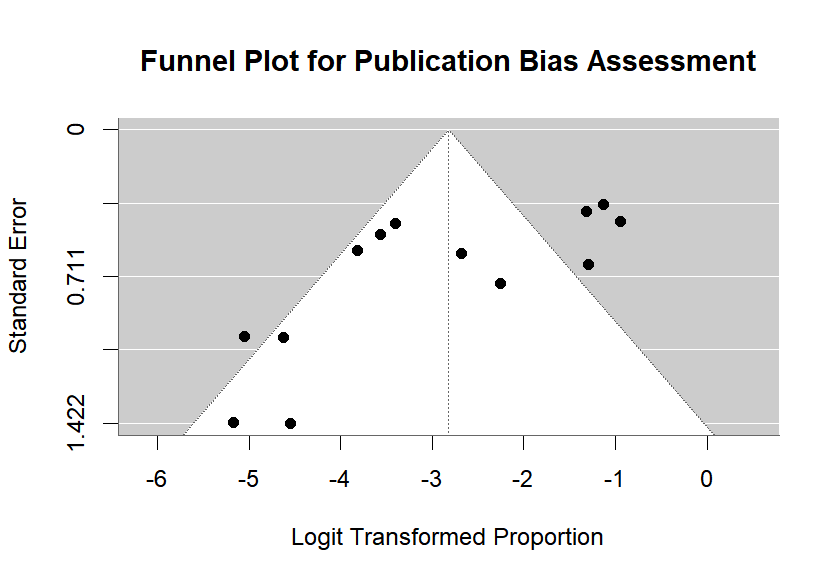


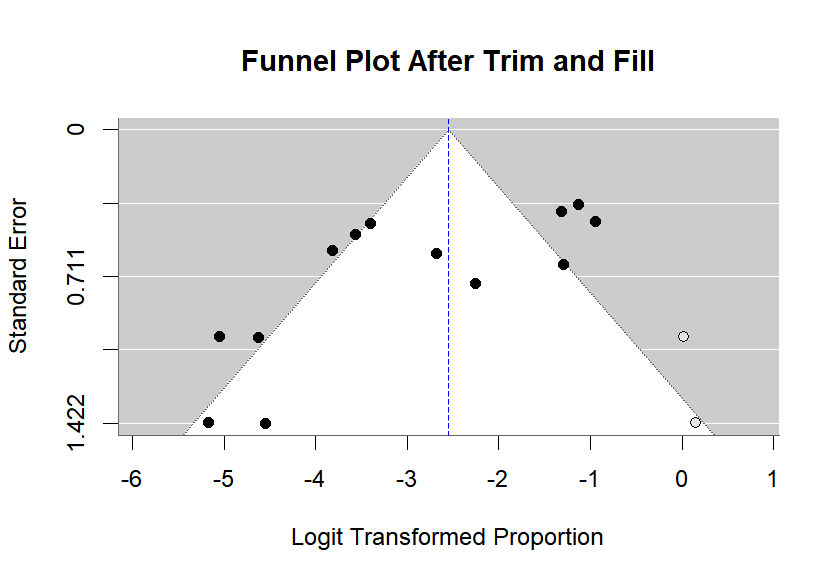


Analysis Effect_Size CI_95 p_value Tau2 I2 Egger_p_value

1 Before Imputation 0.056 [0.026, 0.118] 7.55e-12 1.671 83.3% 1.65e-03

2 After Trim and Fill 0.073 [0.033, 0.151] 1.06e-09 1.977 84.1% 2.53e-01


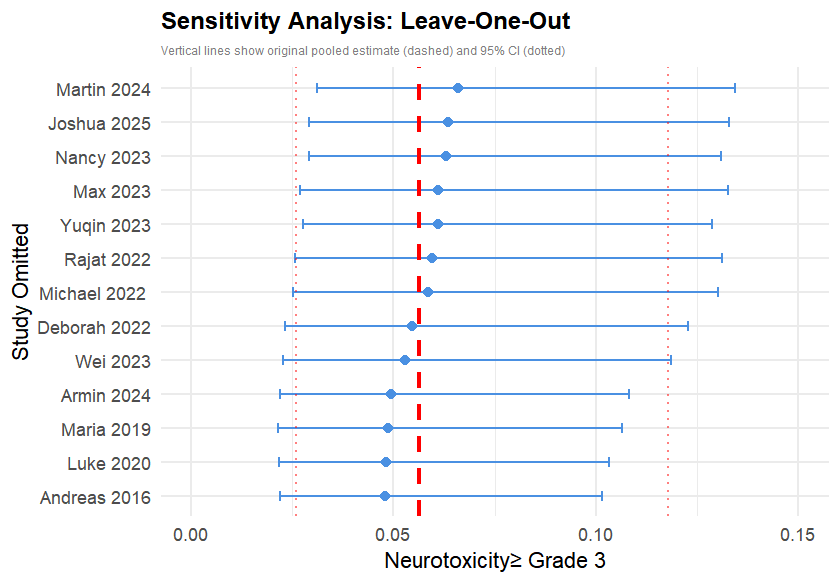


| **Study_Omitted** | **Effect_CI** | **I2** | **p_value** |
| --- | --- | --- | --- |
| Max 2023 | 0.061 [0.027, 0.133] | 83.80% | 3.80e-10 |
| Martin 2024 | 0.066 [0.031, 0.134] | 82.10% | 4.36e-11 |
| Joshua 2025 | 0.064 [0.029, 0.133] | 83.20% | 9.26e-11 |
| Yuqin 2023 | 0.061 [0.028, 0.129] | 84.20% | 7.63e-11 |
| Michael 2022 | 0.058 [0.025, 0.13] | 83.90% | 5.91e-10 |
| Nancy 2023 | 0.063 [0.029, 0.131] | 83.40% | 4.99e-11 |
| Rajat 2022 | 0.059 [0.026, 0.131] | 83.90% | 5.17e-10 |
| Maria 2019 | 0.049 [0.022, 0.106] | 81.70% | 4.91e-12 |
| Armin 2024 | 0.049 [0.022, 0.108] | 83.70% | 7.43e-12 |
| Deborah 2022 | 0.055 [0.023, 0.123] | 85% | 2.97e-10 |
| Luke 2020 | 0.048 [0.022, 0.103] | 80.40% | 1.30e-12 |
| Andreas 2016 | 0.048 [0.022, 0.101] | 80.70% | 4.64e-13 |
| Wei 2023 | 0.053 [0.023, 0.118] | 85.20% | 1.19e-10 |


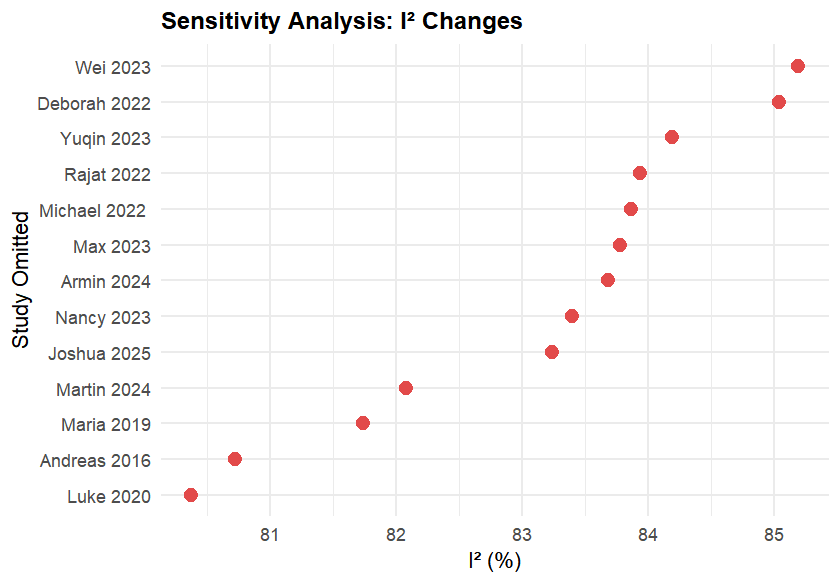


| Min_Effect | Max_Effect | Original_Effect | Range | Min_I2 | Max_I2 | I2_Range |
| --- | --- | --- | --- | --- | --- | --- |
| 0.04783014 | 0.06597086 | 0.05624032 | 0.01814072 | 80.37284 | 85.19323 | 4.820395 |

Figure S5.5 Overall Bispecific antibodies Infection≥ Grade 3


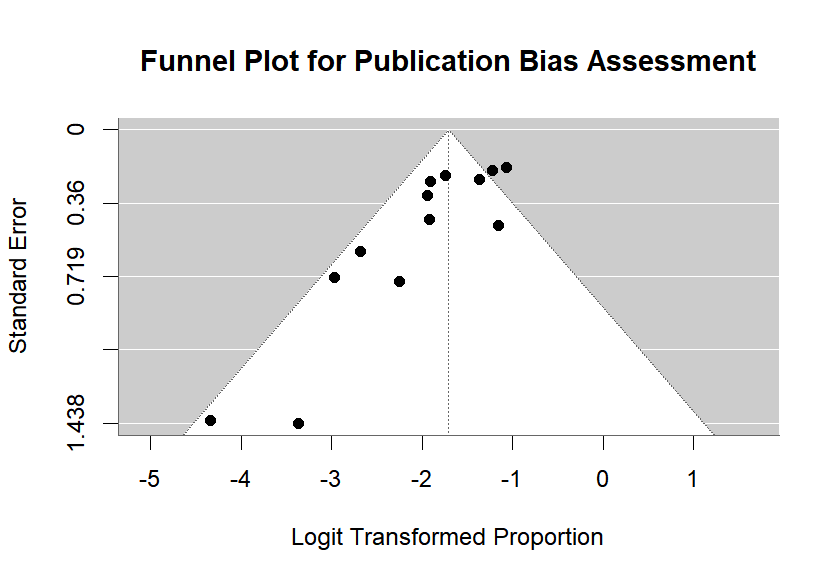


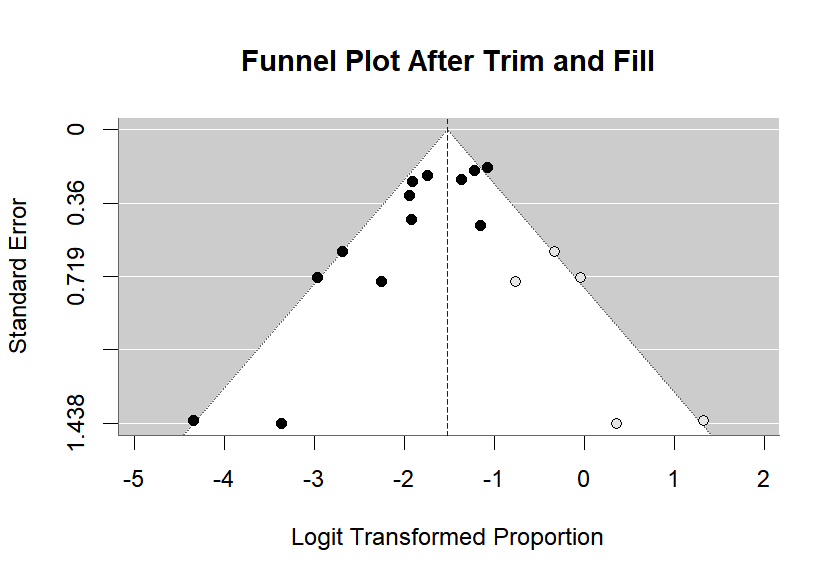


Analysis Effect_Size CI_95 p_value Tau2 I2 Egger_p_value

1 Before Imputation 0.154 [0.118, 0.197] 3.93e-28 0.144 57.9% 2.85e-04

2 After Trim and Fill 0.179 [0.138, 0.23] 1.68e-21 0.199 59.1% 9.27e-01


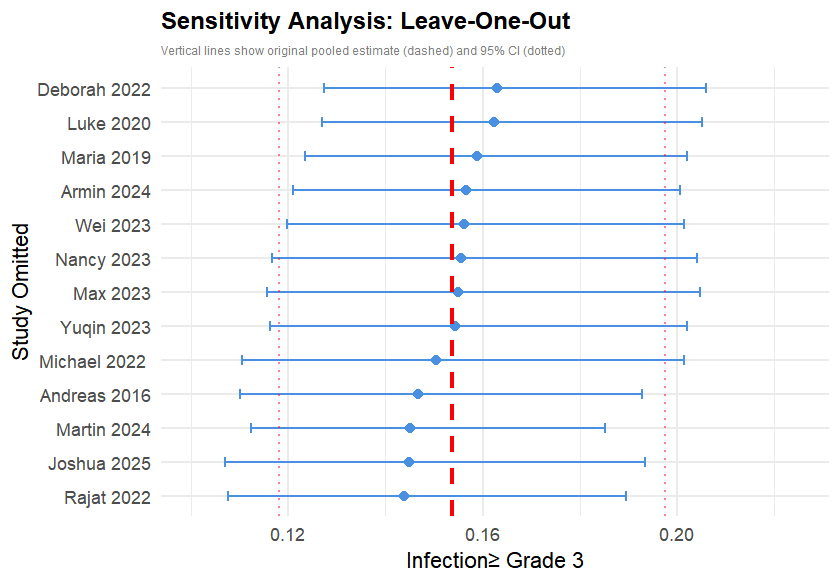


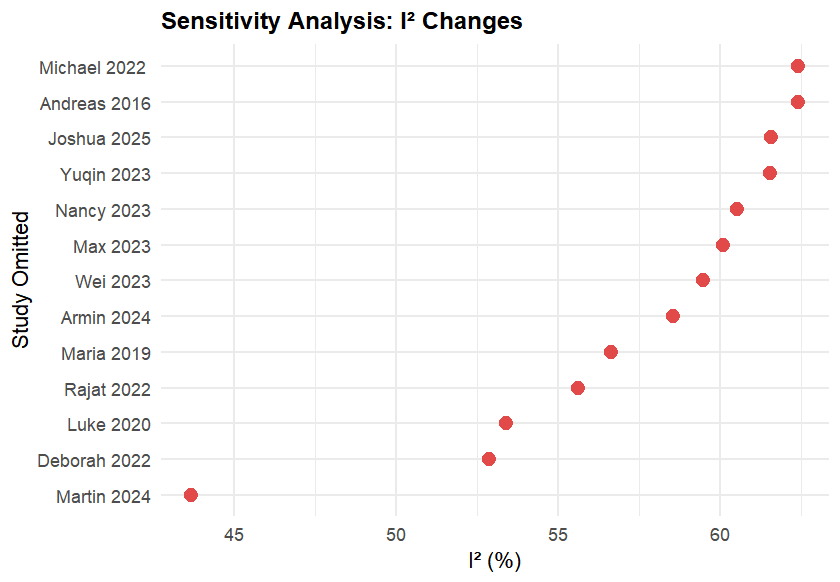


| **Study_Omitted** | **Effect_CI** | **I2** | **p_value** |
| --- | --- | --- | --- |
| Max 2023 | 0.155 [0.116, 0.205] | 60.10% | 1.10e-22 |
| Martin 2024 | 0.145 [0.112, 0.185] | 43.70% | 2.10e-32 |
| Joshua 2025 | 0.145 [0.107, 0.193] | 61.60% | 1.20e-23 |
| Yuqin 2023 | 0.154 [0.116, 0.202] | 61.50% | 2.37e-24 |
| Michael 2022 | 0.15 [0.11, 0.202] | 62.40% | 1.10e-21 |
| Nancy 2023 | 0.155 [0.117, 0.204] | 60.50% | 1.56e-23 |
| Rajat 2022 | 0.144 [0.108, 0.19] | 55.60% | 4.97e-26 |
| Maria 2019 | 0.159 [0.123, 0.202] | 56.70% | 9.24e-29 |
| Armin 2024 | 0.157 [0.121, 0.201] | 58.60% | 4.75e-28 |
| Deborah 2022 | 0.163 [0.127, 0.206] | 52.90% | 7.15e-29 |
| Luke 2020 | 0.162 [0.127, 0.205] | 53.40% | 3.77e-29 |
| Andreas 2016 | 0.147 [0.11, 0.193] | 62.40% | 1.13e-25 |
| Wei 2023 | 0.156 [0.12, 0.201] | 59.50% | 1.05e-26 |

| Min_Effect | Max_Effect | Original_Effect | Range | Min_I2 | Max_I2 | I2_Range |
| --- | --- | --- | --- | --- | --- | --- |
| 0.1437872 | 0.1629508 | 0.1536706 | 0.01916366 | 43.66806 | 62.42792 | 18.75987 |

Figure S5.6 Overall Bispecific antibodies ICANS≥ Grade 3


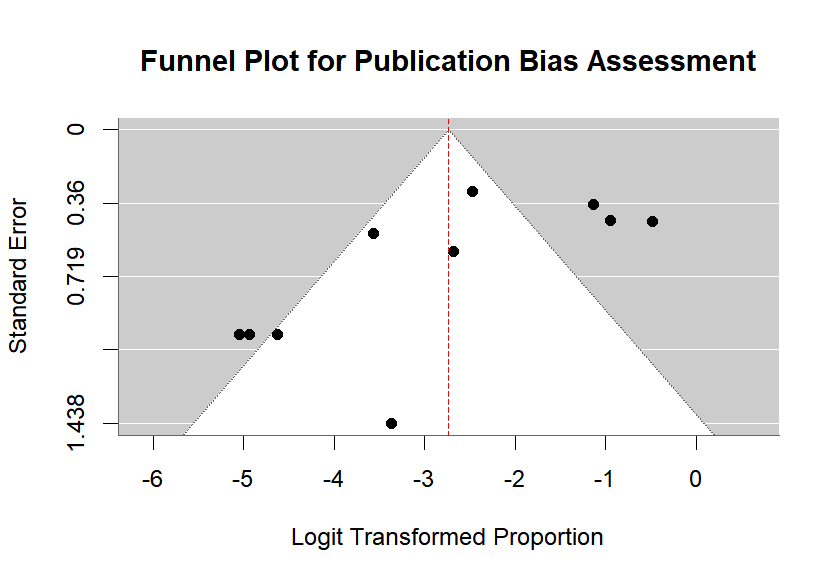


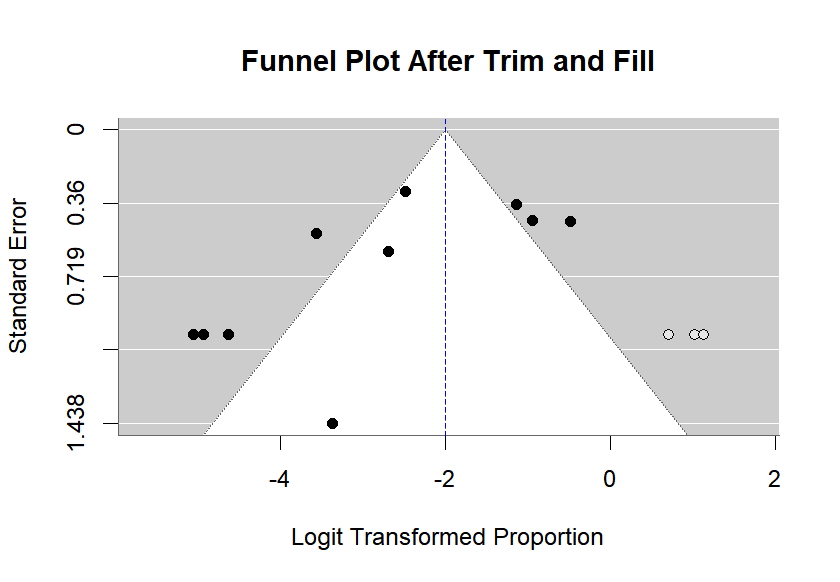


Analysis Effect_Size CI_95 p_value Tau2 I2 Egger_p_value

1 Before Imputation 0.061 [0.022, 0.155] 2.55e-07 2.286 89.1% 4.47e-03

2 After Trim and Fill 0.120 [0.041, 0.301] 6.97e-04 3.851 91.9% 6.66e-01


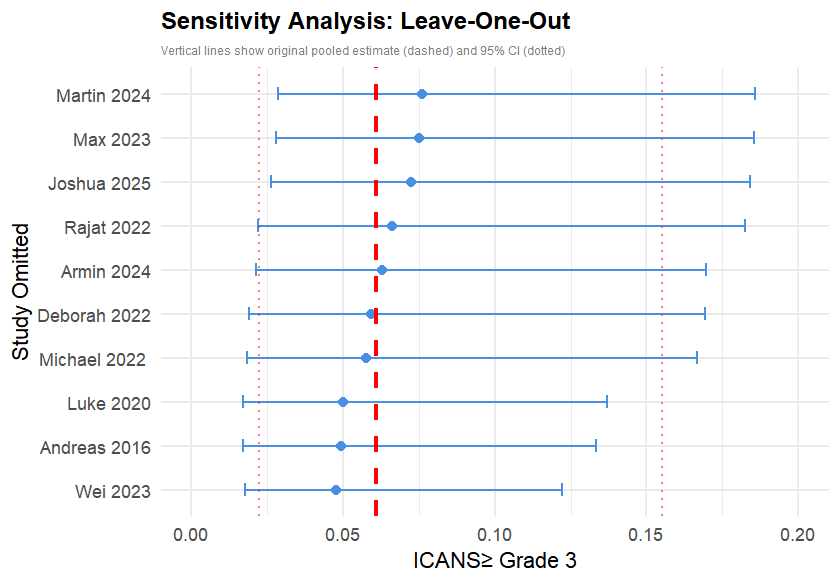


| **Study_Omitted** | **Effect_CI** | **I2** | **p_value** |
| --- | --- | --- | --- |
| Max 2023 | 0.075 [0.028, 0.185] | 88.70% | 1.87e-06 |
| Martin 2024 | 0.076 [0.029, 0.186] | 88.40% | 1.63e-06 |
| Joshua 2025 | 0.072 [0.026, 0.184] | 89.30% | 2.51e-06 |
| Michael 2022 | 0.058 [0.018, 0.167] | 88.50% | 3.90e-06 |
| Rajat 2022 | 0.066 [0.022, 0.182] | 89.80% | 6.20e-06 |
| Armin 2024 | 0.063 [0.022, 0.17] | 90.80% | 1.99e-06 |
| Deborah 2022 | 0.059 [0.019, 0.169] | 90.60% | 3.90e-06 |
| Luke 2020 | 0.05 [0.017, 0.137] | 87.70% | 1.79e-07 |
| Andreas 2016 | 0.049 [0.017, 0.133] | 88.10% | 9.55e-08 |
| Wei 2023 | 0.048 [0.018, 0.122] | 86.40% | 9.15e-09 |


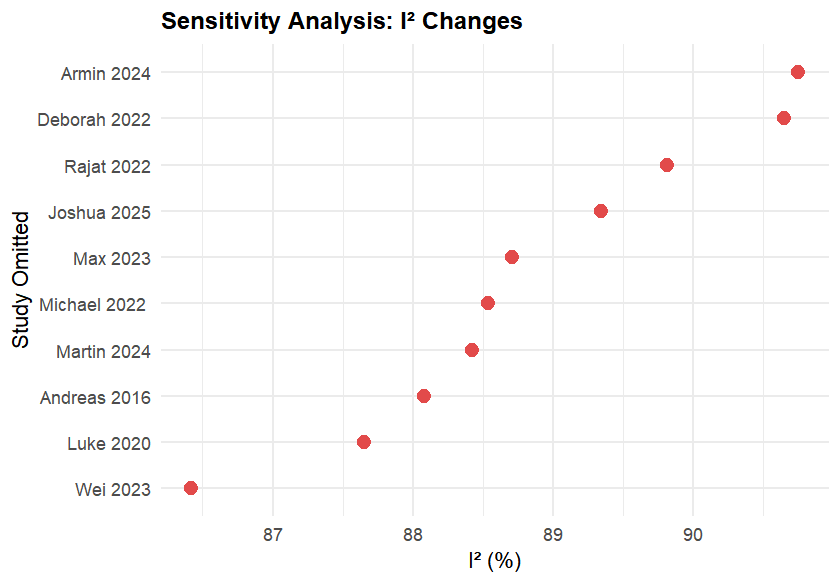


| Min_Effect | Max_Effect | Original_Effect | Range | Min_I2 | Max_I2 | I2_Range |
| --- | --- | --- | --- | --- | --- | --- |
| 0.04770353 | 0.07594316 | 0.0609384 | 0.02823963 | 86.41947 | 90.75027 | 4.330802 |

**Figure S6 The heatmap of indirect comparison**


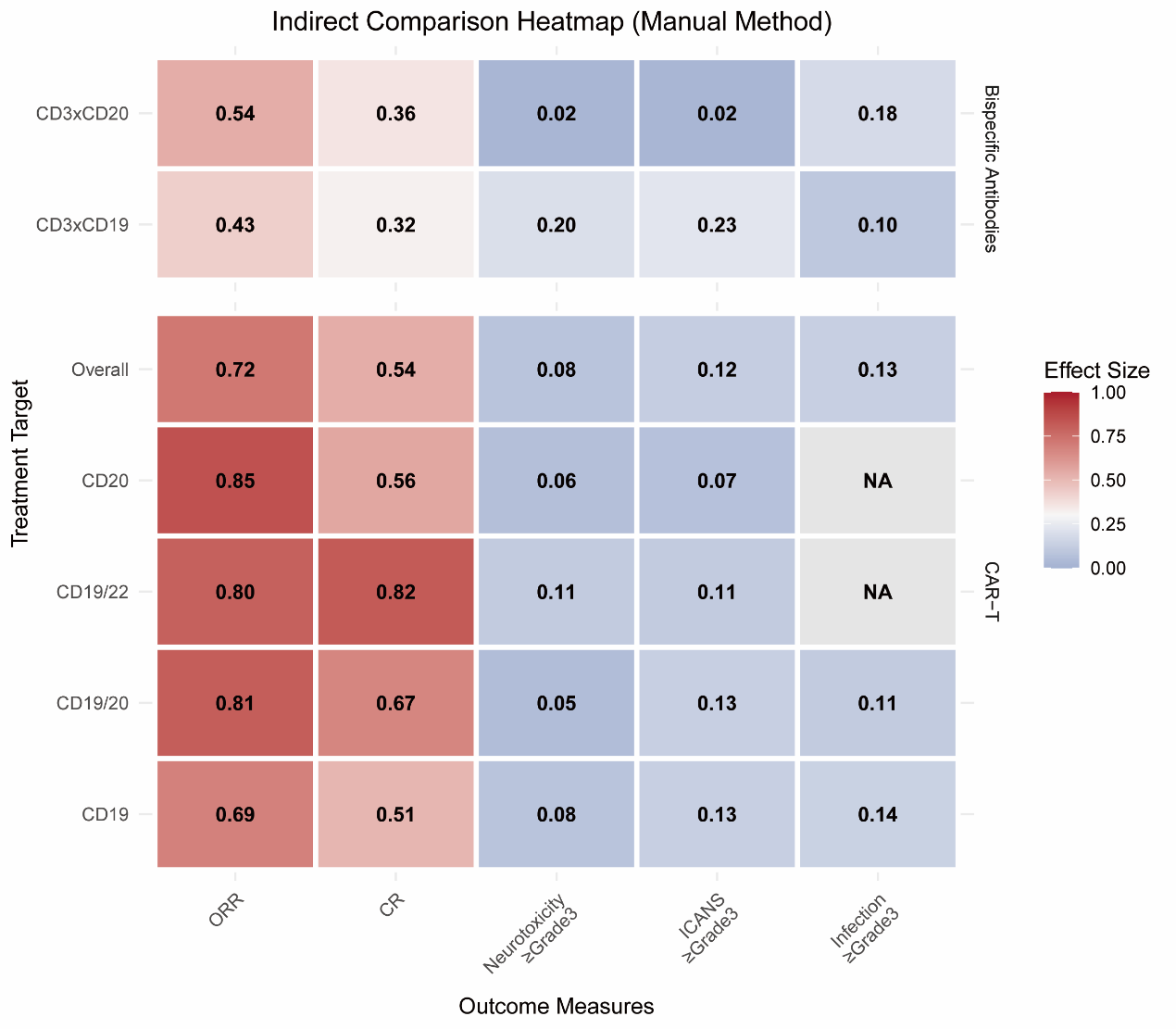


**Figure S7 Meta-regression Results**

Model Results:

estimate se zval pval ci.lb ci.ub

intrcpt 0.3329 0.2579 1.2911 0.1967 -0.1725 0.8383

Age 0.0070 0.0037 1.8859 0.0593 -0.0003 0.0143 .

Refractory 0.0002 0.0008 0.2689 0.7880 -0.0014 0.0018

Prior_treatment_lines -0.0181 0.0274 -0.6612 0.5085 -0.0718 0.0356


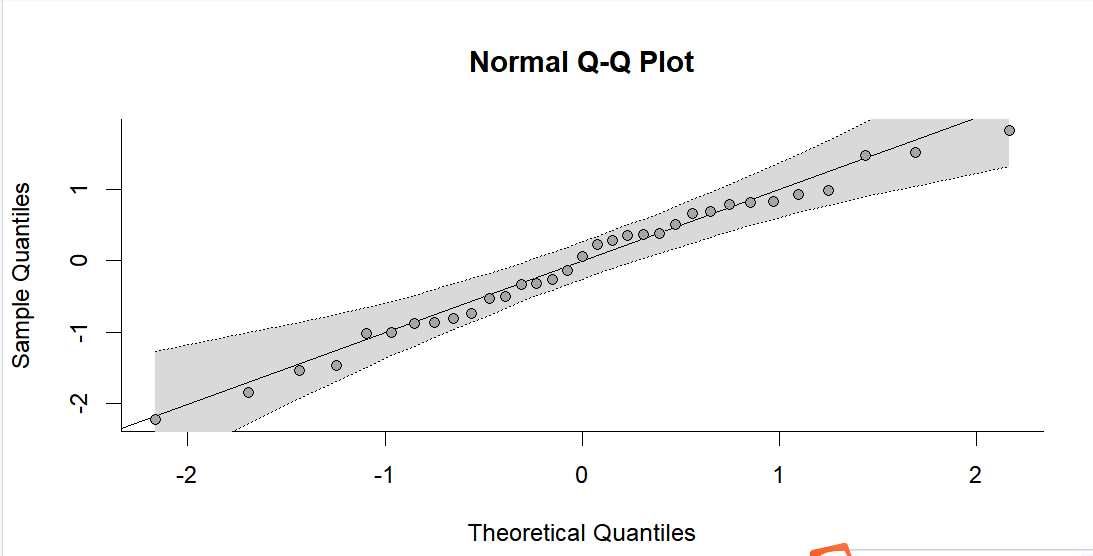

Supplement: Supplementary file 1 [file DataSheet1.docx]
